# Phylogenomic analysis of the diversity of graspetides and proteins involved in their biosynthesis

**DOI:** 10.1186/s13062-022-00320-2

**Published:** 2022-03-21

**Authors:** Kira S. Makarova, Brittney Blackburne, Yuri I. Wolf, Anastasia Nikolskaya, Svetlana Karamycheva, Marlene Espinoza, Clifton E. Barry, Carole A. Bewley, Eugene V. Koonin

**Affiliations:** 1 National Center for Biotechnology Information, National Library of Medicine, National Institutes of Health, Bethesda, MD 20894 USA; 2 Laboratory of Bioorganic Chemistry, National Institute of Diabetes and Digestive and Kidney Diseases, National Institutes of Health, Bethesda, MD 20892 USA; 3 Tuberculosis Research Section, Laboratory of Clinical Immunology and Microbiology, National Institute of Allergy and Infectious Diseases, National Institutes of Health, Bethesda, MD USA

**Keywords:** RiPPs, ATP-grasp ligase, Graspetides, Biosynthetic gene clusters, Lactones, Lactams, Functional prediction

## Abstract

**Background:**

Bacteria and archaea produce an enormous diversity of modified peptides that are involved in various forms of inter-microbial conflicts or communication. A vast class of such peptides are Ribosomally synthesized, Postranslationally modified Peptides (RiPPs), and a major group of RiPPs are graspetides, so named after ATP-grasp ligases that catalyze the formation of lactam and lactone linkages in these peptides. The diversity of graspetides, the multiple proteins encoded in the respective Biosynthetic Gene Clusters (BGCs) and their evolution have not been studied in full detail. In this work, we attempt a comprehensive analysis of the graspetide-encoding BGCs and report a variety of novel graspetide groups as well as ancillary proteins implicated in graspetide biosynthesis and expression.

**Results:**

We compiled a comprehensive, manually curated set of graspetides that includes 174 families including 115 new families with distinct patterns of amino acids implicated in macrocyclization and further modification, roughly tripling the known graspetide diversity. We derived signature motifs for the leader regions of graspetide precursors that could be used to facilitate graspetide prediction. Graspetide biosynthetic gene clusters and specific precursors were identified in bacterial divisions not previously known to encode RiPPs, in particular, the parasitic and symbiotic bacteria of the Candidate phyla radiation. We identified *Bacteroides*-specific biosynthetic gene clusters (BGC) that include remarkable diversity of graspetides encoded in the same loci which predicted to be modified by the same ATP-grasp ligase. We studied in details evolution of recently characterized chryseoviridin BGCs and showed that duplication and horizonal gene exchange both contribute to the diversification of the graspetides during evolution.

**Conclusions:**

We demonstrate previously unsuspected diversity of graspetide sequences, even those associated with closely related ATP-grasp enzymes. Several previously unnoticed families of proteins associated with graspetide biosynthetic gene clusters are identified. The results of this work substantially expand the known diversity of RiPPs and can be harnessed to further advance approaches for their identification.

**Supplementary Information:**

The online version contains supplementary material available at 10.1186/s13062-022-00320-2.

## Background

Ribosomally synthesized and post-translationally modified peptides (RiPPs) comprise a broad class of peptides with various biological activities, primarily causing cell toxicity in a wide range of organisms. Upon translation, the RiPPs are maturated and modified through diverse biochemical pathways, often employing multiple enzymes. Comprehensive reviews describing classification, structure and biosynthetic pathways of numerous RiPPs have been recently published [1, 2]. In the recent decades, the search for new antimicrobial molecules has been boosted by the development of computational tools that have helped to identify a vast number of potential RiPPs and other natural products and their corresponding biosynthetic gene clusters (BGCs) [1, 3]. Among these tools, AntiSMASH [4], RODEO [5], PRISM [6] and the recently developed RiPPER [7] are the most popular. These tools compare the sequences of proteins encoded in a query DNA with databases of custom profiles of RiPPs and their biosynthetic components and/or neighboring genes found to be adjacent to a particular RiPP class, assign the peptides to specific classes and identify known biosynthetic genes. These tools also apply class-specific “rules” or employ machine learning (RODEO) to predict new RiPPs. Genome mining for a marker gene of a particular group of RiPPs followed by “guilt-by-association” analysis of the respective gene neighborhoods has been also successfully applied for the characterization of new lantipeptides [8, 9], microcin C [10], the linear azol(in)e peptides [1, 11] and other active peptides. This approach allows identification of new auxiliary genes and prediction of new RiPPs that lack any similarity with known peptides but are conserved in several genomes. Partly, this approach is employed in the RiPPER tool [7], which however does not analyze auxiliary proteins encoded in the BGCs.

RiPPs of the graspetides class often escape automatic identification. The name of the class derives from the name of the family of enzymes, ATP-grasp ligases, that catalyze the formation of class-defining lactones and lactams in graspetide natural products [1]. In addition to graspetide biosynthesis, ATP-grasp enzymes of the same subfamily are implicated in the pathway of biosynthesis of α-guanidino acid containing peptides and bacterial head-to-tail cyclized peptides [1]. The ATP-grasp ligases involved in the biosynthesis of graspetides are difficult to distinguish, without phylogenetic analysis, from other families of ATP-grasp enzymes involved in RiPP natural product biosynthesis, examples of which include the ligases that catalyze N-terminal to C-terminal cyclization in head-to-tail bacterial peptides and the α-guanidino amino acid-peptide ligase [1, 12]), and from the RimK enzymes that catalyze the maturation of the ribosomal protein S6 [13]. This difficulty with the identification of the relevant enzymes and the fact that the sizes and sequences of the precursors are extremely diverse complicates prediction of graspetides using the above-mentioned tools and suggest that a comparative genomic approach could be more productive.

**Fig. 1:**
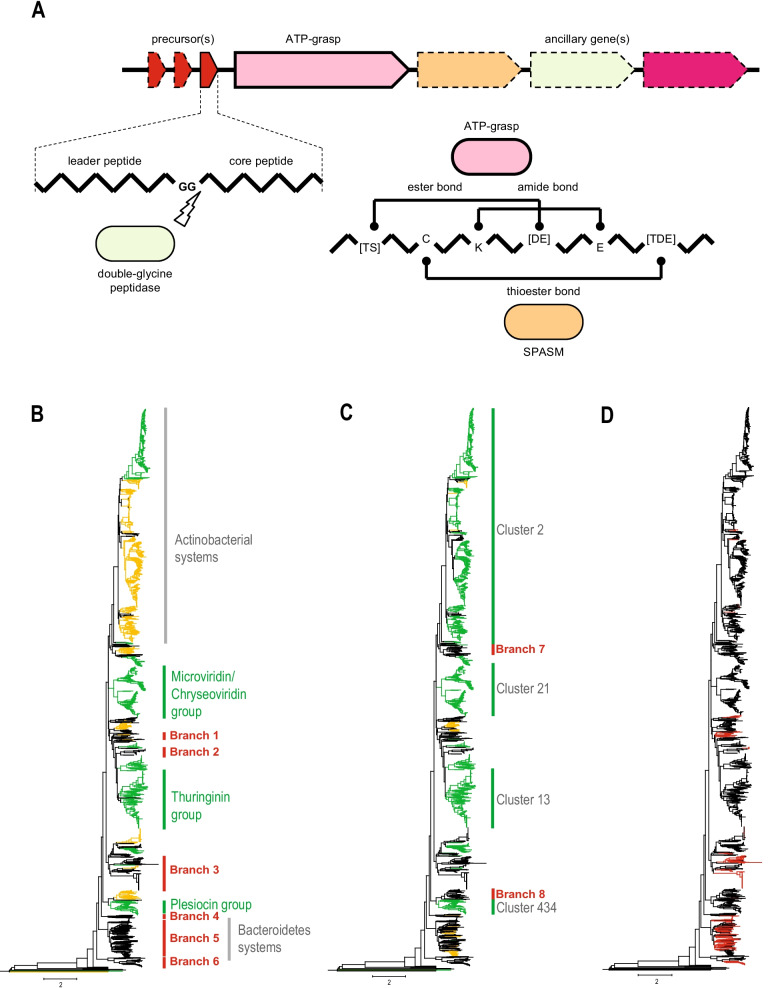
Graspetide processing and comparison of the known graspetide BGCs with BGCs identified in this work. **A** General scheme of graspetide processing. Key features of the organization of graspetide biosynthetic gene clusters and subsequence processing of the precursor peptide are depicted. *SPASM* peptide maturase of the SPASM family, a radical SAM enzyme. **B** Comparison of ATP-grasp sequences identified in this work with ATP-grasp sequences reported by Iyer et al. [23] and Lee et al. [24]. ATP-grasp tree reconstructed in this work is schematically shown. Clades corresponding to experimentally characterized graspetides are marked on the right side of the tree. Two large subfamilies of ATP- (Actinobacterial and Bacteroidetes) mentioned in the work by Iyer et al. [23] are shown in gray. Yellow, ATP-grasp sequences that are at least 50% identical to and overlap by at least 80% of the protein length with those from Iyer et al. [23]. Green, ATP-grasp sequences that are at least 50% identical to and overlap by at least 80% of the protein length with those from Lee et al. [24] or from both studies. Black, the remaining ATP-grasp sequences that were not analyzed previously. Branches 1 to 6 selected for in depth characterization in this work are indicated on the right. Detailed information can be found in the Additional file 8: Table S1. **C** Correspondence between precursor sequences and ATP-grasp enzyme phylogeny. Known precursors are mapped on the same ATP-grasp tree if they are present in respective loci. Precursor cluster number corresponding to experimentally characterized graspetides and to Actinobacterial clade are indicated on the right by gray. Green—precursor similar to those reported in Lee et al. [24] paper or matching to one of the 12 motifs delineated in the same work (see "Methods" for detail). Yellow—precursors known either from Iyer et al. [23] work or annotated as such using respective PSSM (position specific score matrices) from CDD (conserved domain database). Black—ATP-grasp loci where known precursor peptides were not identified. Branches 7 and 8 selected for in depth characterization in this work are indicated on the right. Detailed information can be found in the Additional files 8, 10: Table S1 and S3. **Ds** Precursors identified in this work. Precursors identified in this work (red) are mapped on the same ATP-grasp tree if they are present in respective loci. Detailed information can be found in the Additional file 8: Table S1

The founding and best-studied member of the graspetide class of RiPPs is microviridin, which was originally discovered in *Microcystis* and has been shown to disrupt the molting process in growing *Daphnia pulicaria* feeding on this cyanobacterium [14]. The gene for the microviridin codes for a protein precursor that is cleaved by an unknown peptidase to release the 13 amino acid (aa) peptide known as the core peptide. The peptidase that cleaves the precursor most likely belongs to the double glycine protease family because the precursors typically contain a GG-motif preceding the core peptide and the gene coding for this peptidase is often found in the vicinity of the microviridin related BGCs [15]. Subsequently, it has been shown that microviridin is a serine protease inhibitor [16]. Microviridin has a cage-like structure formed by two ω-ester linkages between the side chain carboxyl group of Asp/Glu and the hydroxyl group of Ser/Thr, and one amide linkage between the δ-carboxyl group of Glu and the ε-amino group of Lys [17, 18]. The formation of these linkages is catalyzed by two paralogous ATP-grasp enzymes encoded in the respective loci [18]. Subsequently, graspetides were characterized including marinostatin [19] and chryseoviridin [20], distant homologs of microviridin, and plesiocin and thuringinin that lack sequence similarity to the former two peptides or to each other [21, 22]. Plesiocin and thuringinin require a single ATP-grasp enzyme to make all the linkages [21, 22]. So far, all characterized graspetides are protease inhibitors [1]. The general scheme of the organization of the graspetides loci and processing of the precursor and core peptide is shown on the Fig. 1A.

In 2009, a comprehensive analysis of the ATP-grasp superfamily revealed a large group of enzymes predicted to be involved in RiPP biosynthesis [23]. In this work, 12 families of RiPPs have been identified. Most of these families are currently deposited in the PFAM database as Inhibitor_I10 or microviridin (PF12559), “Strep_pep” (PF14404), “Actino_peptide” (PF14408), “Bacteroid_pep” (PF14406), “Herpeto_peptide” (PF14409) and “Frankia_peptide” (PF14407). Since 2009, numerous additional bacterial genomes have been sequenced, so a reanalysis of this vast family of ATP-grasp enzymes appears to be timely in order to characterize the expanding diversity of graspetides and mechanisms of their biosynthesis. In a recent study, Ahmed et al. focused on the reanalysis of ATP-grasps linked to microviridin-like peptides [15]. They identified 308 distinct graspetides and proposed to classify this family into three groups based on sequence similarity, the presence of processing signals and the number of core peptides. In 2020, a more comprehensive analysis of 2005 ATP-grasp enzymes associated with RiPP biosynthesis has been published [24]. This study identified 12 groups of graspetides including 9 with novel consensus core motifs. Furthermore, the linkages for core peptides of 6 distinct groups have been characterized experimentally, and the high specificity of ATP-grasp enzymes for their cognate groups of peptides has been demonstrated [24].

The rationale for this study was to expand the analysis reported by Lee et al. [24], and, in particular, include more distant ATP-grasp subfamilies that were not covered in that study. We were also interested to complement the work of Lee et al. [24] by analysis of auxiliary genes and to explore the evolution of the recently characterized chryseoviridin families, in an attempt to understand the origin and evolution of these precursors. To our knowledge, such analysis has not been previously attempted for graspetides. In the present work, we employed a larger database of complete and draft genomes to search for ATP-grasp enzymes. Combined with sensitive computational methods, this allowed us to substantially expand the diversity of graspetide precursors and predict new precursor peptides in several taxa of uncultured bacteria. We also predicted several previously undescribed auxiliary genes encoded in the respective operons and found evidence of duplication and horizontal gene exchange being the principal factors in the evolution of the chryseoviridin family.

## Results and discussion

The initial analysis of the *Chryseobacteria* spp. strains MEBOG6 and MEBOG7 genomes that were sequenced from the environment with the goal of discovering potential novel bioproducts resulted in the identification of two microviridin BGCs (Additional file 1: Figure S1). Considering that microviridin was the founding member of the graspetide class and because we were particularly interested in the evolution of MEBOG BGC homologs we used the ATP-grasp proteins encoded in these loci as queries to initiate a PSI-BLAST search for homologs in the genomic database (see "Materials and Methods" section). The search resulted in the identification of 2761 ATP-grasp proteins. We aligned these sequences, reconstructed a phylogenetic tree and mapped on the tree the sequences with at least 80% similarity (and at least 80% length coverage) with those from Iyer et al. [23] and Lee et al. [24] (Fig. 1B, Additional files 11, 12, Additional file 8: Table S1). This comparison revealed 6 branches that have not been examined in-detail in these two previous studies (Fig. 1B). Thus, we analyzed in depth the neighborhoods of the ATP-grasp genes related to these six branches.

Next, we clustered all the proteins encoded in the 2521 loci coding for 2761 ATP-grasp proteins (some microviridin-like loci encompass two ATP-grasp genes) and compiled the initial set of graspetide precursor candidates. These set included all proteins that were 150 amino acids or less in length and that were encoded in the immediate vicinity of ATP-grasp genes (first or second neighbor in both directions) (Additional file 9: Table S2). The resulting initial set of candidates consisted of 6270 proteins (Additional file 10: Table S3). The clusters were mapped to known precursors both by using BLAST and by identification of conserved motifs described by Lee et al. [24] (Fig. 1C; see “Methods” section for details). This comparison identified two additional branches lacking known precursor peptides, prompting us to examine the neighborhoods for ATP-grasp genes from these two branches in detail (Additional file 8: Table S1).

In addition to the analysis of the putative precursors in the genomic neighborhoods corresponding to the eight branches of poorly characterized ATP-grasp enzymes, all clusters that included five or more candidate precursors were subject to further case-by-case sequence and neighborhood analysis. These analyses included additional PSI-BLAST and HHpred searches, examination of the respective neighborhoods (checking whether the members of the clusters stably and specifically associated with ATP-grasp enzymes), identification of double glycine motifs, and assessment of the conservation of the residues known to be involved in the formation of lactones and lactams, namely Ser, Thr, Asp, Glu and Lys. Altogether, through these analyses we identified 1739 (59 clusters) “known” and 435 (115 clusters) new graspetide precursors (Fig. 1D, Additional file 10: Table S3). In the next two sections, we discuss the diversity of the identified precursors and specifically describe loci corresponding to the eight poorly characterized branches in the ATP-grasp tree.

### Distinct and shared features of the RiPP precursors

**Fig. 2:**
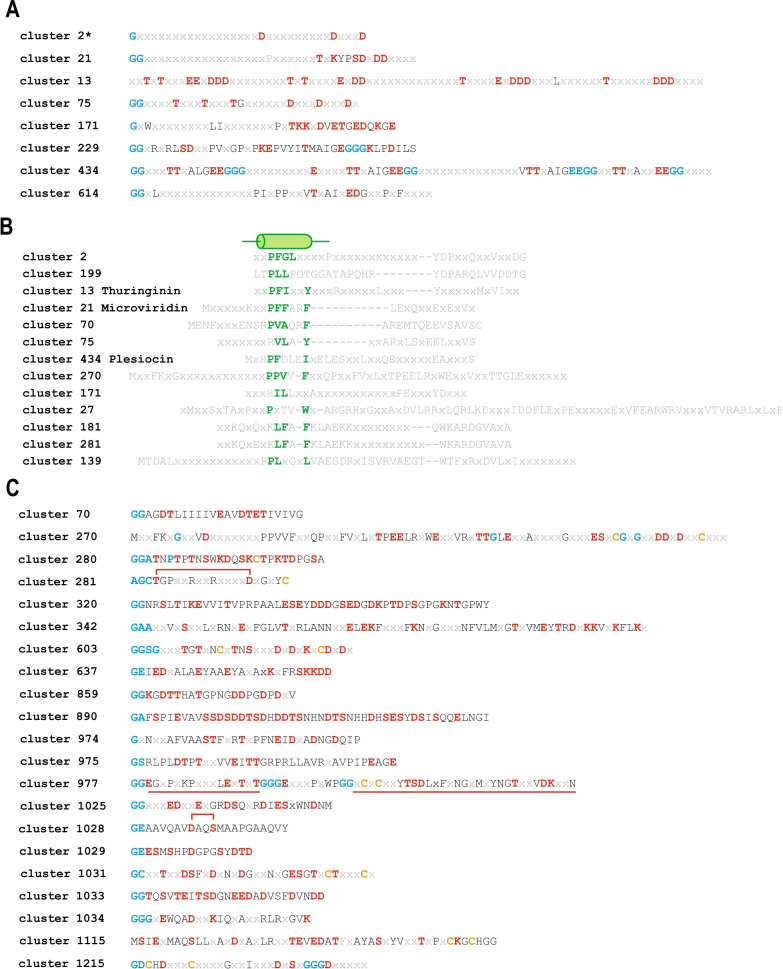
Leader region motifs and new core peptides. **A** Conserved features of largest clusters of known core peptides. Consensus sequences of core peptides are shown for clusters of precursors with more than 10 representatives. The consensus for the multiple alignment of each cluster was determined as described in "Material and Methods" section. Conserved positions that could be involved in the formation of ester or amide bonds are highlighted by red, and variants of the GG-motifs are highlighted by blue. The asterisk indicates that for cluster 2, the diversity of the core peptides is so high that only aspartates required to form lactam bonds are conserved throughout the alignment, whereas the positions of hydroxyl-containing amino acids (Thr and Ser) are not conserved. Gaps in the consensus sequence were removed for compactness. **B** Conserved features of leader regions. Consensus sequences of leader peptides are shown for largest clusters of precursors. Consensus for multiple alignment of each cluster was determined as described in “Material and Methods” section. Conserved positions corresponding to a putative alpha helix which is interacting with ATP-grasp are highlighted by green. The alpha helix indicated schematically above the consensuses according to its position in microviridin precursor [26]. **C** Conserved features of core peptides identified in this work. Consensus sequences of core peptides are shown for clusters of precursors with 5 or more representatives. Consensus for multiple alignment of each cluster was determined as described in “Material and Methods” section. Conserved positions that can be involved in formation of ester or amide bonds are highlighted by red, variants of GG-motifs are highlighted by blue; conserved cysteine that could be involved in formation of thioether bonds are highlighted by orange. Red brackets show an only potential ester bond for two core peptides. Multiple potential cores in core region are underlined. Gaps in the consensus sequence were removed for compactness

As it could be expected, the largest clusters, namely, cluster 2 (958 proteins), cluster 13 (250 proteins) and cluster 21 (185 proteins) consist of “known” precursor sequences (Figs. 1C, 2A**,** Additional file 10: Table S3). Given that we employed a sensitive sequence comparison procedure for clustering, we expected that some precursors that classified into different groups based on the core peptide motifs defined by Lee et al. [24] would fall into the present work because they might share a common leader peptide sequence. Indeed, this appears to be the case for all three largest clusters. Cluster 2, for instance, combined proteins containing sequences matching the core peptide motifs of groups 8, 9 and 11, but share the leader peptide (Additional file 2: Figure S2A, Additional file 8: Table S1). However, the majority of the proteins in cluster 2 (504) do not match any motif described by Lee et al. (24), so the core peptides in these precursors represent “hidden” novelty (Additional file 10: Table S3). For example, precursors from *Salinispora arenicola* strains (eg. KB905534.1) have a core peptide signature “TxxxTxxDxxxxDD”, which is distinct from the 12 motifs described by Lee et al. [24], but encompass a similar leader peptide, and the corresponding ATP-grasp enzymes group with others from this cluster (Additional file 2: Figure S2; Additional file 8: Table S1). Furthermore, ATP-grasps encoded next to cluster 199 precursors belong to the same Actinobacterial branch of the tree and these precursors contain a leader peptide similar to those in cluster 2, but the respective core peptides do not match any of the established motifs (Fig. 1B, C, Additional file 2: Figure S2, Additional file 8: Table S1). These observations suggest that the ATP-grasp enzymes of this branch can tolerate variations of the order and distances between amino acids involved in lactone and lactam linkages but require distinct features of the leader peptide.

Cluster 21 corresponds to the most thoroughly studied precursors of the microviridin/chryseoviridin group. In PSI-BAST searches, almost all of these grouped with precursors identified by Lee et al. [24], but only 121 of the 185 perfectly matched the motif of group 1 delineated by Lee et al. [24], again highlighting some flexibility of the ATP-grasp enzymes modifying the respective core peptides.

Precursors in cluster 13 are of special interest because they encompass several repeats of the core peptide of groups 3, 5 or 6 from Lee et al. [24]. The motifs of these groups share some similarity in the general arrangement of Thr, Glu and Asp residues although the distances between the conserved residues differ. Furthermore, some proteins containing the motif of group 3 also match the motif of group 4, including thuringinin itself, making it difficult to use the motifs for the group classification and for predicting the connectivity between amino acids in the core peptides (Additional file 2: Figure S2). The precursors with core peptide motifs of these three groups also have a similar leader sequence as noted previously [21] (Additional file 2: Figure S2). Our procedure combined all these groups together with several other sequences in cluster 13, a classification that better corresponds to the ATP-grasp phylogeny than the split of these precursors into multiple groups (Fig. 2A, Additional file 8: Table S1).

Leader peptides are known to play an important role in the processing of many RiPPs [25]. Microviridin-like precursors contain the highly conserved “PFFARFL” sequence in the leader, which interacts with the middle subdomain of ATP-grasp, and this interaction is necessary to initiate catalysis [15, 20, 26]. We analyzed consensus sequences of leader peptides of all largest clusters of identified precursors and found that, in all these clusters, the leader region contained several conserved residues, typically, often within the 30 N-terminal residues (Fig. 2B). The most prominently conserved amino acid in these regions is a proline followed by two hydrophobic amino acids, and an aromatic residue that is often present at the end of the conserved region, resulting in a “Phhx(1,2)h” motif, where “h” is a hydrophobic amino acid and “x” is any amino acid (Fig. 2B). We identified this motif in ~ 50% of both the known and new precursors detected in this work (Additional file 10: Table S3). As pointed out above, the sequences of the leader peptide closely correspond to the major branches of the ATP-grasp phylogeny (Additional file 8: Table S1). Most likely, these conserved leader regions play the same role as the “PFFARFL” motif in the microviridins, activating the ATP-grasp enzymes.

Many RiPPs contain the functionally important double-glycine (GG) motif, which represents a cleavage site for the double-glycine peptidase of the C39 family, which cleaves the leader off the core peptide [27]. Given that this peptidase cleaves numerous other RiPPs, it is not typically encoded in the ATP-grasp loci, and the identity of the enzyme involved in the cleavage of graspetides remains unknown. Overall, the GG-motif can be found in about half of the precursors (both known and new), which is not particularly surprising because multiple variations of this motif have been identified (Additional file 10: Table S3). Therefore, in the present study, conservation of at least one small amino acid residue (Gly, Ala, Ser, Cys, Glu) in the N-terminal region of a protein cluster alignment was used as an important feature to predict new precursors. Typically, in a cluster of precursors, there is only one conserved doublet of small amino acids, typically, including at least one glycine (Fig. 2C). Only in two cases, cluster 270 and cluster 1115, this motif could not be identified. In the case of cluster 977, there are three “GG” motifs which, if all cleaved, could result in two different modified peptides (Fig. 2C).

Overall, we predicted 2174 precursors, of which 1739 (59 clusters) were classified as “known” (with the caveats discussed above) and 435 (115 clusters) were “new” (Additional file 13). Thus, the present analysis roughly tripled the known diversity of RiPP precursors although, not unexpectedly, the newly predicted precursors typically belonged to smaller families than those previously described (Additional file 10: Table S3). For larger clusters (5 proteins or more) of new precursors, we mapped amino acids that could potentially form ester or amide linkages in the predicted core peptide consensus sequences (Fig. 2B). In most cases, however, the connectivity between amino acids could not be predicted because there are multiple candidate amino acids present in these regions that could potentially form lactam or lactone linkages. Only in two cases, cluster 281 and cluster 1028, the formation of a single ester bond is theoretically possible. Many new precursors contain one or more conserved cysteine residues, which might serve as sulfur donors for the formation of thioether bonds by radical SAM enzymes [28]. In addition, 1162 candidate precursors were not examined in detail. Among these, there were 49 (40 clusters, mostly, consisting of a single sequence) candidates that are encoded in a putative operon with an ATP-grasp enzyme and for which both the “GG” and “Phhx(1,2)h” motifs were identified within the N-terminal 30 amino acids. Therefore, most likely, these are RiPP precursors with at least 40 distinct core peptides (Additional file 10: Table S3).

### Analysis of eight groups of previously unexplored ATP-grasp loci

As indicated above, we identified 8 branches on the ATP-grasp tree that corresponded to poorly characterized ATP-grasp loci, of which many lacked identifiable known precursors. We examined these loci in more-details. Branches 1 and 2 consist of ATP-grasp sequences from draft genomes of a large group of (mostly) uncultured bacteria from the candidate phyla radiation (CPR) [29]. These bacteria typically have small genomes and cell sizes, belong to deep branches within the bacterial subtree of life [29], and are thought to be parasites or symbionts of other bacteria [30]. The functions of graspetides of CPR bacteria identified in this work remain to be elucidated. They might serve as an “outsourced” offense system benefiting the host, or could contribute to the interactions with the host, and/or to competition among different CPR bacteria leading to superinfection exclusion. These potentially novel biological phenomena clearly merit further study.

**Fig. 3:**
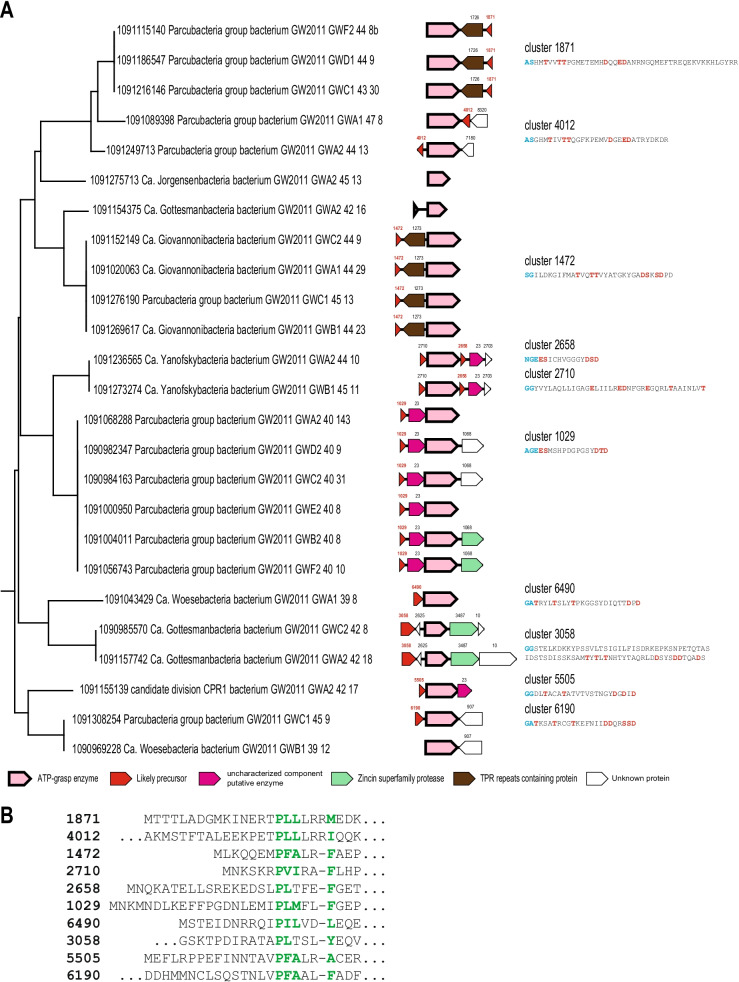
Predicted precursors and loci organization in ATP-grasp branch 1. **A** ATP-grasp loci organization and precursor sequences for branch 1. ATP-grasp subtree for branch 1 (see Fig. 1B) is shown on the left. ATP-grasp loci are mapped to respective genomes. Genes are shown by block arrows, roughly to scale. Putative precursors are shown by red, other genes are colored according to the homology and the key is shown at the bottom of the tree. Cluster number is indicated for all genes, except those coding for ATP-grasp proteins. The consensus sequences for core peptides are shown on the right and colored as follows: conserved amino acids that could be involved in formation of ester or amide bonds are highlighted by red, GG-motifs are highlighted by blue, amino acids conserved in the leader sequence are highlighted by green. **B** Leader peptide region of precursors for branch 1. Cluster number is indicated on the left. Conserved positions are highlighted by green

For the loci from branch 1, we predicted 10 clusters of precursor peptides (Fig. 3A). In each of these clusters, the sequences are almost identical, but the clusters share no identifiable sequence similarity with each other. However, all these putative precursors share the “Phhx(1,2)h” motif, typically, within the first 30 amino acids (Fig. 3B). A putative cleavage site was also detectable in these predicted precursors although, in most cases, it was not the canonical GG. We cannot rule out that the actual cleavage site is different, especially considering that we identified two distinct subfamilies (clusters) of metallopeptidases encoded in several of these loci. Although the core peptides are different, most of them contain the signature TxxxTx(6–10)Dx(1–4)D. However, both putative core peptides in candidatus Yanofskybacteria bacterium lack this motif, again suggesting that, even within one branch of ATP-grasp enzyme, substantial variation of the core peptide structure is possible. In addition to the genes for putative precursor, these loci often encompass other genes; in particular, those encoding TPR repeat-containing proteins and proteins of unknown function from cluster 23 that are discussed below in the “Associated genes” section (Fig. 3A).

**Fig. 4:**
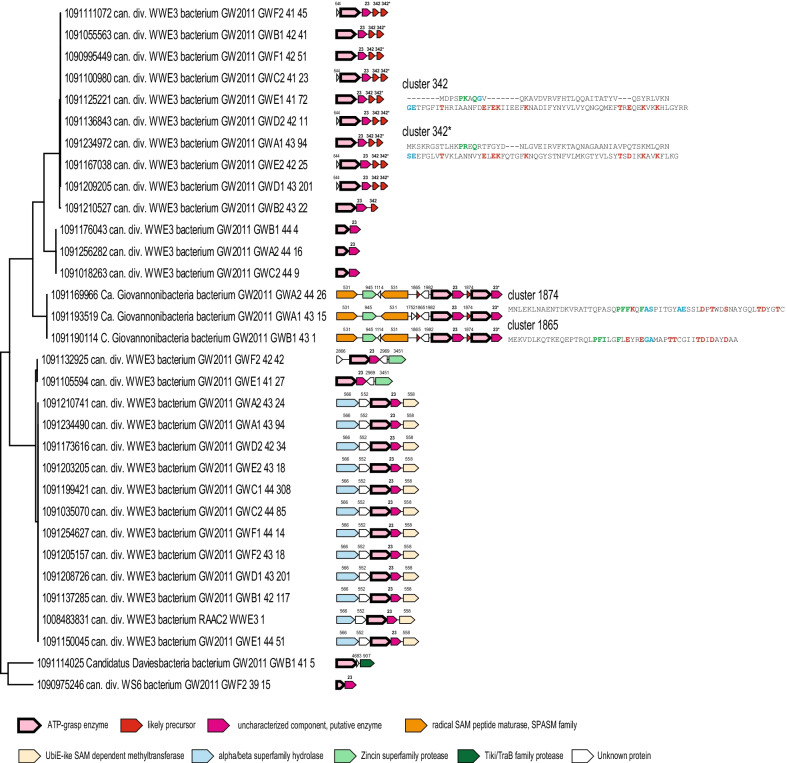
Predicted precursors and loci organization in ATP-grasp branch 2. ATP-grasp subtree for the branch 2 is shown on the left. ATP-grasp loci are mapped to respective genomes. Genes are shown by block arrows, roughly to scale. Putative precursors are shown by red, other genes are colored according to the homology and the key which is shown at the bottom of the tree. Cluster number is indicated for all genes, except those coding for ATP-grasp proteins. The consensus sequences for precursors are shown on the right and colored as follows: conserved amino acids that could be involved in formation of ester or amide bonds are highlighted by red, GG-motifs are highlighted by blue, amino acids conserved in leader sequence are highlighted by green

Branch 2 loci and precursors are even more diverse than those from Branch 1 (Fig. 4). Cluster 342 precursors are encoded as a tandem of two divergent paralogs and show sufficient sequence diversity to enable prediction of the leader and the core peptide (Figs. 2B, 4). Two putative precursors are also encoded in candidatus Giovannoni bacteria loci (Fig. 4). These precursors contain the “Phhx(1,2)h” motif, but otherwise have no common features. We could not confidently identify any precursor genes in any of the other loci associated with Branch 2. The precursors might be small proteins that were missed by ORF prediction methods, or alternatively, could be encoded far from the ATP-grasp enzymes. As in the case of Branch 1, all Branch 2 loci encode a cluster 23 family protein. Many loci also encode a SAM radical peptide maturase of the SPASM family or a UbiE-like methyltransferase, which could be involved in further peptide modifications [31]. Additionally, two distinct peptidases of the zincin superfamily, Tiki/TraB family and possibly alpha/beta hydrolase family are likely co-expressed with the respective ATP-grasp enzymes and could be involved in the cleavage of the leader or in further maturation of the modified peptide.

**Fig. 5:**
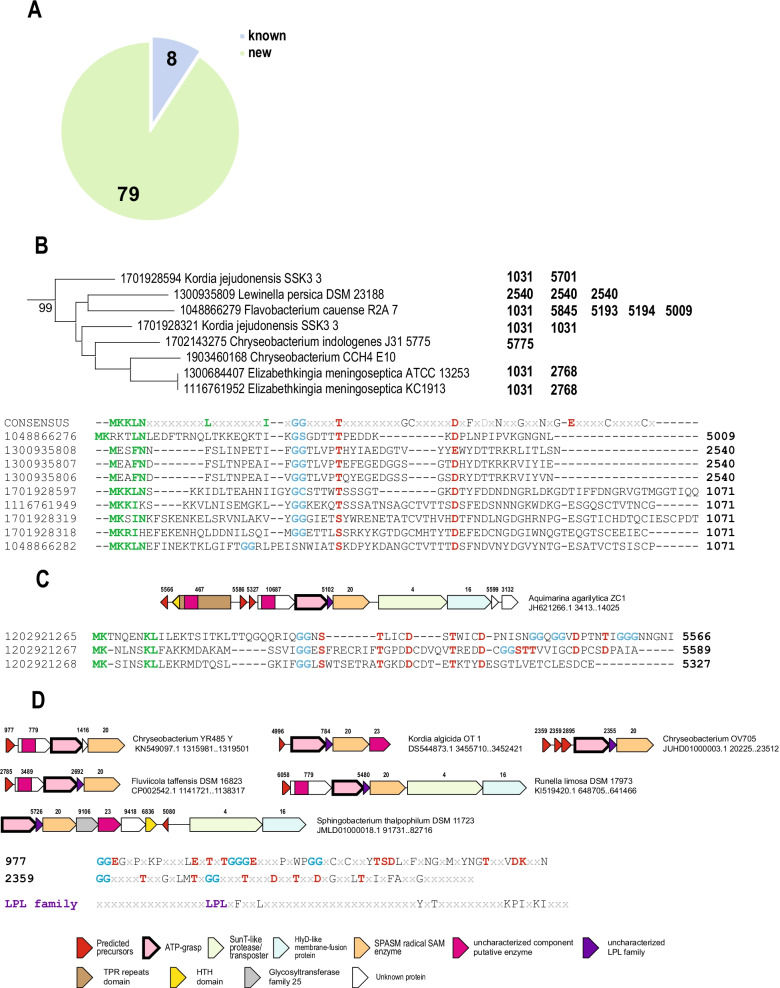
Predicted precursors and loci organization in ATP-grasp branch 5. **A** Known precursor clusters versus new precursor clusters for branch 5. **B** Example of ATP-grasp subtree for Bacteroidetes with diverse precursors encoded in respective loci. The support value for the subtree is indicated. All cluster numbers identified in respective loci are indicated on the right. Manual alignment of most of the precursors in these loci is shown below. Protein local identifiers for the precursors are indicated on the left and the cluster numbers on the right. Amino acids highlighted as follows: conserved amino acids that could be involved in formation of ester or amide bonds are highlighted by red, GG-motifs are highlighted by blue, amino acids conserved in leader sequence are highlighted by green. **C** Example of diverse precursors in the locus of *Aquimarina agarilytica*. Locus organization is shown. Genes are shown by block arrows, roughly to scale. Genes are colored according to the homology and the key which is shown at the bottom of the Figure. Cluster number is indicated for all genes, except those coding for ATP-grasp proteins. Genbank accession and coordinates of the locus are indicated on the right. Manual alignment of the precursors is shown below. Local protein identifiers and respective cluster number are provided for each protein on the left and right respectively. Amino acids highlighted as follows: conserved amino acids that could be involved in formation of ester or amide bonds are highlighted by red, GG-motifs are highlighted by blue, amino acids conserved in leader sequence are highlighted by green. **D** Examples of selected loci from the branch 5. Selected loci organizations are shown. Genes are depicted by block arrows, roughly to scale. Genes are colored according to the homology and the key which is shown at the bottom of the Figure. Cluster number indicated above the arrows is provided for all genes, except those coding for ATP-grasp proteins. Genbank accession and coordinates of the locus and the name of the organism are indicated on the right of the locus schematics. Consensus sequences for two clusters precursors is shown below, cluster number are indicated on the left. Amino acids highlighted as follows: conserved amino acids that could be involved in formation of ester or amide bonds are highlighted by red, GG-motifs are highlighted by blue. Consensus sequences for LPL family is shown below the precursor sequences and the signature “LPL” motif is highlighted by purple

Branch 5 is the largest (190 loci) among the poorly characterized groups of ATP-grasps and is highly specific for Bacteroides. Several of these BGCs have been described by Iyer et al. [23], and typically, in addition to the ATP-grasp, encode a SPASM family enzyme. The latter seems to be specifically associated with small peptide modifications and can be involved in the formation of carbon–carbon, carbon–oxygen or carbon–sulfur (thioether) bonds [28, 31, 32]. The thioether bonds typically involve a cysteine residue as a donor of sulfur and a carbon atom of the acceptor Asn, Thr or Asp residue. Only several precursors from Bacteroides were identified by Iyer et al. [23]. Based on this analysis, a single PSSM was generated in the PFAM database (pfam14406, “Ribosomally synthesized peptide in Bacteroidetes”) and two more PSSMs (TIGR04139 and TIGR04149) were derived for putative precursors encoded next to the SPASM family peptide maturase [31]. Only 8 clusters of precursors from branch 5 loci set were identified as “known” based on sequence similarity with one of these PSSMs, whereas 79 more were detected upon examination of the respective loci (Fig. 5A). The cause of this unexpected diversity of the predicted precursors became apparent when we mapped the precursors clusters from these loci to the ATP-grasp tree (Additional file 3: Figure S3). Even closely related genomes with closely similar ATP-grasp enzymes were found to encode apparently unrelated or distantly related precursors, often several in the same locus. As an example, a small subtree from Branch 5 is shown in Fig. 5B. Most of the predicted precursors in the respective loci are small proteins with an easily identifiable and often canonical GG-motif that is typically encoded upstream of the ATP-grasp gene. Using several iterations of PSI-BLAST, it was in some cases possible to identify additional regions of sequence similarity between some but not all of the precursors (Fig. 5B). One of such regions is the putative leader sequence “MKKL” and another region is in the core peptide and includes strictly conserved Thr/Ser position and an Asp/Glu position (Fig. 5B). The latter two residues are most likely linked in the mature peptide. Precursors from cluster 1071 also show conservation of three cysteine residues that could be involved in the formation of thioether bonds by the SPASM family enzyme. Conserved cysteines are also typical of other precursors associated with branch 5 (Additional file 10: Table S3). In many loci with several precursors, their sequences could not be reliably aligned using available methods, but nevertheless shared similar features identifiable upon detailed examination. For example, in the ATP-grasp locus of *Aquimarina agarilytica*, there are three precursors from three distinct clusters; however, in addition to the GG-motif, we detected a pattern of amino acids likely involved in ester bond formation (Fig. 5C). Interestingly, some of the predicted precursors contain several GG-motifs, so that some of the regions between these motifs that contain Thr, Ser and Asp residues potentially might form distinct mature peptides. Each precursor additionally contains a cysteine residue that can be a sulfur donor for further modification.

Additional examples of precursors containing several GG-motifs, with a high diversity even within the same cluster, are shown in Fig. 5D. The combinations of the residues potentially involved in ester or amide bonds formation differ from the examples discussed above. These observations imply extensive chemical diversity of the precursors associated with branch 5 despite the high similarity among the ATP-grasp sequences, even without further modifications that most likely occur given that all these loci encode SPASM family enzymes (Fig. 5D).

In addition, almost all branch 5 loci encode a protein belonging or homologous to cluster 23, which we discuss in detail below, and a small protein, which we dubbed LPL family, after the signature conserved motif of these proteins (Fig. 5D, Additional file 2: Figure S2). Using several iterations of PSI-BLAST, we identified 116 proteins (41 distinct cluster) that belong to the LPL family, which appears to be specific for branch 5. Although the LPL family proteins are small and are strongly associated with ATP-grasp loci, they seem to lack features, such as conservation of the GG-motif, that would identify them as potential precursors. The fact that these LPL proteins are always encoded directly upstream of the SPASM family enzymes suggests that they are auxiliary components, possibly analogous to pyrroloquinoline quinone (PQQ) biosynthesis protein PqqD, which is a chaperone required for proper positioning of precursor peptide PqqA in the modifying SPASM family enzyme PqqE [28, 33]. In the loci where LPL family was not identified, genes encoding unrelated small proteins are located in the exact same position and thus can be predicted to play the same role as the LPL proteins. It cannot be ruled out that these proteins interact with ATP-grasp enzymes, especially considering that the LPL motif is reminiscent of the Phh[× 1–2]h motif and that in precursors of branch 5 the latter motif is absent.

**Fig. 6:**
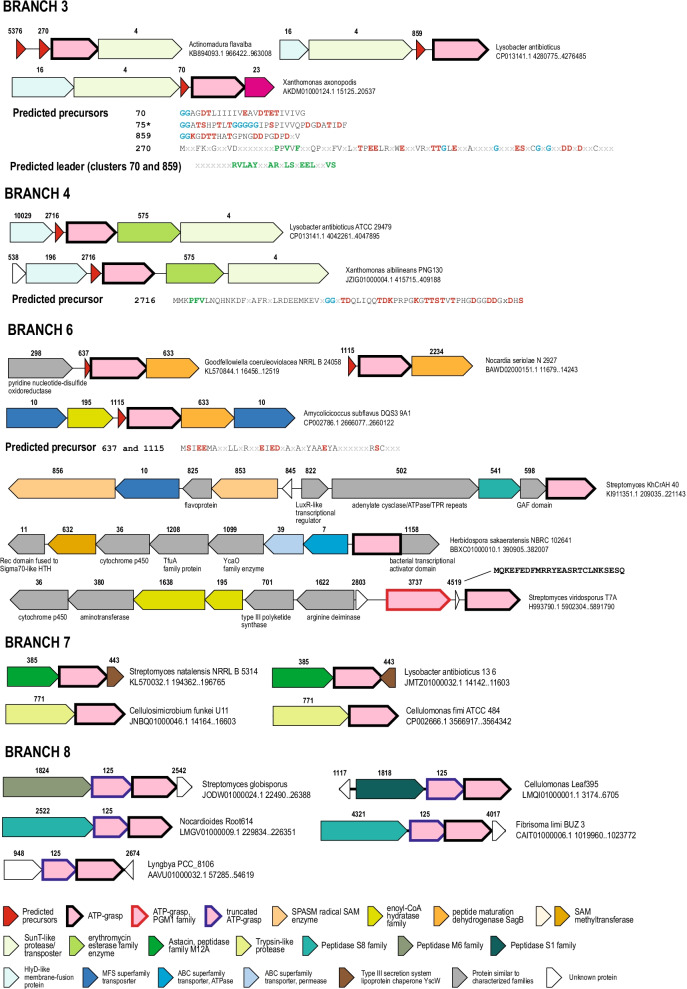
Predicted precursors and loci organization in ATP-grasp branches 3, 4, 6, 7 and 8. Selected loci organization are shown for each branch. Genes are depicted by block arrows, roughly to scale. Genes are colored according to the homology and the key which is shown at the bottom of the Figure. Cluster number indicated above the arrows is provided for all genes, except those coding for ATP-grasp proteins. Genbank accession and coordinates of the locus and the name of the organism are indicated on the right of each locus schematics. Consensus sequences for clusters of putative precursors are shown below loci schematics for Branch 3, 4 and 6, cluster numbers are indicated on the left. Amino acids highlighted as follows: conserved amino acids that could be involved in formation of ester or amide bonds are highlighted by red, GG-motifs are highlighted by blue, conserved amino acids in putative leader peptides are highlighted by green. Asterisk indicates cluster 75 which is known from previous studies but shown here for comparison because it is associated with Branch 3. Amino acid sequence for putative precursor in the *Streptomyces viridosporus* T7A locus is shown

Most genomes from branch 3 belong to either gammaproteobacteria or actinobacteria. Most diverse actinobacterial precursors belong to cluster 270 or its distant homologs (clusters 4099, 5178, 5376, 6087). The proteins contain a conserved motif in the leader region similar to the “Phhx(1,2)h” motif (Figs. 2A, 6), but the identity of the GG cleavage motif, and consequently, the core peptide region are unclear despite the fact that double-glycine peptidase of cluster 4 is encoded in many of these loci, so that it could be expected that the “GG” site or its analogs should be conserved in the precursors (Fig. 6). In multiple *Xanthomonas* genomes from this branch, precursors belong to either cluster 70 or cluster 75 (Fig. 6). Some of these precursors are associated with ATP-grasps from a neighboring branch, which includes several precursors of group 8 identified by Lee et al. [24], but none of the predicted precursors of cluster 75 match the group 8 consensus and instead display a distinct pattern of amino acids in the predicted core peptide region (Fig. 6). Another group of predicted precursors from *Lysobacter* species (gammaproteobacteria) belongs to cluster 859 and contains yet another configuration of amino acids implicated in the formation of ester or amide bonds. However, as in other cases discussed above, the similarity between the leader regions was detectable even for precursors with highly dissimilar core peptide regions (Fig. 6).

With branch 4 loci, we largely failed to predict precursors although an assortment of small proteins is frequently encoded in the ATP-grasp putative operons. However, these small proteins are not conserved and most often lack any of the canonical “GG” or “Phhx(1,2)h” motifs or other identifiable sequence patterns. The only conserved precursor, in which we detected these motifs, is cluster 2716 that consists of two closely similar sequences from different gammaproteobacterial species (Fig. 6). Considering that the loci organization in these genomes is nearly identical and, in addition to the precursor and ATP-grasp, includes the double-glycine peptidase and another enzyme distantly related to erythromycin esterase (cluster 575) these loci might have recently spread by horizontal transfer (Additional file 9: Table S2). The function of erythromycin esterase homolog is unclear. These proteins might be involved either in additional modification of the peptide or confer the host’s resistance for this peptide.

In most loci of branch 6, we could not identify precursor genes either, with the exception of the clusters 367 and 1115 that we found to be distantly related to each other (Fig. 6). These predicted precursors lack obvious counterparts of with the “GG” motif or the “Phhx(1,2)h” motif. Most loci encoding these precursors, in addition to the ATP-grasp enzymes, also code for a nitroreductase superfamily enzyme that is most closely similar to the peptide maturation dehydrogenase SagB involved in the maturation of streptolysin S [34]. These enzymes have been identified as components of several RiPP- associated BGCs where they catalyze the formation of thiazole and oxazole heterocycles between cysteine (sulfur donor) and threonine or serine (oxygen donor) and the side chain of a preceding amino acid [1, 35]. Most of the other loci encoding the branch 6 ATP-grasp enzyme are highly complex and encode several enzymes previously found to be associated with different modification steps of various RiPPs maturations pathways. These include a SPASM family radical SAM enzyme [28], aminotransferases, adenylate cyclases and polyketide synthase (Fig. 6). In particular, ATP-grasp enzymes of branch 6 are associated with ATP-dependent cyclodehydratases of the YcaO family and TfuA, which is often associated with YcaO family enzymes, and is involved in the hydrolysis of thiocarboxylated ThiS as a sulfur donor, enhancing the affinity of YcaO for the thioamidation substrate [1, 36]. YcaO family proteins are known to be involved in the biosynthesis of bottromycins, linear azole-containing peptides, thioamitides and thiopeptides, but to the best of our knowledge, association of YcaO with ATP-grasp has not been so far reported despite recently published genome mining results [1, 37]. Also notable is the association of branch 6 ATP-grasps enzymes with type III polyketide synthetases, dehydratases, amino- and amidinotransferases and other proteins recently shown to be involved in the biosynthesis of the antibiotic pheganomycin [12] (Additional file 4: Figure S4). One step of this pathway is catalyzed by a distantly related ATP-grasp ligase PGM1, which links a small core peptide with a nonproteinogenic amino acid, (S)-2-(3,5-dihydroxy-4-hydroxymethyl)phenyl-2-guanidinoacetic acid [12]. In the neighborhood from *Streptomyces viridosporus T7A *(Fig. 6), ATP-grasp from branch 6 is encoded along with PGM1, but all three genes of pheganomycin biosynthesis, those for non-ribosomal peptide synthetase, radical family SAM enzyme and C-methyltransferase, are absent suggesting that the product of this pathway is distinct from pheganomycin (Fig. 6, Additional file 8: Table S1, Additional file 4: Figure S4). The association of ATP-grasps with some of the genes from these loci, such as dehydrogenase and radical SAM enzymes, was noticed previously [23], but the relationships between these ATP-grasps have not been established. Here we show that ATP-grasps from branch 6 play a role in complex pathways of modified peptide biosynthesis along with many different modifying enzymes that might jointly introduce a distinct chemical modification remaining to be characterized.

We were unable to predict any precursors neither for branch 7 nor for branch 8. Most of the loci associated with these branches include genes for peptidases, surprisingly, of several unrelated families (Fig. 6). Therefore, it seems likely that these peptidases provide peptides for modification by ATP-grasp enzymes by cleaving some other cellular proteins distinct from typical precursors and possibly encoded outside the ATP-grasp loci. The link to proteases in these loci is so strong that it seems justified to hypothesize that the uncharacterized protein of cluster 948 often encoded in these loci in cyanobacteria might be a peptidase, too (Fig. 6, Additional file 8: Table S1). The loci of branch 7 often encode a homolog of Type III secretion system lipoprotein chaperone YscW, but not other components of the Type III secretion system [38]. The loci of branch 8 typically encode a truncated ATP-grasp protein that contains only the two N-terminal domains and thus is catalytically inactive.

### Genes frequently associated with ATP-grasp loci

To analyze functional associations of ATP-grasp enzymes, we examined the genes (identified by the cluster number) that belong to the same directons (closely spaced genes transcribed in the same direction) with an ATP-grasp. We computed raw frequency and weighted frequency (to normalize for potential redundancy of the ATP-grasp sequences) for each cluster found in the analyzed directons and considered those 18 that were present in more than 20 loci and in at least 1% of the independent observations (weighted frequency) to be non-randomly associated with ATP-grasp (Table 1). As it could be expected, double glycine peptidase and RiPP specific ABC transporter (cluster 4) topped the list ranked by weighted frequency because these genes were broadly dispersed over different branches in the ATP-grasp tree, and therefore, ranked higher than O-methyltransferase that is encoded in many more loci but is largely specific for the Actinobacterial branch (Fig. 1B, Additional file 8: Table S1). Two more families were also frequently found in the ATP-grasp loci, namely the SPASM radical SAM enzyme and HlyD-family periplasmic protein involved in the secretion of mature peptides [39]. These four protein families have been found to be associated with ATP-grasp and accordingly discussed previously [23, 31]. Most of the remaining families listed in Table 1 have not been previously considered in the context of functional connections with ATP-grasp and RiPPs, and thus were of interest although they are typically specific for a few branches in the ATP-grasp tree (small values of weighted frequency). Cluster 18, for example, consists of uncharacterized membrane proteins specific for the thuringinin biosynthetic loci. Considering that it is mostly present in loci that lack known peptide transport systems, it seems likely cluster 18 proteins are involved in peptide transport. We analyzed in greater detail cluster 23 because these proteins are encoded in many loci discussed above. Iterative PSI-BLAST searches identified 264 (25 distinct clusters) cluster 23 homologs, including proteins of cluster 149, placing these proteins, taken together, among the three most abundant families associated with graspetide BGCs (Table 1). Many of these proteins contain a predicted signal peptide and the typical lipoprotein attachment site, a conserved cysteine following the signal peptide, suggesting that they are secreted and targeted for lipidation by lipoprotein diacylglyceryl transferase [40]. Examination of the multiple alignment, revealed several conserved positing including histidine and arginine, suggesting that these proteins could have some enzymatic activity (Additional file 2: Figure S2D). The specific functions of these proteins remain to be determined.

**Table 1:** The most common protein families associated with ATP-grasp enzymes

Cluster number	Weighted frequency (%)	Number of occurrences	CDD PSSM	Description
Cluster 4	0.19	433	COG1132	Double glycine peptidase (C39 family) and RiPP specific ABC transporter
Cluster 3	0.18	717	TIGR04188	Protein-L-isoaspartate(D-aspartate) O-methyltransferase, RiPPs specific
Cluster 20	0.13	185	TIGR04193	SPASM domain peptide maturase
Cluster 16	0.09	191	TIGR01843	Type I secretion membrane fusion protein HlyD-family, macrolide-specific efflux protein, periplasmic
Cluster 18	0.04	198		Uncharacterized membrane protein
Cluster 23	0.03	150		Uncharacterized protein
Cluster 14	0.03	85	pfam13581	Histidine kinase-like ATPase domain
Cluster 146	0.02	40		Uncharacterized protein, same family as cluster 23
Cluster 8	0.02	68	pfam13560	Helix-turn-helix domain, often fused to uncharacterized protein DUF5753
Cluster 5	0.02	84		TPR-repeats containing protein
Cluster 9	0.02	30	COG1028	Short chain dehydrogenase
Cluster 163	0.02	31	pfam14905, TIGR04056	Outer membrane protein beta-barrel family; TonB-linked outer membrane protein, SusC
Cluster 145	0.02	40	TIGR04500	Peptidyl-prolyl cis–trans isomerase family protein
Cluster 24	0.02	50	pfam00561	Alpha/beta hydrolase
Cluster 7	0.02	25	pfam00005	ABC transporter, ATPase binding component
Cluster 40	0.02	86		Uncharacterized membrane protein
Cluster 11	0.02	92	COG0745	DNA-binding response regulator, OmpR family
Cluster 6	0.01	39	COG1309	DNA-binding transcriptional regulator, AcrR

Several protein families associated with ATP-grasp loci are likely involved in the regulation of gene expression in these loci. Histidine kinase-like ATPase (cluster 14), transcriptional regulator fused to uncharacterized DUF5753 (cluster 8), and AcrR family transcriptional regulators (cluster 6) are mostly linked to ATP-grasp in actinobacterial branch, whereas OmpR family response regulator (cluster 11) is specific for branch 5. Several protein families can be predicted to mediate the export of graspetides. These include ATPase subunit of ABC transporter (cluster 7) and TonB-dependent outer membrane receptor related proteins (cluster 146) most often found in Bacteroidetes. Enzymes, such as dehydrogenase (cluster 9), alpha/beta hydrolase (cluster 24) and peptidyl-prolyl cis–trans isomerase (cluster 145), can be predicted to modify graspetides. Cluster 145 and cluster 9 are mostly encoded in complex actinobacterial loci, whereas cluster 24 often is present in the loci corresponding to microviridin BGCs. The role of two other ATP-grasp-associated families, TPR repeat-containing proteins (cluster 5) and uncharacterized membrane proteins (cluster 40) remains unclear.

The straightforward “guilt-by-association” analysis described above has its limitations because there seem to be many non-orthologous gene displacements in the ATP-grasp linked BGCs [23]. As mentioned above, distinct peptidases are associated with ATP-grasps in branches 7 and 8. Additionally, distinct systems are implicated in the export of mature peptides, such as double glycine peptidase/ABC transporter, MFS system and others. Furthermore, diverse (predicted) modifying enzymes were often detected in ATP-grasp loci from the same branch. Clearly, this is only a part of the complexity of the graspetide biosynthetic gene clusters because potential functional connections with proteins encoded in trans were not addressed. Thus, each individual system should be analyzed on the case-by-case basis both computationally and experimentally, in order to establish both the chemical nature of the peptide and the proteins involved in export and regulation.

### Evolution of the ATP-grasp loci

**Fig. 7:**
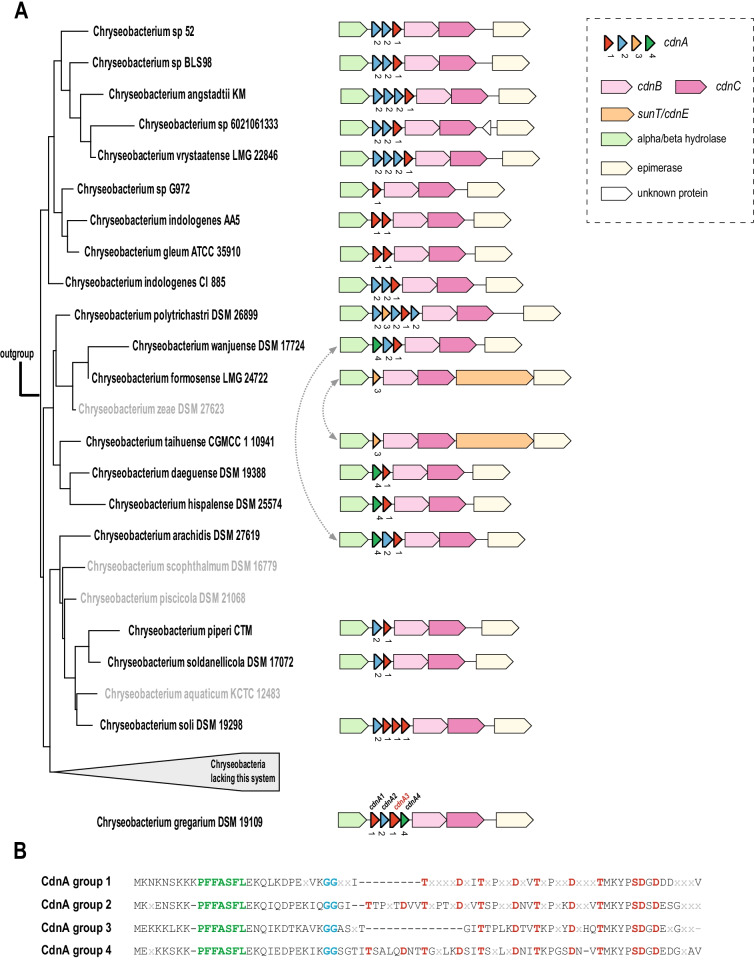
Evolution of chryseoviridin systems. **A** 16S rRNA subtree for Chryseobacteria species bearing chryseoviridin system is schematically shown on the left (complete tree is available in Additional file 14). Chryseoviridin loci are mapped to respective genomes. Genes are shown by block arrows, roughly to scale, color code is provided in the inset on the right upper corner. *Chryseobacterium gregarium* system in which CdnA3 peptides was experimentally characterized is shown separately (16S rRNA gene in this genome is incomplete and cannot be included in the tree reconstruction) [20]. Numbers below the precursor genes correspond to 4 clades in the respective tree (Additional file 5: Figure S5). Gray arrows indicate exchange of the genes by horizontal gene transfer (see discussion in the text). **B** Consensus sequences of four groups of chryseoviridin precursors. Consensus for multiple alignment of each cluster was determined as described in “Material and Methods” section. Conserved positions that can be involved in formation of ester or amide bonds are highlighted by red and correspond to the experimentally determined linkages [20], GG-motifs are highlighted by blue and characteristic microviridin group leader motif is highlighted by green

It is known that multiple graspetide precursors can be encoded in the same locus, and above we discussed the striking diversity of precursors associated with the ATP-grasp loci of branch 5. Thus, we were interested to trace the origins of multiple precursors in closely related bacteria. The recently characterized chryseoviridin system in *Chryseobacterium gregarium* is a good candidate for exploring the origins of the four distinct precursors encoded in this locus and tracing the evolution of this system in the closely related genomes. For this analysis we selected 47 species closely related to *C. gregarium* and reconstructed their phylogeny from 16S rRNA sequences (Additional file 14). We identified chryseoviridin loci in these genomes and built phylogenetic trees for all precursors, CdnB and CdnC ATP-grasp proteins and two flanking genes, alpha/beta superfamily hydrolase and epimerase (Fig. 7, Additional files 5, 6, 7: Figure S5, S6 and S7). Based on the resulting phylogenetic trees (Additional file 5: Figure S5), we assigned precursors to 4 clades and mapped this information to the respective loci on the 16S rRNA tree (Fig. 7A). Analysis of multiple alignments of the precursors showed that the N-terminal part of the core peptide region was much more variable than the C-terminal part which encompassed the conserved motif TxxxxDxxxTxKxPSDxD[DE] containing amino acids involved in the formation of 3 lactone or lactam linkages (Fig. 7B, Additional file 2: Figure S2E). The variable portion of the core includes amino acids that could form from one to three additional lactam linkages. Precursors of clades 3 and 4 were likely derived from clade 2, and most have one additional lactam linkage compared to clade 1 (Fig. 7B, Additional file 5: Figure S5 and Additional file 2: Figure S2E). However, we observed apparent independent losses of the segments of core peptides involved in the formation of one lactam linkage in clades 1, 3 and 4. Thus, it appears likely that both clade 1 and clade 2 precursors were ancestral. We also observed many tandem duplications of precursors, including recent duplication of clade 1 precursors in *Chryseobacterium soli* (Fig. 7B, Additional file 5: Figure S5). Horizontal gene transfer also played a role in the diversification of the chryseoviridin loci. Based on the phylogenies of all ATP-grasp associated genes, we inferred exchange (or acquisition from the same unknown source) of the entire locus between *Chryseobacterium formosense* and *Chryseobacterium taihuense* (Fig. 7A). Another example is the apparent exchange of precursors of clade 4 and clade 1 between *Chryseobacterium wanjuense* and *Chryseobacterium arachidis.* In this case, exchange of the precursors likely happened in situ because we did not observe grouping of these species in other trees, including clade 2 (Fig. 7A, Additional files 5, 6, 7: Figure S5, S6 and S7). Interestingly, in *C.gregarium* the precursor genes in the locus apparently were shuffled because at least clade 4 and clade 1 precursors are more similar to those of *Chryseobacterium hispalense*, where the order of these genes is the opposite and more similar to the order of these genes in other genomes (Fig. 7A). The CdnA3, the chryseoviridin is the most diverged precursor in clade 1, and possibly, was acquired from a distantly related bacterium.

Thus, evidence of duplications, losses of lactam linkages and exchange of the system components during the evolution of chryseoviridin system was obtained. Tandem duplications of precursor genes and intragene duplications or losses of small fragments (6–10 aa) of protein with potential to form one lactone bond appear to be the most frequent evolutionary events.

## Conclusions

In this work we compiled the most diverse, manually curated set of graspetides to date. This set consists of 174 families, including 115 new families with distinct patterns of amino acids implicated in macrocyclization and further modifications. This substantially expanded collection of graspetides can be employed as a training set for further improvement of automatic detection and classification of graspetides, in particular, using advanced machine learning methods. We also detected a leader region signature Phhx(1,2)h that could be also helpful for graspetide recognition. Notably, we have identified graspetide BGCs and likely precursors in two branches of CPR (candidate phyla radiation) bacteria. Establishing the role of these BGCs in relationships of parasitic CPR species with their hosts appears to be a promising area for further research. We also showed that graspetide BGCs of Bacteroidetes (branch 5) stand out in terms of the fast evolution of their precursors and in the apparent flexibility of their ATP-grasp enzymes with respect to the substrate structure. The ATP-grasp enzymes of branch 6 are specifically associated with complex BGCs and are predicted to be involved in biosynthetic pathways of antibiotics or other RiPPs that are possibly outside of graspetide class. Two distinct groups of ATP-grasps, branch 7 and 8, are identified as specifically associated with peptidases of different families and do not encode detectable precursors in the respective loci, suggesting the existence of novel precursors, possibly encoded in other genomic regions. Analysis of the proteins associated with graspetide biosynthetic loci led to the identification of a widespread group of predicted auxiliary extracellular enzymes (cluster 23 family proteins), as well as a putative precursor chaperone, the “LPL” family. Altogether, these results show that graspetides and molecular machinery, involved in their biosynthesis, are far more diverse than previously thought. Experimental study of these systems could provide insights into molecular mechanisms of inter-species conflicts and identify peptides with application potential.

## Methods

### Genomic DNA extraction, whole genome sequencing and initial analysis of MEBOG06 and MEBOG07

Genomic DNA from *Chryseobacteria* spp. strains MEBOG06 and MEBOG07 was extracted using the Wizard Genomic DNA Purification Kit (Promega, USA) following the manufacturer’s instructions with minor modifications. Briefly, 1 mL of an overnight culture in Luria Bertani broth was centrifuged for 2 min at 13,000×*g* and the supernatant discarded. The pellet was suspended in 480 μL 50 mM EDTA and120 μL of 0.1% w/v lysozyme was added. The mixture was incubated at 37 °C for 1 h. After centrifugation, the pellet was lysed with 600 μL lysis buffer at 80 °C for 5 min. An additional 3 μL RNase Solution was added and incubated at 37 °C for 45 min. After centrifugation, the supernatant was precipitated with 600 μL of isopropanol and washed with 70% EtOH. The air-dried DNA was rehydrated with TE buffer at 4 °C overnight.

The genomic DNA concentration was measured using a fluorescent Quant-iT PicoGreen dsDNA assay (Invitrogen). Genomic DNA samples (5–10 μg) from MEBOG06 and MEBOG07 were submitted to the NIH intramural sequencing center (NISC) for PacBio sequencing. Contigs from two separate CANU assemblies (40 × and 100 × coverage) were manually joined to form a single linear sequence. The joined linear sequence was corrected and polished using the PacBio reads and ArrowAssembly and assembled into a single contig of total genome size of ca. 5.3 Mb. Both genomes were submitted to Genbank under project ID PRJNA767328. MEBOG06 and MEBOG07 genome sequences were then analyzed for the presence of BGCs. Two complementary procedures were used in identifying BGCs. In the first procedure, gene prediction software GeneMark [41] was used to identify and translate the genes in the sequenced genomes, the resulting proteins were annotated by running RPS-BLAST against all annotated profiles in the CDD database (including CD, Pfam, COG, TIGR and other profiles, parameters: -e = 1 -t = @ -b = 10 -v = 10 -bt = 100 -rand -work = 2) [42] and the annotated genome was examined to find clusters of co-localizing genes encoding proteins involved in secondary metabolite biosynthesis. In the second procedure, the antiSMASH BGC-detecting software [4] was used on each genome (parameters: cf “on”, all extra features “on”, min number of genes in a cluster = 5, cf_prob_thres = 0.6, all types of clusters enabled, other parameters set as default). The two approaches yielded largely compatible results. Among the identified BGCs were microviridin BGCs found in both MEBOG6 and MEBOG7 genomes.

### Genome mining and loci analysis

PSI-BLAST [43] search (e-value cut-off was set to 1e−06 and the max target limit was set to 100,000 sequences, the rest of the parameters remained default) was performed using two queries, namely, the profiles of the two aligned MdnB sequences and two MdnC sequences from MEBOG6 and MEBOG7. The PSI-BLAST search was initiated from the alignment of homologous MdnB and MdnC sequences, which were aligned using MUSCLE v. 3 [44]. The search was run against a database of complete and draft genomes downloaded from Genbank at NCBI in March 2016, which contained 4,961 completely assembled genomes and 43,599 partial bacterial and archaeal genomes.

For each identified ATP-grasp gene ten genes upstream and downstream were collected and respective ORFs were annotated using PSI-BLAST [43] with E-value threshold = 0.01 run against position-specific scoring matrices (PSSMs) deposited in the CDD database [42]. Only hits to regularly updated databases, namely pfams, CDD, COGs, TIGRfams and NFfams were considered. Additionally, for uncharacterized proteins HHpred search with default parameters against PDB, Pfam and CDD profile databases was used [45].

In order to identify homologous proteins with low sequence similarity in the ATP-grasp neighborhoods, we further applied the following procedure. First, sequences were cluster ed using UCLUST [46], with the sequence similarity threshold of 0.9. Second, one representative sequence was chosen from each cluster, and the representative sequences were clustered again, with the similarity threshold of 0.5. Next, all sequences in each of the clusters obtained in the second step were aligned using MUSCLE [44], and a consensus sequence was derived for each alignment (including degenerate single-sequence alignments and consensus sequences for singleton clusters). Then, a PSI-BLAST [43] search using cluster alignments as queries was run against the database of consensus sequences followed by converting scores for a pair of clusters into distances using the formula *d_AB* = *d_BA* = *-ln(max(s_AB,s_BA)/min(s_AA,s_BB))*. Finally, a UPGMA (unweighted pair group method with arithmetic mean) tree was constructed from the respective distance matrix. This tree was dissected into subtrees with a depth cutoff of − exp(0.01)/2 = 2.3. All proteins from the same subtree were assigned to the same cluster of homologs.

For the initial set of candidates graspetide precursors, we selected the ORFs that met the following criteria: (1) encoded in the immediate vicinity of ATP-grasp genes (first and second neighbor in both directions); (2) the majority of sequences (> 50%) in the cluster were 150 or fewer amino acids in length. To identify homologs of precursors peptides with low sequence conservation, we used PSI-BLAST run for 3 iterations (or until convergence) with inclusion E-value = 1. The outputs were visually examined to exclude false positives. All sequence alignments were constructed using MUSCLE v.3 [44]. Minor corrections based on examination of pairwise alignment in the PSI-BLAST output were introduced in multiple alignments of selected precursor sequences.

Multiple alignment homogeneity was analyzed, and consensus sequences were derived as described previously [47]. Briefly, for each position, an amino acid with the maximum sum of BLOSUM62 scores against all amino acids in the corresponding alignment column was selected as the consensus amino acid. For positions with homogeneity values less than 0.5, the consensus amino acid was set to “x” (undefined).

### Phylogenetic analyses

Multiple alignments of protein sequences were filtered to retain the positions with less than 50% of gaps and homogeneity value greater than 0.1. Approximate maximum likelihood phylogenetic trees for the filtered alignments were built using FastTree (WAG evolutionary model, gamma distributed site rates) [48]. For the in-depth evolutionary analysis, we selected 47 completely sequenced genomes of the Chryseobacterium group, for which full size 16S rRNA sequences were available. Several Riemerella and Cloacibacterium 16 s rRNAs were selected as outgroup. An approximate maximum likelihood phylogenetic tree for 16S rRNA alignment was constructed using FastTree [48] with the GTR evolutionary model and 20 site rate categories.

### Comparison of ATP-grasp and RiPP precursor sequences with previously detected homologous proteins

The set of 2761 ATP-grasps sequences from this work was combined with the set of 136 sequences from Iyer et al. [23] and the set of 2036 sequences Lee et al. [24]. The sequences were clustered using BLASTCLUST with 80% amino acid identity and 80% length coverage. The sequences that fell into the same clusters with sequences from either or both of the previously reported sets were marked accordingly. Precursor sequences from Lee et al. [24] were compared with clusters of our candidate precursors using two approaches. First, these previously identified precursors were clustered using BLASTCLUST with 50% amino acid identity and 50% length coverage. Second, all proteins in the ATP-grasp neighborhoods were searched for perfect matches to the motifs of the 12 precursors groups identified by Lee et al. [24]. Several large proteins that were identified in this search but were encoded far from an ATP-grasp gene and/or have incompatible annotations were excluded from the set. If at least one protein from our cluster belonged to the same cluster with a precursor identified by Lee et al. [24] or contained at least one of the previously identified motifs, all proteins in the respective cluster were marked “known”. Also, we examined protein annotations obtained by the search against CDD database and marked as “known” the protein clusters that included at least one protein annotated as a precursor by any CDD profile.

## Supplementary Information

**Additional file 1**. **Figure S1. Identification of microviridin related BGC in two Bog Bacteria Genomes**. A. Organization of microviridin related loci. MEBOG06 and MEBOG07—two *Chryseobacterium* sp. genomes where the loci have been identified. Coordinates of the loci indicated on the right. B. *Chryseobacterium* MEBOG06 and MEBOG07 microviridin precursor peptides aligned with two of the closely related precursor peptides from known *Chryseobacterium *genomes. Class III precursor peptides as per classification in Ahmed et al, 2017. Green—leader region motif, blue—GG motif, red—core motif as per Ahmed et al. [15] and Lee et al. [24]. 600003570, 600003571, 600003572 are microviridin precursor peptides from MEBOG06; 700001629, 700001630 are microviridin precursor peptides from MEBOG07.

**Additional file 2**. **Figure S2. Multiple alignments of selected protein families. A. **Selected representatives taken from multiple alignment of precursors from cluster 2. Conserved residues in leader region are colored green, double glycine motif—blue, amino acids involved in ester and amide bonds formation—red; Underlined residues correspond to motifs of groups 8, 9 and 11 described in Lee et al. [24]. In addition, consensus sequences from *Salinispora* and cluster 199 aligned manually to show similarity within leader region. Abbreviations: gr8, gr9 and gr11—sequences with identified motifs of respective groups of core peptides delineated in Lee et al. [24]; “no”—sequences with no identified motifs delineated in Lee et al. [24]. **B. **Selected representatives taken from multiple alignment of precursors from cluster 13. Coloring is the same as in the Supplementary Figure 2A. Abbreviations: gr3, gr4, gr5 and gr6—sequences with identified motifs of respective groups of core peptides delineated in Lee et al. [24], respective motifs are underlined; “no”—sequences with no identified motifs delineated in Lee et al. [24]. The regions with a single core motif are shown by the red outline. **C. **Multiple alignment of LPL family of proteins. Alignments were colored using http://www.bioinformatics.org/sms2/color_align_cons.html server with default amino acid groups with 50% consensus. **D. **Multiple alignment of Cluster 23 and homologs. Alignments were colored using http://www.bioinformatics.org/sms2/color_align_cons.html server with default amino acid groups with 70% consensus. Residues within signal peptide region are colored cyan. Positions with conserved histidine, aspartate and asparagine marked by red letters H, D and R above the alignment. **E. **Multiple sequence alignment of chryseoviridin-like precursors. Alignments were colored using http://www.bioinformatics.org/sms2/color_align_cons.html server with default amino acid groups with 100% consensus. Amino acids shown experimentally to be involved in formation of lactam linkages are mapped on the CdnA3 sequence (Zhao et al., 2021).

**Additional file 3**. **Figure S3. Diversity of precursors associated with Branch 5 ATP-grasps. **The ATP_grasp subtree corresponding to branch 5 is shown. Cluster number of precursors identified in the respective ATP-grasp loci are indicated on the right.

**Additional file 4**. **Figure S4. Comparison of pheganomycin BGC locus and partly similar locus from Streptomyces viridosporus T7A. **Genes are shown by block arrows, roughly to scale. Homologous genes present in both loci connected by dashed lines and the percent of identical residues is indicated in red. A table with gene annotation for both loci is shown below.

**Additional file 5**. **Figure S5. Phylogenetic analysis of chryseoviridin precursors. **Approximate maximum likelihood phylogenetic tree was built using FastTree (WAG evolutionary model, gamma distributed site rates) (Price et al. [48]). Same program was used to calculated support values, which are indicated for each branch. Four distinct branches 1 to 4 are colored by orange, green, magenta and blue respectively. Precursors from *Chryseobacterium gregarium* DSM 19109 are underlined.

**Additional file 6**. **Figure S6. Phylogenetic analysis of ATP-grasps from chryseoviridin loci. **Approximate maximum likelihood phylogenetic tree was built using FastTree (WAG evolutionary model, gamma distributed site rates) (Price et al. [48]). Same program was used to calculated support values, which are indicated for each branch. Two branches corresponding to two ATP-grasp proteins CdnA and CdnB encoded in chryseoviridin-like loci are indicated respectively.

**Additional file 7**. **Figure S7. Phylogenetic analysis of flanking genes from chryseoviridin loci. **A. Epimerase. B. Alpha/beta hydrolase. Approximate maximum likelihood phylogenetic trees were built using FastTree (WAG evolutionary model, gamma distributed site rates) (Price et al. [48]). Same program was used to calculated support values, which are indicated for each branch.

**Additional file 8**. **Table S1**. ATP-grasp sequences in this study and features of respective loci.

**Additional file 9**. **Table S2**. Detailed information for ATP-grasp loci

**Additional file 10**. **Table S3**. Detailed information for graspetides identified in this work

**Additional file 11**. ATP-grasp sequences (fasta format)

**Additional file 12**. ATP-grasp tree (newick format)

**Additional file 13**. Final set of precursor sequences; Alignments of 174 distinct clusters of precursors (zip file).

**Additional file 14**. 16s rRNA tree (newick format)

## Data Availability

Not applicable.
