# Supplementary material for: Phylogenomic analysis of the diversity of graspetides and proteins involved in their biosynthesis
Source: Biol Direct. 2022 Mar 21;17:7. doi: 10.1186/s13062-022-00320-2 (PMC8939145; doi:10.1186/s13062-022-00320-2)
Supplement: Supplementary file 1 — Additional file 1. Figure S1. Identification of microviridin related BGC in two Bog Bacteria Genomes. A. Organization of microviridin related loci. MEBOG06 and MEBOG07—two Chryseobacterium sp. genomes where the loci have been identified. Coordinates of the loci indicated on the right. B. Chryseobacterium MEBOG06 and MEBOG07 microviridin precursor peptides aligned with two of the closely related precursor peptides from known Chryseobacterium genomes. Class III precursor peptides as per classification in Ahmed et al, 2017. Green—leader region motif, blue—GG motif, red—core motif as per Ahmed et al. [15] and Lee et al. [24]. 600003570, 600003571, 600003572 are microviridin precursor peptides from MEBOG06; 700001629, 700001630 are microviridin precursor peptides from MEBOG07. [file 13062_2022_320_MOESM1_ESM.pdf]

A

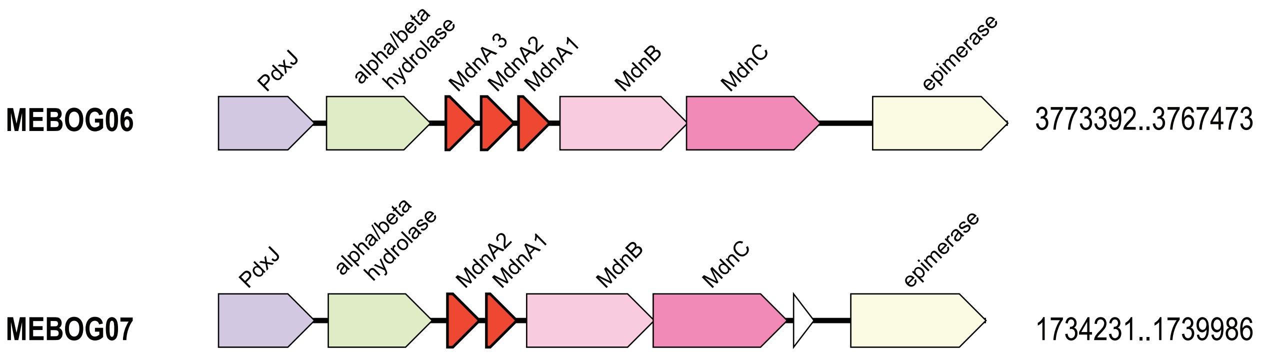

B

```
C. formosense   MEKKKLKK-PFFASFLENQIKDTKAVQGGASA----TGITTPLKDTVTKPAY-----------DQQQTMKYPSDGDESGV----
C. gregarium    MENRKSKK-PFFASFLEKQIEDPEKIKGGAVTSALVDNVTSVNKDNVTSSLL-DHVTKP---GGDNVTMKYPSDGDEDGNAV—

0600003570      MKNKDSKKKPFFATFLEKQIKDPETVKGGAGS----DMITIPERDFVTKPLA-DDVTSPQ--LDLMHTMKYPSDGDDDSMTMPL
0600003571      MENKNSKKKPFFATFLEKQIKDPETVKGGTSTSLLTDSVTSVIKDHITTAALEDSVTTP---NNDNVTLKYPSDGDDHVFEPQD
0600003572      MENKNSKKKPFFATFLEKQIKDPETVKGG-------GTITSVIKDHIITTTLEDTVVTTQINDHVNVTLKYPSDGDDDVLDV--

0700001629      MENKNSKKKPFFATFLEKQVKDPETVKGGAITSKLADQITSVIKDHITTSALEDSVTTP---DNDNVTMKYPSDGDDDVFEM--
0700001630      MKSNSSKKKPFFASFLEKQVKDPETVKGGT------DIISIPERDMITKPSV-DTVTSPK--DDMMHTMKYPSDGDDDTITIPL
```

Supplementary Figure 1
