# Supplementary material for: Phylogenomic analysis of the diversity of graspetides and proteins involved in their biosynthesis
Source: Biol Direct. 2022 Mar 21;17:7. doi: 10.1186/s13062-022-00320-2 (PMC8939145; doi:10.1186/s13062-022-00320-2)
Supplement: Supplementary file 2 — Additional file 2. Figure S2. Multiple alignments of selected protein families. A. Selected representatives taken from multiple alignment of precursors from cluster 2. Conserved residues in leader region are colored green, double glycine motif—blue, amino acids involved in ester and amide bonds formation—red; Underlined residues correspond to motifs of groups 8, 9 and 11 described in Lee et al. [24]. In addition, consensus sequences from Salinispora and cluster 199 aligned manually to show similarity within leader region. Abbreviations: gr8, gr9 and gr11—sequences with identified motifs of respective groups of core peptides delineated in Lee et al. [24]; “no”—sequences with no identified motifs delineated in Lee et al. [24]. B. Selected representatives taken from multiple alignment of precursors from cluster 13. Coloring is the same as in the Supplementary Figure 2A. Abbreviations: gr3, gr4, gr5 and gr6—sequences with identified motifs of respective groups of core peptides delineated in Lee et al. [24], respective motifs are underlined; “no”—sequences with no identified motifs delineated in Lee et al. [24]. The regions with a single core motif are shown by the red outline. C. Multiple alignment of LPL family of proteins. Alignments were colored using http://www.bioinformatics.org/sms2/color_align_cons.html server with default amino acid groups with 50% consensus. D. Multiple alignment of Cluster 23 and homologs. Alignments were colored using http://www.bioinformatics.org/sms2/color_align_cons.html server with default amino acid groups with 70% consensus. Residues within signal peptide region are colored cyan. Positions with conserved histidine, aspartate and asparagine marked by red letters H, D and R above the alignment. E. Multiple sequence alignment of chryseoviridin-like precursors. Alignments were colored using http://www.bioinformatics.org/sms2/color_align_cons.html server with default amino acid groups with 100% consensus. Amino acids shown experimentally to [file 13062_2022_320_MOESM2_ESM.pdf]

Supplementary Figure 2. Multiple alignments of selected protein families

A.

Cluster2

```
CONSENSUS          PxxLxxx-xPxx--xxx-xxxx-xxxYDPxxQxxxxxD--------Gxxxxxxx-----xxxxxxxxx--xx----xxxDxx-----------xxxDx-xxD--
1115239570_gr9     PFGYLFA--NWEPGTPA-ETETELGYYDADQQVWVTPD--------GAITAGV------YTKTRTSGS--------NCGD-------------CVTDD-ACA--
1302152154_gr9     PFAYAFA--TWTEDAEE-AQDD-TGHYNEETQVWEIPG--------GGYTMGV------YTKR-TGCS--------CHPD-------------YVTDD-ACF--
1084936075_gr8     PFGARAERTPY---TVT-LDAG-SFVYDPARQVNVLTD--------GSGALWSR-----STSLAGSCT--TN--WDSKNDDT-----------SDPY--LMR--
1504406364_gr8     PFAMRYAARQTS----E-PADTTSVTYDQGLQIAVTSD--------GNPWA--------HTALGETSS--------TNSDSR-----------NDEGT-DFY--
1605672014_gr8     PWGLRRL-APYP--AVD-APQYARVELDAGTQTARYLDAA------GAVVMAPG-----HGTSSGTNPPTGTT---GQGD----RNSDAPDSDTGND--TDQ--
1301744864_gr11    PFALQYA-RPQ-----TGEPAA-PYAFDQAEQVNVLAG--------GGYAAED------TALLARLGS--AS----TAGS--------------KTH--WDD--
1806787427_gr11    PFALNYA-RPAR----E-LVVSTPYVYDSGLQLNVLAD--------GRVAACD------HALLREVGT--TS----TAGS--------------KTH--FDD--
1301678086_gr11    PFAWNYA-QPAD----P-APALVSYSYDATLQLNVLSD--------GRPAISD------RALMAAAGT--TS----KAGS--------------ATH--FDD--
1101511595_no      PWGLARM-RPYPA-AAV-LPAA-RPVLDPHTQVPAWVGPD------GAPLTAQAKHKRSETSQETST---TS--LDGTPDQG-----------SDQNG-DSD--
1301420005_no      PFGLTLS-TQVGDEVPV-VDLA-DARLDPVTQTVVGVD--------GVALGKP------QTTQKVTTN--KT----SYDHQN--------FNDSTPDT-YQD--
1504264967_no      PWALRLVTDRLP--VGP-PPYA-TVTLDPVTQTARYADAA------GRPVEMGKH----GTSRTTSTA--VS----GGGDGN--GPQSQTQDDSTTDY-ESD--

Salinispora        PVSFGFGQARPMP---DNPLDLTGVVYDPTLQVA-TVDGV-PYAELGGPLA--------ATVRQTRED--------GQL--------------------WDDKD

Cluster199         PL-LP---------GTG-GATAPQHRYDPARQLV--VDDT------GAPVxxDP----xSTIMxTGHx--------GPH-----------ERADRPDG-MDSWW
```

B.

Cluster 13

```
CONSENSUS          PxIxxYxxxx--xxxxxxxxxxxxxxxxxxxxxxxxxxPxIxx---xxxxxTxTxx---xxDDDxxxxxxx----xTxTxx-xExDDxx----------------------------
1003947440_gr3     PYIANYIKEKNFNKTKRFAYN--------------------------KKEQVSFIDE---RKVIKEYF-----SSSTRLTKS-VENEDEDEFKC-------------------------
1044887267_gr3     PYTMLYSESFEINASTISNKT--------------------------NSTVKTFTTE---PNDED-YL-----NTWTTHTRV-IENQDEDY------SL-APSNCERKYEISN----
1102345754_gr4&3   PYIGQYVEEVELLDGPALYYD--SVTQ--------------------MSYTDREMTR---RAIHNIEK------DTTVITDT-IEQSDPDE------FSLSQTTMKTATIENGDPDE
Thuringinin_gr4&3  PYIAKYVEEQTLQNSTNLVYD--DITQ--------------------ISFINKEKNV---KKINLGP-------DTTIVTET-IENADPDEPYIA--KYVEEQTLQNSTNLVYDD--
1011944976_gr4&3   PYIMNYSETITMKQSVAIQNT--NILD--------------------VTGTRTFTIE---TDDHDEIL-----GLATIITER-IENSDRDE------IS-AYSTFQTNIVENSDKDE
1904508545_gr5     PLIFDYMREQ--TNVEFPELTYDRKSGLNFI-NGTKFVDLCNSKT------------------------------VLQTKTFTQRESDDEEKPYTSSISKMFLVTKTDTIREVDDD--
1080071630_gr5     PFLARFATER--TEGENIPGWYSYEQDLWVVEEDSKEIPIISKYT-LTQTVTKTKVR-QESDDDSS-VSML--ELTTKTEAQLERDDSDLSMNH---LLELMTKTDTIQERDDDN-
1305270918_gr6     SFILGFAEKPKINNLDYSIIEYSKKQNLSVL--KNTEIPAITYVNMGTETFTKTTNEPTDSDNDYRFRLKRLLDTTSETFTTTEPSDSDHNYSSLKLLMDTQTITE-SQEPTDSDK
1506242654_gr6     YLLFEYGESPT-YMTDIPPVKYDYDKNLNVL--ESNNTPAISV-LDNCVTETGTKVHNESADSDNNFPLNF--GTETFTLVDTEQSDSDEDSILLSSSLITMTTRVEMESTDSD-

CONSENSUS          ---------------------------TxxxExDDDxxxxxxxx-xTxx------xDDDxxxx---------------------------
1003947440_gr3     --------------------EETTALTENVENDDEDEFKCL--ESTLYSETIENDDEDEVSLFFG-----TKKTAKIETSDPDELNI
1044887267_gr3     ---------------EKQS-AMDTFFTESIENNDDDFIVC----RTFVTNVVESGDDDFYEN-------------------------
1102345754_gr4&3   -----LSL------------SETSMETRTIENGDHDEIFLF--GTTKQTFTIEDSDPDEYMMTD-----TTRITKSIEGSDPDEFYS
Thuringinin_gr4&3  -ITQISFINKEKNVKKINLGPDTTIVTETIENADPDEYFLD-PDTTIHTFTVENNDTYFLDDEHYCGPETTRITKTLENIDPDEYYS
1011944976_gr4&3   ----------------ISC--AGTMVTAVIENSDTDEYLLF---STMETRELEAADSEEISYLMSDS---TIMTKVFEATDNDE---
1904508545_gr5     ----------------MNFNNLTYLLTKTEENKEQDE---------------------------------------------------
1080071630_gr5     ----------------WCAGNILELSIKTFVSVERDEDFSMHTYDHQREGLI------------------------------------
1305270918_gr6     ----------------------------------------------------------------------------------------
1506242654_gr6     ----------------------------------------------------------------------------------------
```

C.

LPL family

```
Consensus 50%
1028378093 --------MEKKEFVSLFKEESYKLLPINYHLIRNVYYTKEEVKRFNLH----YSAH-------SNFHQT------TIYDIFY----N--------------------- 59
1506709131 --------MEKKEFVSLFKEESYKLLPINYHLIRNVYYTKEEVKRFNLH----YSAH-------SNFHQT-TI---YDIFYNY----KPII--IL-------------- 66
1604649742 --------MEKHEFISLFKEESYKLLPINYHLIRNVYYTKEEVKRFNLH----YTAH-------SSFHQT-TM---YDIFYNF----KPII--IL-------------- 66
1502594610 -------MNIQDNKEIIFSAETIKKLPLGYRFL--TKMSTDHVPELRIGA---KYSQ-------SKRHGA-DA---YLTFDIE----KPIS--NIYLDIPIINDTQQF- 79
1107402590 -------MKNKLKKKTIFTAKELKNLPVTYRMM--DEKQTEEVGELYTQA---VFTT-------SARHFS-NQ---FKMYISP----KPIS--NFIITKND-------- 72
1033505825 -----MADKKRHNFEQLIYNKDLKKVPLVFRFL--EVKKTDEIGELYMHV---KFSS-------SIQLQT-KS---LNNYLDS----VPMS--NILIEDSDCNERVK-- 80
1080909573 -----MVNKKRQKFEHLVEEKELKNLPLAFRFL--IKRPTNIIGEMYSQV---RFSS-------SSKLQT-KS---LNNYLDF----TPIS--SIFISKEK-------- 74
1500267439 -----MAYRKRQKYEDLIAEKDLKNLPLAFRFL--IRRETNTIGEMYSYV---RFST-------SKKLGT-CS---LNNYTDL----TPIS--GIFISEEK-------- 74
1301770241 ----------MDINKKELTDEQIENLPLFFKTI--YYNKMVSIDAENQL----MKYN-------YNIHAV-EM-------RHY----NPNR--RICLPYDFSQFANK-- 70
1505711076 ----MEVKEPKEEKTYYYPTELRTELPVNFFLS--KIIAQNASAKIPYLK---EKVY-------CKMHNT-KC---YPVGTFQ----KPIS--NIL------------- 70
1019511433 ---MKKKETVFPRLEKIFGVETAQTLPPYIQLA--QRMPSNAEENHEIR----KNET-------SDLHQT-DF---YDVYPLN----KPVS--KIFDPKVLNNDQS--- 80
1032991484 -------------------------MSFYLSQM----HENTKVVQYPK-----AAAK-------STFYNA-SD---IACFPFY----KNPS--LIIDLNDQQQ------ 52
1305268634 -----MKNIIKQEIKTLEDIVSLQELPIYFKHL--TEIAFSEANTQNV-----IVTP-------SNKHQT-DK---HKLSFFF----KPIV--KIHP------------ 68
1405872909 ---------MFEIKEYKIAKESSKTFPLGYNFI---KYNHLSVPKSEEY----YVIK-------IKRYNS-E----MGPYRST----KPIS--KIFDFTPIQKELKNE- 74
1402605514 --------MNQKEMGEIPELKGVNGLPVTFKLL--TKVAVNETPKKN------LFFY-------VAAYQ--GM---ALAEAFE----KPYS--KIINWQE--------- 66
1502591667 ---------MNIKEYLNIAKEKFAGLPSSFKFLFLVKFDKAVSMKMKSKR---YGVS-------VDAYQS-ET---YQPTGHY----KNIS--KIVDYKIIQEKNG--- 77
1064363958 ----------MIDKRDIFFKKQLDNLPFFLRN---HKIKELQVSKNSSEVTVSYNDQ-------FPVYHS-ET---VECNLSL----KPIS--KLLTFDEQKEF----- 74
1306133393 ------MNHEEKISDYIKKNKLSKEVPVILHKI--ESVNLNDKVVI-------KTFK-------NSVFKT-NK---MVCFSFY----KNIF----------------- 61
1701924764 -------MTKKQKTIPELAEIAEKHLGVSLPFY--LKKTKDINKQISIF----RYDT-------STFYKS-NF---VVSKPFY----KMIH--MPFNNEYYENNPDE-- 77
1017537696 ---------MIKLKKLSISEPQLENLPLFYNYL--IYNELLPS----------------------DPFFRT-NYSEKYSSIIFYSSGDKPIS--KILDTTQLKK------ 68
1032986777 ---------MQKENKVRISDKDKKSLPVFCYSI---YFNEMISREIEN-----KLFT-------YGQNSE-SI---YGRYSAT----RPIS--KIIKMENLK------- 68
1202920038 -------MKKKKLKKLLLEKTIEGELPVFFTKP--IFLNEEDDSFIMNQ----KFLS-------VKDYQTNKYN--IPDIKII----RPIC--KTFKL----------- 70
1306177980 ------------MKFIEFKKNINENPQIFSLPM---FYLEEQPISISKR----KVLF-------SNFYNG-N----YILDPFY----KNIH--RLI------------- 59
1505713340 ------MKKHFKELGINKGKASKIPLTLTLATL--KPKDKNKIRI---------IFE-------TQAHQS-TR---VPLKPFY----KKIS--RFI------------- 62
1015114672 ------MQNNHETLLKNKLKKINSSFILLDLGK--LKVKKNILDNNENL----QIKG-------NNKIKN-SI---TFSRKLY----KPIS--KL-------------- 66
1207124865 ------------------MESKKPINI-FNGL--KLKVERETQPLPKN-----NLIT-------IQAYQN-SK---NISKPFV----KPIS--KII------------- 53
1505713717 ---------MNNINKKLFQEEQEHSLPLIAHFL--NTKELTTIKVEKQL----KYVA-------IDFYQS-QA---IKTYPYY----KPIS--KIITSASYRK------ 71
1202921271 ----------------MKARVNIKEEIKSGKAPIHLYSTTKSNRLSVLV---KARI-------SKSLKT-DW---LTIDPFY----KSVI----------------- 56
1122343331 --MMKKEQEKIKEELKNWIEENMPQLSPNAGTW--HIEVYENTADAEPQ----EPIK-------LGRYDA-EV---PSSNPFY----KPYS--KMFVLPL--------- 75
1604611437 -MKKNCKKEAIYNEIKQWITDNKDQLPLNVNTLGVRVWETDKTNQH-------FAIH-------LSKYAS-EP---SFVNPLY----KPYS--KMFDGSK--------- 75
```

```
1604690910 ---MKEEQEKIKEKVQNWMAENKSQLSLNSDTW--NIDVFENKKNTEFQ----KSLK-------LGKYNA-EL---TESDPFY----KPYS--KMHIL----------- 72
1116761953 -------------------MMKKNKLSIYMQEH--DKIKKVIDSNTIHK----NKIK-------VKKLNS-NN---YQNCNFY----KPIS--KILSFESEK------- 60
1300684408 --------------------MKKNKLSIYMQEH--DKIKKVIDSNTIHK----NKIK-------VKKLNS-NN---YQNCNFY----KPIS--KILSFESEK------- 59
1806090729 --------MIDDSEKYKDLISIPEHPSISYDLH--KISISHQSKKNNSK----RFIY-------MKKYRG-EG----SILDFY----KPNS--NILSIKEND------- 70
1019258132 -----------------MEDNIKIKEPLLLKFL--TPVPDVTKITEPVRG--------------NTYYNA-GA---FDVIPFY----KAIS--LLVTHESNLSINNIEV 66
1080819527 ------------------MNKSDGFAPLAFHVL--DKNDNATKKRFRN-------AE-------SKIFQT-NN---HPFVSFY----KPIS--ILGKKNGK-------- 57
1004548170 ---------MKKSKDLIEALNACEDAPLTLKKM--KQTKNTLPNYSTFK----KLFE-------VEKYDS-KA---VRLVQSY----KPIS--KL-------------- 63
1004550496 ----------MKKSISQIIKDDKSS-PLTLKKM--KKSKNATPKMQYK-----TLFQ-------VSLYES-SQ---VRVVNFY----KPIS--KL-------------- 60
1701928595 -----------------MQLIKIEQLPLTFHKA--TITETATIKKAKKE----HFIL-------IKKIGS-DK---ISTEKFY----KPIS--KIFSFDED-------- 61
1701928322 -----------------MKNDLLKNVPLTIQNA--TVTQKILQKHTT------KLIS-------IKKYQS-TQ---NTSDIFY----RPIS--KL-------------- 53
1081398938 ---------------MKKNIKQGVENLPLLYQL--SEVEEIGGGEPIQP----VVSRRPNRVIKMEVYES-VK---TPTGGAP----KPVT--KSFNFFK--------- 69
1702143276 ---------------MKPLIKNKKYLPIIYQNL--EERPTGTSGKT-------VTIK-------VKSINA-T----LKTGQFH----RPIS--KIISFNE--------- 58
1105847656 ------------MPDHLKNIIKRNKGSYILRDLDPLFSPNEKLPDYRN-----LNIK-------SSFRNGSGI---YLSKKFY----KRIS--KF-------------- 62
1604693137 ------------------MKTNKYKKSILLKNL--KPVSGGKA----------VLID-------IDRINS-SE---TFSFLFY----KPISKFEILDDEKNK------- 57
1500393663 -----------------MNNKEKIEAPLFLRSI--EERKISQHKEESSNE---KYCY-------SQKYQGYCI---FRKSNRW----KSIA--ISINNDKD-------- 63
1085296070 --------MKNSLLKEMKLEKKKEFLPIIFSHT--NPVDETKIKRNREY----RIIK-------TRFHQS-KI---YCLRTYK----DGIN--ISTE------------ 66
1095771966 ---------MSTYKLIKEMKLNKEKLPATFSLI--ERVDYINTTVLKIY---------------SPYYN--GY---FNMKVFH----KKIH--RLL------------- 59
1102742539 MLFKKNEYMKKTLLKEMGYEKKKELLPIIFSHT--MPMDIKKIKRERPF----RMVE-------TSIHQS-EI---YCLRIYK----DGIN--ISTE------------ 74
1604645044 ----------MKKSLIQELKHNKELFPVVFSNI--FPFPDTKKARRNNLS---RQVT-------TSIHQS-EI---HCLRIYK----DGIN--LYTEQ----------- 66
1085906968 ------------------MNRRKNKFNLIYFLMDVEFKQEIESFNDDQQNFS-DIVF-------SKKFNT-SK---LKTKVYD----KVST--PFINTNLFHEEL---- 69
1013201691 ---------------------MKNRINLIYFTLTPKLTEYLKKISLNAPSNKKYNIL-------SKRYNT-NN---LKVKIYD----KVST--PFIDIKLFDDEE---- 67
1403764038 ---------------------MKNRINLIYFTLTPKLTEYLKKISLNAPSNKKYNIL-------SKRYNT-NN---LKVKLYD----KVST--PFIDIKLFDDDE---- 67
1008400074 ------------------MNLMKNRINLIYFTLTPKLTEYLKKISLNAPSNKKYNIL-------SKRYNT-NN---LKVKLYD----KVST--PFIDIKLFDDDE---- 70
1012269384 ------------------MNPMKNRINLIYFTLTPKLTEHLKNISLNVPNSKKHQIF-------SKRYNT-NS---LKVKLYD----KVST--PFIDIKLFDDDE---- 70
1403778845 ---------------------MKNRINLIYFTLTPKLTEHLKKISLNVPNSKKYHIF-------SKRYNT-NS---LKVKLYD----KVST--PFIDIKLFDDDE---- 67
1074098712 ------------------MNLMKNRINLIYFTLTPKLTEHLKKISLNIPNSKKYHIF-------SKRYNT-NS---LKVKLYD----KVST--PFIDIKLFDDEE---- 70
1024086609 ------------------MNLMKNRINLIYFTLTPKLTEHLKKISLNVPNSKKYHIF-------SKRYNT-NS---LKVKLYD----KVST--PFIDIKLFDDDE---- 70
1114742664 ------------------MNLMKNRINLIYFTLTPKLTEHLKKISLNIPNSKKYHIF-------SKRYNT-NS---LKVKLYD----KVST--PFIDIKLFDDDE---- 70
1038225731 -----------MKRFLERFNSEKDFLSLSLNAL--LAEEIDENKEVSVMKVEYRSLS-------CSYYNN-KD---HVSFSYF----KDIIIPDID------------- 69
1305268641 --------MTKQENFKKLLKNNRENISLSFNNI--TIKQSISAIGG-------KFVA-------IDFYDT-NR---FHVSTNV----KSVT--RFL------------- 62
1015112256 -------MEKKTESFEQFFKENRNKLPYYFKDL--KATK-LVVDDNNITIN--KIFN-------SNFFNS-KY---CIVERFY----KDIV--RL-------------- 66
1086846106 -----------MEEKIFKSLKEINYLPFSARFW--EKLPQKRSLHQNKG---------------SVKFQT-ND---FLNEIFY----KSIT--RKV------------- 58
1022634858 ------------------MKINDERLPFYFKLM--TKTEILDVVEDDT------LGK-------STKHMT-DA---YYIHDSY----KPVADPQLLINQKK-------- 60
1017538469 -----MEAKKTKEEIVEHLERTKGILPLILHDV--NV---TVIEKNNKVTG--KLIT-------VPSYKT-SG---ISISNYL----KHVT--RFPHE----------- 69
1050776254 -----------MKKKFISNLKDKKCLPLSIYLL--E-ADGYSSKNFKENMI--KCEK-------AEKINA-KG---YELLSFY----KVIS--KF-------------- 62
1008126603 --------MIDKEEIKQVLKRDEEILPISFNQT--IVRDKVDFSKKNL-----KSVK-------SIYYKD-GD---YQIFPYY----KQIT--QF-------------- 63
1089032244 ---------MTKEEIKKYLKDNKELFPLSFFEL---KYRERSIENISKY----KIAV-------SEKYFT-DN---FPIYKFY----KPIT--RF-------------- 62
1049905012 ---------MEKDLNEFFENKLENLPLYMRYIQFKEKNDNRI-----------EVIH-------VEKHNT-TK---YKVRPFY----RPLQPLKL-------------- 60
1008125305 ------------MEKNKILEEFKQCLPLSLQNI--ELKENLSAVTG-------KVYP-------VEKYNT-KV---YPFLQFY----KHIT--RII------------- 58
1085296302 ------------MKKVTTKLKNKKLPLPYWNIQ-KELSDKKLGDIPAM----MYSN-------SDLYQT-RG---YVVEGSR----IVIS--KIIS------------ 62
1604691190 ------------MKKITTKLKNKKLPLPYWNIQ-KELSDKKLGDIPAM----MYSN-------SDLYQT-RG---YIVEGSR----IVIS--KIIS------------ 62
1004551140 --------------------MKKDNLPLIFNAL--KTVKSNSKKEG-------FFVT-------CSQLQT-KR---LELNAYY----KKSS--NFLKK----------- 52
1102458186 ---------------MKNIIQEKNKLPLTFSYL--EIIKRKKTIRKSVQIA--YYTK-------SKKYQT-DF---LLIEEPE----KSLV--RLL------------- 60
1028377461 ----------------MKKNKEQHKLPLIFKYL--EFLPKTAHMSYKD-----NIIV-------CGKYQT-NF---YGKNISV----KPLT--RML------------- 56
1079182110 ---------------MNKKFKEKNKLPLTLKYM--ECFRVNNPKIEDIYKN--DYIV-------CKEYQT-DF---LMKAKPV----KSLT--RLL------------- 60
1506937980 ---------------MKKKFKERNKLPLTLKYI--ENFRGASAEIQDSHKI--NYVT-------TVEYQT-NF---LMRIKPT----KPLV--RLV------------- 60
1604652443 ---------------MKKKYKERNKLPLTLKYI--ENFRDASAEIQDSHKI--NYVI-------AIQYQT-DY---LMTMKPT----KPLV--RLL------------- 60
1016680128 -------------MKRDFTTKEKHKLPLTFKYI--DVVNSKSREKRYFS----ITIS-------CEKYHT-DF---YAKLNPY----KFLT--RLV------------- 60
1604617185 -------------MKRDFTNKEKHKLPLTFKYI--DVVKSKSREKKHFS----VTIP-------CEKYYT-DS---YAKLNPY----KFLT--RLL------------- 60
1011496328 -------------MKKNFTPKEKHPLPLIYNYM--EMIKNKEREKSIAN----TTIA-------SEKYQT-DF---YTPSKPY----KFLV--RFL------------- 60
1095770552 ---------------MKKISKEKNILPLIFKYI--EFSKNKLPIKDKSKLT--FFVE-------CEKYQT-EF---LVRSKPY----KSIT--RLL------------- 60
1604643112 ---------------MKIKVKEKNKLPLTFKHI--ESFNDQKLKKNNDVI---YVLD-------CEKYQT-NF---YIRERPI----KSLT--RLL------------- 59
1604648334 ---------------MKIKVKEKNKLPLTFKHI--ESFNDQKLKKPNDII---YVLD-------CEKYQT-NF---YVRERPM----KSLN--RLF------------- 59
1011495981 --------------MKKLFKEEKNKLPLTFKFI--EKLGTKKSNDKNSINI--HYVE-------CETYQT-DY---LMRERPI----KALT--RLL------------- 61
1085297867 ---------------MKKLYKEKNKLPLTFKYI--EIFKTKNVSKIHSSMV--YSVP-------SEKYQT-EY---HVREKPL----KQLT--RLI------------- 60
1095767320 ---------------MKKLYNEKNKLPLTFKYI--EIFKTKNVSKIHSSMV--YFVP-------SEKYQT-EY---HVREKPL----KQLT--KLL------------- 60
1095776607 ---------------MKKLYNEKNKLPLTFKYI--EIFKTKNVGKIHSNML--YFVQ-------SESYQT-EH---HVREKPL----KPLI--RIL------------- 60
1604692379 --------------MKKLFKKEKNKLPLTFKYI--EELRTKKNNKNNSTG---NHVP-------CEKFQT-DY---LLREMPI----KSLT--RLL------------- 60
1604610696 ---------------MENQFKEKHSLPLTFNYI--ELIKKTKSGKENKNII--YFIT-------CKKYQT-DF---YVREKPF----KPLT--RML------------- 60
1079182107 ---------------MKNTFGEKHKLPLTFKNI--TKFGNVNNVKRNSNTA--YFLE-------SKKYRS-DF---YIREKPF----KSLT--RLL------------- 60
1079179758 ---------------MKKNFGEKYKLPLSFKQM--EYFKHKNSNKIDSNTI--CVLK-------SEKYQS-DF---YVREKPV----KSLF--RLL------------- 60
1079170133 ---------------MKDNFGEKHKLPLSFKYM--DFFDNTDSKMITSNTK--CILR-------CKKYKT-GF---YVREKPV----KPLI--RLL------------- 60
1604802081 ---------------MKENFGEKHKLPLSFKHM--DFFDNTGSKGITSNTK--CILR-------CKKYQT-SF---YVREKPI----KPLI--RLL------------- 60
```

D.

Cluster23 and homologs

```
1064363960 MKYIYIIGFVLIISCSKQNNNQIKSHSINLDSLITSLNTQEKKEKFLVNLFQQDQNVRNSDRENEI-----LKRNNYNVDSSEYQDYRRRIKE-----VD 90
1091210526 -------------------------------------MKIKIVNFFLSLLFKVDQKVRYRGKYGVL-----------------------PVKI-----TD 35
1091100979 ----------------------------------------------------------------ML-----------------------PVKI-----TD 8
1091209206 ----------------------------------------------------------------ML-----------------------PVKI-----TD 8
1091136844 ----------------------------------------------------------------ML-----------------------PVKI-----TD 8
1091234973 ----------------------------------------------------------------ML-----------------------PVKI-----TD 8
1091111073 ----------------------------------------------------------------ML-----------------------PVKI-----TD 8
1090995448 ----------------------------------------------------------------ML-----------------------PVKI-----TD 8
1091125220 ----------------------------------------------------------------ML-----------------------PVKI-----TD 8
1091055562 ----------------------------------------------------------------ML-----------------------PVKI-----TD 8
1091167037 -------------------------------------MKIKIVNFFLSILFKVDQKVRYQGKYGVL-----------------------PVKI-----TD 35
1017537694 -----------MKMLLFITIITISMNMESQNKIKITQLDYKELQTQLEAIRIEDQTLRQLLPEATA---------KFGADSDELKHIWKLIHK-----QD 75
1507123407 MYTKFHYILLIFALCFFGC------------KKEEKTDSYFKVRKTLEKVLEDDQKYRQQATTDWW----------------------KEQGK-----LD 61
1506709129 MYTKFHYILLIFALCFFGC------------KKEEKTDSYFKVRKTLEKVLEDDQKYRQQATTDWW----------------------KEQGK-----LD 61
1028378095 MYTKFHYILLIFALCFFGC------------KKEEKTDSYFKVRKTLEKVLEDDQKYRQQATTDWW----------------------KEQGK-----LD 61
1604649744 MITKLKYLLLVFILSFFGC---------------KKTENYSEVRKTLEQVLVDDQKFRQPMDFIKQ-------------------------AP-----LD 55
1604693139 --------------MSKRIIAVSMLLLITSCKSTLSPTEKRNLTNEIDAMGTIDQVAAYIPQGKYA--------------NYSRQRWSSFKDS-----VM 67
1034294064 --MRRFGLLGWVLVLSSSPVWAVDLSQAMHACPGAAAFIEAQVAQQRAALSVGPSVVSDNVRRERL-LALQDEDQRLYERLASGSIDVTALMP-----VK 92
1701082198 ----------------------------MDACPGAAKFIQAQISRQLAAGPPKAPAVTDEAKRHRI-LEHEEEDQRLYEQLASGSVNPTAFKE-----LQ 66
1701091927 ----------------------------MDACPGAAKFIQAQISRQLAAGPPKAPAVTDEAKRHRI-LEHEEEDQRLYEQLASGSVNPTAFKE-----LQ 66
1701098556 ----------------------------MDACPGAAKFIQAQISRQLAAGPPKAPAVTDEAKRHRI-LEHEEEDQRLYEQLASGSVNPTAFKE-----LQ 66
1701107844 ----------------------------MDACPGAAKFIQAQISRQLAAGPPKAPAVTDEAKRHRI-LEHEEEDQRLYEQLASGSVNPTAFKE-----LQ 66
1701085478 ----------------------------MDACPGAAKFIQAQISRQLAAGPPKAPAVTDEAKRHRI-LEHEEEDQRLYEQLASGSVNPTAFKE-----LQ 66
1701063269 ----------------------------MDACPGAAKFIQAQISRQLAAGPPKAPAVTDEAKRHRI-LEHEEEDQRLYEQLASGSVNPTAFKE-----LQ 66
1701088627 ----------------------------MDACPGAAKFIQAQISRQLAAGPPKAPAVTDEAKRHRI-LEHEEEDQRLYEQLASGSVNPTAFKE-----LQ 66
1701069525 ----------------------------MDACPGAAKFIQAQISRQLAAGPPKAPAVTDEAKRHRI-LEHEEEDQRLYEQLASGSVNPTAFKE-----LQ 66
```

```
1003372624 --MRLVSALGLASLLFIDSCGAVDLSQAMDACPGAAKFIQAQISRQLAAGPPKAPAVTDEAKRHRI-LEHEEEDQRLYEQLASGSVNPTAFKE-----LQ 92
1200090063 ----------------------------MDACPGAAKFIQAQISRQLAAAPPKAPVVTDEAKRHRL-LEHEEEDQRLYEQLASGGVNPTTLKE-----LQ 66
1000489224 --MRLFSALVLASLLFIDSCGAVDLSQAMDACPGAAKFIQAQISRQLAAAPPKAPVVTDEAKRHRL-LEHEEEDQRLYEQLASGGVNPTTLKE-----LQ 92
1000112493 --MRLFSALVLASLLFIDSCGAVDLSQAMDACPGAAKFIQAQISRQLAAAPPKAPVVTDEAKRHRL-LEHEEEDQRLYEQLASGGVNPTTLKE-----LQ 92
1101210601 --MRFFSALVLASLLFIDSCGAVDLSQAMDACPGAAKFIQAQISRQLAAAPPKVPVVTDEAKRHRL-LEHEEEDQRLYEQLASGSVNPTTLKE-----LQ 92
1091018262 --------------------------------------MDNNIATLLSNLIAVDQGLRKNKIFSET-----------------------LMQK-----FD 34
1091256281 --------------------------------------MDNNIATLLSNLIAVDQGLRKNKIFSET-----------------------LMQK-----FD 34
1091176042 --------------------------------------MDNNIATLLSNLIAVDQGLRKNKIFSET-----------------------LMQK-----FD 34
1033412857 --MSKRLVLLAALLWL-------------AQVLPAQARPECAAVPQQIRTMIDRDQALRLRVNQLPP----------------GDTSLDAELLR-----TD 65
1303959073 -------------------------------------------MSQILSAQGYEKQFLANAWSVQL-------------------------CA-----ID 27
1300606538 --MMMRLITTATLLAIAGGSFARPQQEQPMAHSVEVEGVNADYRQQLLQLAQADQQIRADVLKDYT-------PQQLQNDPGSARALALKIHA-----SQ 86
1049540699 --------MLGMLLWQLATMGHAVAGTGHSSQPD-HQSMNAAYREQLLALADADQKIRQQIRGAFT-------VQQLQANQGAAKEMATRLVA-----SQ 79
1081417263 ----MKTVVMALLLLWQVALPGQGVATDSKALQSGHQSMNASYREQLLALADADQKIREQIRGAVT-------PQQLQANQGAAKEVAMRLVA-----SQ 84
1203949331 ------MVLLGMLLWGQWAMPVQAFAEDAEEQPARQQSMNASYRQQLLALADADQKIRAEIRGAVT-------AQQLQANQGAAKEMAMRLVA-----SQ 82
1203760654 ------MVLLGMLLWGQWAMPVQAFAEDAEEQPARQQSMNASYRQQLLALADADQKIRAEIRGAVT-------AQQLQANQGAAKEMAMRLVA-----SQ 82
1203785350 ------MVLLGMLLWGQWAMPVQAFAEDAEEQPARQQSMNASYRQQLLALADADQKIRAEIRGAVT-------AQQLQANQGAAKEMAMRLVA-----SQ 82
1203805937 ------MVLLGMLLWGQWAMPVQAFAEDAEEQPARQQSMNASYRQQLLALADADQKIRAEIRGAVT-------AQQLQANQGAAKEMAMRLVA-----SQ 82
1203792350 ------MVLLGMLLWGQWAMPVQAFAEDAEEQPARQQSMNASYRQQLLALADADQKIRAEIRGAVT-------AQQLQANQGAAKEMAMRLVA-----SQ 82
1203852386 ------MVLLGMLLWGQWAMPVQAFAEDAEEQPARQQSMNASYRQQLLALADADQKIRAEIRGAVT-------AQQLQANQGAAKEMAMRLVA-----SQ 82
1203773865 ------MVLLGMLLWGQWAMPVQAFAEDAEEQPARQQSMNASYRQQLLALADADQKIRAEIRGAVT-------AQQLQANQGAAKEMAMRLVA-----SQ 82
1203741733 ------MVLLGMLLWGQWAMPVQAFAEDAEEQPARQQSMNASYRQQLLALADADQKIRAEIRGAVT-------AQQLQANQGAAKEMAMRLVA-----SQ 82
1203848578 ------MVLLGMLLWGQWAMPVQAFAEDAEEQPARQQSMNASYRQQLLALADVDQKIRAEIRGAVT-------AQQLQANQGAAKEMAMRLVA-----SQ 82
1203948816 ------MVLLGMLLWGQWAMPVQAFAEDAEEQPARQQSMNASYRQQLLALADVDQKIRAEIRGAVT-------AQQLQANQGAAKEMAMRLVA-----SQ 82
1203891105 ------MVLLGMLLWGQWAMPVQAFAEDAEEQPARQQSMNASYRQQLLALADVDQKIRAEIRGAVT-------AQQLQANQGAAKEMAMRLVA-----SQ 82
1203752654 ------MVLLGMLLWGQWAMPVQAFAEDAEEQPARQQSMNASYRQQLLALADVDQKIRAEIRGAVT-------AQQLQANQGAAKEMAMRLVA-----SQ 82
1203955172 ------MVLLGMLLWGQWAMPVQAFAEDAEEQPARQQSMNASYRQQLLALADVDQKIRAEIRGAVT-------AQQLQANQGAAKEMAMRLVA-----SQ 82
1203885689 ------MVLLGMLLWGQWAMPVQAFAEDAEEQPARQQSMNASYRQQLLALADVDQKIRAEIRGAVT-------AQQLQANQGAAKEMAMRLVA-----SQ 82
1203813176 ------MVLLGMLLWGQWAMPVQAFAEDAEEQPARQQSMNASYRQQLLALADVDQKIRAEIRGAVT-------AQQLQANQGAAKEMAMRLVA-----SQ 82
1203894760 ------MVLLGMLLWGQWAMPVQAFAEDAEEQPARQQSMNASYRQQLLALADVDQKIRAEIRGAVT-------AQQLQANQGAAKEMAMRLVA-----SQ 82
1203714212 ------MVLLGMLLWGQWAMPVQAFAEDAEEQPARQQSMNASYRQQLLALADVDQKIRAEIRGAVT-------AQQLQANQGAAKEMAMRLVA-----SQ 82
1203794393 ------MVLLGMLLWGQWAMPVQAFAEDAEEQPARQQSMNASYRQQLLALADVDQKIRAEIRGAVT-------AQQLQANQGAAKEMAMRLVA-----SQ 82
1203978383 ------MVLLGMLLWGQWAMPVQAFAEDAEEQPARQQSMNASYRQQLLALADVDQKIRAEIRGAVT-------AQQLQANQGAAKEMAMRLVA-----SQ 82
1203825023 ------MVLLGMLLWGQWAMPVQAFAEDAEEQPARQQSMNASYRQQLLALADVDQKIRAEIRGAVT-------AQQLQANQGAAKEMAMRLVA-----SQ 82
1203988106 ------MVLLGMLLWGQWAMPVQAFAEDAEEQPARQQSMNASYRQQLLALADVDQKIRAEIRGAVT-------AQQLQANQGAAKEMAMRLVA-----SQ 82
1114737733 ----MKMVLLGMLLWGQWAMPVQAFAEDAEEQPARQQSMNASYRQQLLALADVDQKIRAEIRGAVT-------AQQLQANQGAAKEMAMRLVA-----SQ 84
1203871641 ----MKMVLLGMLLWGQWAMPVQAFAEDAEEQPARQQSMNASYRQQLLALADVDQKIRAEIRGAVT-------AQQLQANQGAAKEMAMRLVA-----SQ 84
1203765404 ----MKMVLLGMLLWGQWAMPVQAFAEDAEEQPARQQSMNASYRQQLLALADVDQKIRAEIRGAVT-------AQQLQANQGAAKEMAMRLVA-----SQ 84
1203936693 ----MKMVLLGMLLWGQWAMPVQAFAEDAEEQPARQQSMNASYRQQLLALADVDQKIRAEIRGAVT-------AQQLQANQGAAKEMAMRLVA-----SQ 84
1203914376 ----MKMVLLGMLLWGQWAMPVQAFAEDAEEQPARQQSMNASYRQQLLALADVDQKIRAEIRGAVT-------AQQLQANQGAAKEMAMRLVA-----SQ 84
1203897480 ----MKMVLLGMLLWGQWAMPVQAFAEDAEEQPARQQSMNASYRQQLLALADVDQKIRAEIRGAVT-------AQQLQANQGAAKEMAMRLVA-----SQ 84
1203736703 ----MKMVLLGMLLWGQWAMPVQAFAEDAEEQPARQQSMNASYRQQLLALADVDQKIRAEIRGAVT-------AQQLQANQGAAKEMAMRLVA-----SQ 84
1203923455 ----MKMVLLGMLLWGQWAMPVQAFAEDAEEQPARQQSMNASYRQQLLALADVDQKIRAEIRGAVT-------AQQLQANQGAAKEMAMRLVA-----SQ 84
1203714688 ----MKMVLLGMLLWGQWAMPVQAFAEDAEEQPARQQSMNASYRQQLLALADVDQKIRAEIRGAVT-------AQQLQANQGAAKEMAMRLVA-----SQ 84
1203932523 ----MKMVLLGMLLWGQWAMPVQAFAEDAEEQPARQQSMNASYRQQLLALADVDQKIRAEIRGAVT-------AQQLQANQGAAKEMAMRLVA-----SQ 84
1203927777 ----MKMVLLGMLLWGQWAMPVQAFAEDAEEQPARQQSMNASYRQQLLALADVDQKIRAEIRGAVT-------AQQLQANQGAAKEMAMRLVA-----SQ 84
1203878120 ----MKMVLLGMLLWGQWAMPVQAFAEDAEEQPARQQSMNASYRQQLLALADVDQKIRAEIRGAVT-------AQQLQANQGAAKEMAMRLVA-----SQ 84
1203882381 ----MKMVLLGMLLWGQWAMPVQAFAEDAEEQPARQQSMNASYRQQLLALADVDQKIRAEIRGAVT-------AQQLQANQGAAKEMAMRLVA-----SQ 84
1203981868 ----MKMVLLGMLLWGQWAMPVQAFAEDAEEQPARQQSMNASYRQQLLALADVDQKIRAEIRGAVT-------AQQLQANQGAAKEMAMRLVA-----SQ 84
1203910183 ----MKMVLLGMLLWGQWAMPVQAFAEDAEEQPARQQSMNASYRQQLLALADVDQKIRAEIRGAVT-------AQQLQANQGAAKEMAMRLVA-----SQ 84
1203919133 ----MKMVLLGMLLWGQWAMPVQAFAEDAEEQPARQQSMNASYRQQLLALADVDQKIRAEIRGAVT-------AQQLQANQGAAKEMAMRLVA-----SQ 84
1203917928 ----MKMVLLGMLLWGQWAMPVQAFAEDAEEQPARQQSMNASYRQQLLALADVDQKIRAEIRGAVT-------AQQLQANQGAAKEMAMRLVA-----SQ 84
1203960538 ----MKMVLLGMLLWGQWAMPVQAFAEDAEEQPARQQSMNASYRQQLLALADVDQKIRAEIRGAVT-------AQQLQANQGAAKEMAMRLVA-----SQ 84
1203708059 ----MKMVLLGMLLWGQWAMPVQAFAEDAEEQPARQQSMNASYRQQLLALADVDQKIRAEIRGAVT-------AQQLQANQGAAKEMAMRLVA-----SQ 84
1203798348 ----MKMVLLGMLLWGQWAMPVQAFAEDAEEQPARQQSMNASYRQQLLALADVDQKIRAEIRGAVT-------AQQLQANQGAAKEMAMRLVA-----SQ 84
1203901836 ----MKMVLLGMLLWGQWAMPVQAFAEDAEEQPARQQSMNASYRQQLLALADVDQKIRAEIRGAVT-------AQQLQANQGAAKEMAMRLVA-----SQ 84
1203754395 ----MKMVLLGMLLWGQWAMPVQAFAEDAEEQPARQQSMNASYRQQLLALADVDQKIRAEIRGAVT-------AQQLQANQGAAKEMAMRLVA-----SQ 84
1203817186 ----MKMVLLGMLLWGQWAMPVQAFAEDAEEQPARQQSMNASYRQQLLALADVDQKIRAEIRGAVT-------AQQLQANQGAAKEMAMRLVA-----SQ 84
1203940998 ----MKMVLLGMLLWGQWAMPVQAFAEDAEEQPARQQSMNASYRQQLLALADVDQKIRAEIRGAVT-------AQQLQANQGAAKEMAMRLVA-----SQ 84
1203857187 ----MKMVLLGMLLWGQWAMPVQAFAEDAEEQPARQQSMNASYRQQLLALADADQKIRAEIRGAVT-------AQQLQANQGAAKEMAMRLVA-----SQ 84
1203821751 ----MKMVLLGMLLWGQWAMPVQAFAEDAEEQPARQQSMNASYRQQLLALADADQKIRAEIRGAVT-------AQQLQANQGAAKEMAMRLVA-----SQ 84
1203966786 ----MKMVLLGMLLWGQWAMPVQAFAEDAEEQPARQQSMNASYRQQLLALADADQKIRAEIRGAVT-------AQQLQANQGAAKEMAMRLVA-----SQ 84
1203862519 ----MKMVLLGMLLWGQWAMPVQAFAEDAEEQPARQQSMNASYRQQLLALADADQKIRAEIRGAVT-------AQQLQANQGAAKEMAMRLVA-----SQ 84
1203835242 ----MKMVLLGMLLWGQWAMPVQAFAEDAEEQPARQQSMNASYRQQLLALADADQKIRAEIRGAVT-------AQQLQANQGAAKEMAMRLVA-----SQ 84
1203729303 ----MKMVLLGMLLWGQWAMPVQAFAEDAEEQPARQQSMNASYRQQLLALADADQKIRAEIRGAVT-------AQQLQANQGAAKEMAMRLVA-----SQ 84
1203779155 ----MKMVLLGMLLWGQWAMPVQAFAEDAEEQPARQQSMNASYRQQLLALADADQKIRAEIRGAVT-------AQQLQANQGAAKEMAMRLVA-----SQ 84
1203747334 ----MKMVLLGMLLWGQWAMPVQAFAEDAEEQPARQQSMNASYRQQLLALADADQKIRAEIRGAVT-------AQQLQANQGAAKEMAMRLVA-----SQ 84
1203719954 ----MKMVLLGMLLWGQWAMPVQAFAEDAEEQPARQQSMNASYRQQLLALADADQKIRAEIRGAVT-------AQQLQANQGAAKEMAMRLVA-----SQ 84
1203767106 ----MKMVLLGMLLWGQWAMPVQAFAEDAEEQPARQQSMNASYRQQLLALADADQKIRAEIRGAVT-------AQQLQANQGAAKEMAMRLVA-----SQ 84
1203724032 ----MKMVLLGMLLWGQWAMPVQAFAEDAEEQPARQQSMNASYRQQLLALADADQKIRAEIRGAVT-------AQQLQANQGAAKEMAMRLVA-----SQ 84
1203963496 ----MKMVLLGMLLWGQWAMPVQAFAEDAEEQPARQQSMNASYRQQLLALADADQKIRAEIRGAVT-------AQQLQANQGAAKEMAMRLVA-----SQ 84
1203867268 ----MKMVLLGMLLWGQWAMPVQAFAEDAEEQPARQQSMNASYRQQLLALADADQKIRAEIRGAVT-------AQQLQANQGAAKEMAMRLVA-----SQ 84
1203736652 ----MKMVLLGMLLWGQWAMPVQAFAEDAEEQPARQQSMNASYRQQLLALADADQKIRAEIRGAVT-------AQQLQANQGAAKEMAMRLVA-----SQ 84
1203783979 ----MKMVLLGMLLWGQWAMPVQAFAEDAEEQPARQQSMNASYRQQLLALADADQKIRAEIRGAVT-------AQQLQANQGAAKEMAMRLVA-----SQ 84
1203831230 ----MKMVLLGMLLWGQWAMPVQAFAEDAEEQPARQQSMNASYRQQLLALADADQKIRAEIRGAVT-------AQQLQANQGAAKEMAMRLVA-----SQ 84
1203837418 ----MKMVLLGMLLWGQWAMPVQAFAEDAEEQPARQQSMNASYRQQLLALADADQKIRAEIRGAVT-------AQQLQANQGAAKEMAMRLVA-----SQ 84
1203974736 ----MKMVLLGMLLWGQWAMPVQAFAEDAEEQPARQQSMNASYRQQLLALADADQKIRAEIRGAVT-------AQQLQANQGAAKEMAMRLVA-----SQ 84
1203866957 ----MKMVLLGMLLWGQWAMPVQAFAEDAEEQPARQQSMNASYRQQLLALADADQKIRAEIRGAVT-------AQQLQANQGAAKEMAMRLVA-----SQ 84
1203844643 ----MKMVLLGMLLWGQWAMPVQAFAEDAEEQPARQQSMNASYRQQLLALADADQKIRAEIRGAVT-------AQQLQANQGAAKEMAMRLVA-----SQ 84
1203808660 ----MKMVLLGMLLWGQWAMPVQAFAEDAEEQPARQQSMNASYRQQLLALADADQKIRAEIRGAVT-------AQQLQANQGAAKEMAMRLVA-----SQ 84
1084153285 ----MKMVLLGMLLWGQWAMPVQAFAEDAEEQPARQQSMNASYRQQLLALADADQKIRAEIRGAVT-------AQQLQANQGAAKEMAMRLVA-----SQ 84
1084167088 ----MKMVLLGMLLWGQWAMPVQAFAEDAEEQPARQQSMNASYRQQLLALADADQKIRAEIRGAVT-------AQQLQANQGAAKEMAMRLVA-----SQ 84
1082907377 ----MKMVLLGMLLWGQWAMPVQAFAEDAEEQPARQQSMNASYRQQLLALADADQKIRAEIRGAVT-------AQQLQANQGAAKEMAMRLVA-----SQ 84
1084162744 ----MKMVLLGMLLWGQWAMPVQAFAEDAEEQPARQQSMNASYRQQLLALADADQKIRAEIRGAVT-------AQQLQANQGAAKEMAMRLVA-----SQ 84
1082902114 ----MKMVLLGMLLWGQWAMPVQAFAEDAEEQPARQQSMNASYRQQLLALADADQKIRAEIRGAVT-------AQQLQANQGAAKEMAMRLVA-----SQ 84
1082897639 ----MKMVLLGMLLWGQWAMPVQAFAEDAEEQPARQQSMNASYRQQLLALADADQKIRAEIRGAVT-------AQQLQANQGAAKEMAMRLVA-----SQ 84
1085622206 ----MKMVLLGMLLWGQWAMPVQAFAEDAEEQPARQQSMNASYRQQLLALADADQKIRAEIRGAVT-------AQQLQANQGAAKEMAMRLVA-----SQ 84
1084157524 ----MKMVLLGMLLWGQWAMPVQAFAEDAEEQPARQQSMNASYRQQLLALADADQKIRAEIRGAVT-------AQQLQANQGAAKEMAMRLVA-----SQ 84
1082143499 ----MKMVLLGMLLWGQWAMPVQAFAEDAEEQPARQQSMNASYRQQLLALADADQKIRAEIRGAVT-------AQQLQANQGAAKEMAMRLVA-----SQ 84
1904335826 ------MVLLGMLLWGQWAMPVQAFAEDAEEQPARQQSMNASYRQQLLALADADQKIRAEIRGAVT-------AQQLQANQGAAKEMAMRLVA-----SQ 82
1104577954 ----MKMVLLGMLLWGQWAMPVQAFAEDAEEQPARQQSMNASYRQQLLALADADQKIRAEIRGAVT-------AQQLQANQGAAKEMAMRLVA-----SQ 84
1806099227 ----MKMVLLGMLLWGQWAMPVQAFAEDAEEQPARQQSMNASYRQQLLALADADQKIRAEIRGAVT-------AQQLQANQGAAKEMAMRLVA-----SQ 84
1501657983 --------------MKNLIKPIVLFVASIFAYINVYGQIDTILRDKINQMAKKDQDWMMNPPKNAN----------------GNDYFFKRMEE-----IY 65
1090975247 -------------------------MKQSYPPVTTKKVDIKRIAKRLILMAKKDQQIRDKYFSKNE-----------------KKYLDKMWK------VD 52
1806863047 ---------------------------------MTCEPRRPDIARSLLGRAETARELRGKLLRGLL-----------------SESETSMARH-----TD 45
1091236563 --------------------------------------------------------MRASNKWDES--------------------------------ID 12
```

```
1091273272 --------------------------------------------------------MRASNKWDES--------------------------------ID 12
1091169967 ------------------------------------MAVYKRYADNIVSMAFMDQEARKNPKISIS-----------------------ELKK-----ID 36
1091193520 ------------------------------------MAVYKRYADNIVSMAFMDQEARKNPKISIS-----------------------ELKK-----ID 36
1091190115 ------------------------------------MAVYKRYADNIVSMAFMDQEARKNPKISIS-----------------------ELKK-----ID 36
1091254628 -------------------------------------MKYSKIAKQINEFAKNDQLLRNRIFSKPS----------------ISEEDKKALED-----FD 42
1091035071 -------------------------------------MKYSKIAKQINEFAKNDQLLRNRIFSKPS----------------ISEEDKKALED-----FD 42
1091203206 -------------------------------------MKYSKIAKQINEFAKNDQLLRNRIFSKPS----------------ISEEDKKALED-----FD 42
1091210742 -------------------------------------MKYSKIAKQINEFAKNDQLLRNRIFSKPS----------------ISEEDKKALED-----FD 42
1091205158 -------------------------------------MKYSKIAKQINEFAKNDQLLRNRIFSKPS----------------ISEEDKKALED-----FD 42
1091137286 -------------------------------------MKYSKIAKQINEFAKNDQLLRNRIFSKPS----------------ISEEDKKALED-----FD 42
1091173617 -------------------------------------MKYSKIAKQINEFAKNDQLLRNRIFSKPS----------------ISEEDKKALED-----FD 42
1091234491 -------------------------------------MKYSKIAKQINEFAKNDQLLRNRIFSKPS----------------ISEEDKKALED-----FD 42
1091150044 -------------------------------------MKYSKIAKQINEFAKNDQLLRNRIFSKPS----------------ISEEDKKALED-----FD 42
1091199422 -------------------------------------MKYSKIAKQINEFAKNDQLLRNRIFSKPS----------------ISEEDKKALED-----FD 42
1091208725 -------------------------------------MKYSKIAKQINEFAKNDQLLRNRIFSKPS----------------ISEEDKKALED-----FD 42
1008483832 -------------------------------------MKYSKIAKQINEFAKNDQLLRNRIFSKPS----------------ISEEDKKALED-----FD 42
1091132924 -----------------------------------MTPKYSKVAKQINDFAKNDQLLRNKIFSKSS----------------ISGKDKNALAD-----FD 44
1091105593 -------------------------------------MKYSKVAKQINDFAKNDQLLRNKIFSKSS----------------ISGKDKNALAD-----FD 42
1701924761 --------------------------MMITACKTKNIPYSEKMKTDLKVMYKKDQDIQNLIINTSN-------------EKVDKSELERKKYA-----IF 56
1504402626 -----------------------------------VEHDIAALAAELADMGAADQRSSAGANSDDP-------------------ARQLAWRR-----LT 41
1502349055 -----------------------------------VTHDVAALAVELMDMAAADHRTAVHANSDDP-------------------AEQLAWRR-----LT 41
1091155140 ----------------------------------MEKFKNKALAELISSMAKMDQLERSRVRQKQK--------------------LYSGLAK-----SD 41
1115923480 -----------------------------------------------------MPVGRDPERWRRV------------------------VTP-----VD 18
1505624847 -------------------------------MPDPTPPQRRAVADELLRRMATDQHARGVRENGPS-----------------VAPDNDLMRT-----VD 47
1091169964 ----------------------------------MKSKLNQKLIKEIKEMFDIDQELRNSWVNKKL-TEPLISWGEDDEGRKPKTKMGLGLANYLVYLID 65
1091193517 ----------------------------------MKSKLNQKLIKEIKEMFDIDQELRNSWVNKKL-TEPLISWGEDDEGRKPKTKMGLGLANYLVYLID 65
1091190112 ----------------------------------MKSKLNQKLIKEIKEMFDIDQELRNSWVNKKL-TEPLISWGEDDEGRKPKTKMGLGLANYLVYLID 65
1091068289 ------------------------------------MVPNKKIKESLLLMIERDQKMRKLFMKKRS-----------------------NWNAN----VD 37
1091000951 -------------------------------------------------MIERDQKMRKLFMKKRS-----------------------NWNAN----VD 24
1090982346 ------------------------------------MVPNKKIKESLLLMIERDQKMRKLFMKKRS-----------------------NWNAN----VD 37
1091056744 ------------------------------------MVPNKKIKESLLLMIERDQKMRKLFMKKRS-----------------------NWNAN----VD 37
1091004012 -------------------------------------------------MIERDQKMRKLFMKKRS-----------------------NWNAN----VD 24
1090984164 ------------------------------------MVPNKKIKESLLLMIERDQKMRKLFMKKRS-----------------------NWNAN----VD 37
1091392317 ------------------------------------MSIDRKIRDKIQQMAKEDQLVRRQQWRDVQLLNKRHLDRKNNEYKRVIAGLIKKLRI-----LD 59
1091393958 ---------------------------------------------------------------------------------------------------- 0

                                               H                                  D
1064363960 SLNYLSILKYLKE-YGYPVFKSKNPKVIPAIRTVCLH-Q--SFNKQLELFPYLYKAYNDGFINSEEFSFLLNRMHIMK-----YGESY-----------P 170
1091210526 TITTNILKFLIGT-LGTDFVCKLGESGVNRFITLSCH-SR-NLKFIESICES--DEILKCTSDREKVAILIDNALVRSGRKQRFGEIMQIHKNIEGKSVS 130
1091100979 TITTNILKFLIGT-LGTDFVCKLGESGVNRFITLSCH-SR-DLKFIESICES--DEILKCTSDREKVAILIDNALVRSGKKQRFGEIMQIHKNMDGKSVS 103
1091209206 TITTNILKFLIGT-LGTDFVCKLGESGVNRFITLSCH-SR-DLKFIESICES--DEILKCTSDREKVAILIDNALVRSGKKQRFGEIMQIHKNMDGKSVS 103
1091136844 TITTNILKFLIGT-LGTDFVCKLGESGVNRFITLSCH-SR-DLKFIESICES--DEILKCTSDREKVAILIDNALVRSGKKQRFGEIMQIHKNMDGKSVS 103
1091234973 TITTNILKFLIGT-LGTDFVCKLGESGVNRFITLSCH-SR-DLKFIESICES--DEILKCTSDREKVAILIDNALVRSGKKQRFGEIMQIHKNMDGKSVS 103
1091111073 TITTNILKFLIGT-LGTDFVCKLGESGVNRFITLSCH-SR-DLKFIESICES--DEILKCTSDREKVAILIDNALVRSGKKQRFGEIMQIHKNMDGKSVS 103
1090995448 TITTNILKFLIGT-LGTDFVCKLGESGVNRFITLSCH-SR-DLKFIESICES--DEILKCTSDREKVAILIDNALVRSGKKQRFGEIMQIHKNMDGKSVS 103
1091125220 TITTNILKFLIGT-LGTDFVCKLGESGVNRFITLSCH-SR-DLKFIESICES--DEILKCTSDREKVAILIDNALVRSGKKQRFGEIMQIHKNMDGKSVS 103
1091055562 TITTNILKFLIGT-LGTDFVCKLGESGVNRFITLSCH-SR-DLKFIESICES--DEILKCTSDREKVAILIDNALVRSGKKQRFGEIMQIHKNMDGKSVS 103
1091167037 TITTNILKFLIGT-LGTDFVCKLGESGVNRFITLSCH-SR-DLKFIESICES--DEILKCTSDREKVAILIDNALVRSGKKQRFGEIMQIHKNMDGKSVS 130
1017537694 RINEEKVLHIIDT-YGWLGKSDIGEMANQTLWLVIQH-AA--IDVQEKYLPQLEASVKKGASEGWHLAFLEDRIRMRKGKKQRYGTQAK-KDKLSGITYI 170
1507123407 RKNIKIVTKIIDS-LGWLGKDKIGKDANLALFIAIQH-AD-KLSTMEKYLPMLKEAANKGNAEKGQAAYLIDRVELLNKRKQIYGTQYS-IKENGT---A 154
1506709129 RKNIKIVTKIIDS-LGWLGKDKIGKDANLALFIAIQH-AD-KLSTMEKYLPMLKEAANKGNAEKGQAAYLIDRVELLNKRKQIYGTQYS-IKENGT---A 154
1028378095 RKNIKIVTKIIDS-LGWLGKDKIGKDANLALFIAIQH-AD-KLSTMEKYLPMLKEAANKGNAEKGQAAYLIDRVELLNKRKQIYGTQYS-IKENGT---A 154
1604649744 RKNIKIVTKIIDS-LGWLGEDKIGHDANLALFVALQH-AD-KLETMEKYLPVLKEAANKNNAEKGQAAYLIDRVELLNNRKQIYGTQYS-IRENGT---A 148
1604693139 ADNTKRAELLYKK-YGYLGFDKVGEKGETQFWVIVQH-SDKSVDFQNKVLKSLKKEVSKKNASPDNYALLYDRVKVNNGEKQLFGTQVE-YRKNGQAKPK 164
1034294064 ERHLSYLRQELGDSVAIPSVGEVGRDGLAALWLLVQH-ADDDPSLQAKALANFEPLVKRGELDASKFALLSDRVLLASGKPQRFGTQLR-SLETGE---P 187
1701082198 ARNLRYLHKELGHAVSIPSIDEVGRDGLAALWLLIQH-ADGDVELQSKALKKFEPMVKSGEIDASKFALLSDRVLLASGKPQRFGSQLR-SPTTGE---P 161
1701091927 ARNLRYLHKELGHAVSIPSIDEVGRDGLAALWLLIQH-ADGDVELQSKALKKFEPMVKSGEIDASKFALLSDRVLLASGKPQRFGSQLR-SPTTGE---P 161
1701098556 ARNLRYLHKELGHAVSIPSIDEVGRDGLAALWLLIQH-ADGDVELQSKALKKFEPMVKSGEIDASKFALLSDRVLLASGKPQRFGSQLR-SPTTGE---P 161
1701107844 ARNLRYLHKELGHAVSIPSIDEVGRDGLAALWLLIQH-ADGDVELQSKALKKFEPMVKSGEIDASKFALLSDRVLLASGKPQRFGSQLR-SPTTGE---P 161
1701085478 ARNLRYLHKELGHAVSIPSIDEVGRDGLAALWLLIQH-ADGDVELQSKALKKFEPMVKSGEIDASKFALLSDRVLLASGKPQRFGSQLR-SPTTGE---P 161
1701063269 ARNLRYLHKELGHAVSIPSIDEVGRDGLAALWLLIQH-ADGDVELQSKALKKFEPMVKSGEIDASKFALLSDRVLLASGKPQRFGSQLR-SPTTGE---P 161
1701088627 ARNLRYLHKELGHAVSIPSIDEVGRDGLAALWLLIQH-ADGDVELQSKALKKFEPMVKSGEIDASKFALLSDRVLLASGKPQRFGSQLR-SPTTGE---P 161
1701069525 ARNLRYLHKELGHAVSIPSIDEVGRDGLAALWLLIQH-ADGDVELQSKALKKFEPMVKSGEIDASKFALLSDRVLLASGKPQRFGSQLR-SPTTGE---P 161
1003372624 ARNLRYLHKELGHAVSIPSIDEVGRDGLAALWLLIQH-ADGDVELQSKALKKFEPMVKSGEIDASKFALLSDRVLLASGKPQRFGSQLR-SPTTGE---P 187
1200090063 ARNLRYLHKELGHAVSIPSIDEVGRDGLAALWLLIQH-ANGDIELQSKALKKFEPMVKSGEIDASKFALLSDRVLLASGKPQRFGSQLR-SLTTGE---S 161
1000489224 ARNLRYLHKELGHAVSIPSIDEVGRDGLAALWLLIQH-ANGDIELQSKALKKFEPMVKSGEIDASKFALLSDRVLLASGKPQRFGSQLL-SLTTGE---P 187
1000112493 ARNLRYLHKELGHAVSIPSIDEVGRDGLAALWLLIQH-ANGDIELQSKALKKFEPMVKSGEIDASKFALLSDRVLLASGKPQRFGSQLL-SLTTGE---P 187
1101210601 ARNLRYLHKELGHALSIPSIDEVGRDGLAALWLLIQH-ANGDIELQSKALRKFEPMVKSGEIDASKFALLSDRVLLASGKPQRFGSQLQ-SLTTGE---P 187
1091018262 HISTDVIKALVDK-FGLPTPENVGVEAARYFIVLLIH-TS-DMSYVSTLSNT--PEFRRVDYNKTDLAILKDRILLFKGKKQCFGTVVTTKKDKKGKFVT 129
1091256281 HISTDVIKALVDK-FGLPTPENVGVEAARYFIVLLIH-TS-DMSYVSTLSNT--PEFRRVDYNKTDLAILKDRILLFKGKKQCFGTVVTTKKDKKGKFVT 129
1091176042 HISTDVIKALVDK-FGLPTPENVGVEAARYFIVLLIH-TS-DMSYVSTLSNT--PEFRRVDYNKTDLAILKDRILLFKGKKQCFGTVVTTKKDKKGKFVT 129
1033412857 TRHTGALKAIIRR-CGWPDAARYDAPTARAAWLLAQH-ADHDVRFQMRVLKLIRGSIAGDADSRKEFAYLSDRISLKQKGKQVYGSQLMVRDSGIAELPP 163
1303959073 KQNAHELREIIKL-NGWPLISTHGAQAAHDAWLVAQH-S--DLELQEAVLRTIKPLLAIKEVAPRHYAYLADRVALKKTGKQRFGTQ---MECAQGRLVL 120
1300606538 QQNQQALSALMAK-YGFPDTAVAGSDGAHAGFLVAQH-AA-DRTFRDAFLTGIQAAAEKGRYSKEDLALYVDRNRVLSGQPQLYGTQ---HKADGS---L 177
1049540699 RENQAALSALIEK-FGFPDVALVGEDGAHAGFLVAQH-ST-DRKFRDEFLKNIEVAAAGGSYSTADMALFVDRNLIMSGKPQRYGTQ---RKADGS---L 170
1081417263 RENQVALSALMDK-FGFPDIASVGQDGAHAGFLVAQH-ST-DREFRDAFLQKMQAASERGSYSTADLALFVDRNLVMSSKPQRYGTQ---RKADGS---L 175
1203949331 RENQAALSRLIDQ-FGFPDIPSVGEDGAHAGFLIAQH-ST-DRAFRDDFLQKMQAAAQRGAYSTADLALFIDRNLIMSGKPQRYGTQ---RRADGS---L 173
1203760654 RENQAALSRLIDQ-FGFPDIPSVGEDGAHAGFLIAQH-ST-DRAFRDDFLQKMQAAAQRGAYSTADLALFIDRNLIMSGKPQRYGTQ---RRADGS---L 173
1203785350 RENQAALSRLIDQ-FGFPDIPSVGEDGAHAGFLIAQH-ST-DRAFRDDFLQKMQAAAQRGAYSTADLALFIDRNLIMSGKPQRYGTQ---RRADGS---L 173
1203805937 RENQAALSRLIDQ-FGFPDIPSVGEDGAHAGFLIAQH-ST-DRAFRDDFLQKMQAAAQRGAYSTADLALFIDRNLIMSGKPQRYGTQ---RRADGS---L 173
1203792350 RENQAALSRLIDQ-FGFPDIPSVGEDGAHAGFLIAQH-ST-DRAFRDDFLQKMQAAAQRGAYSTADLALFIDRNLIMSGKPQRYGTQ---RRADGS---L 173
1203852386 RENQAALSRLIDQ-FGFPDIPSVGEDGAHAGFLIAQH-ST-DRAFRDDFLQKMQAAAQRGAYSTADLALFIDRNLIMSGKPQRYGTQ---RRADGS---L 173
1203773865 RENQAALSRLIDQ-FGFPDIPSVGEDGAHAGFLIAQH-ST-DRAFRDDFLQKMQAAAQRGAYSTADLALFIDRNLIMSGKPQRYGTQ---RRADGS---L 173
1203741733 RENQAALSRLIDQ-FGFPDIPSVGEDGAHAGFLIAQH-ST-DRAFRDDFLQKMQAAAQRGAYSTADLALFIDRNLIMSGKPQRYGTQ---RRADGS---L 173
1203848578 RENQAALSRLIDQ-FGFPDIPSVGEDGAHAGFLIAQH-ST-DRAFRDDFLQKMQAAAQRGAYSTADLALFIDRNLIMSGKPQRYGTQ---RRADGS---L 173
1203948816 RENQAALSRLIDQ-FGFPDIPSVGEDGAHAGFLIAQH-ST-DRAFRDDFLQKMQAAAQRGAYSTADLALFIDRNLIMSGKPQRYGTQ---RRADGS---L 173
1203891105 RENQAALSRLIDQ-FGFPDIPSVGEDGAHAGFLIAQH-ST-DRAFRDDFLQKMQAAAQRGAYSTADLALFIDRNLIMSGKPQRYGTQ---RRADGS---L 173
1203752654 RENQAALSRLIDQ-FGFPDIPSVGEDGAHAGFLIAQH-ST-DRAFRDDFLQKMQAAAQRGAYSTADLALFIDRNLIMSGKPQRYGTQ---RRADGS---L 173
1203955172 RENQAALSRLIDQ-FGFPDIPSVGEDGAHAGFLIAQH-ST-DRAFRDDFLQKMQAAAQRGAYSTADLALFIDRNLIMSGKPQRYGTQ---RRADGS---L 173
1203885689 RENQAALSRLIDQ-FGFPDIPSVGEDGAHAGFLIAQH-ST-DRAFRDDFLQKMQAAAQRGAYSTADLALFIDRNLIMSGKPQRYGTQ---RRADGS---L 173
1203813176 RENQAALSRLIDQ-FGFPDIPSVGEDGAHAGFLIAQH-ST-DRAFRDDFLQKMQAAAQRGAYSTADLALFIDRNLIMSGKPQRYGTQ---RRADGS---L 173
1203894760 RENQAALSRLIDQ-FGFPDIPSVGEDGAHAGFLIAQH-ST-DRAFRDDFLQKMQAAAQRGAYSTADLALFIDRNLIMSGKPQRYGTQ---RRADGS---L 173
1203714212 RENQAALSRLIDQ-FGFPDIPSVGEDGAHAGFLIAQH-ST-DRAFRDDFLQKMQAAAQRGAYSTADLALFIDRNLIMSGKPQRYGTQ---RRADGS---L 173
1203794393 RENQAALSRLIDQ-FGFPDIPSVGEDGAHAGFLIAQH-ST-DRAFRDDFLQKMQAAAQRGAYSTADLALFIDRNLIMSGKPQRYGTQ---RRADGS---L 173
1203978383 RENQAALSRLIDQ-FGFPDIPSVGEDGAHAGFLIAQH-ST-DRAFRDDFLQKMQAAAQRGAYSTADLALFIDRNLIMSGKPQRYGTQ---RRADGS---L 173
1203825023 RENQAALSRLIDQ-FGFPDIPSVGEDGAHAGFLIAQH-ST-DRAFRDDFLQKMQAAAQRGAYSTADLALFIDRNLIMSGKPQRYGTQ---RRADGS---L 173
```

```
1203988106 RENQAALSRLIDQ-FGFPDIPSVGEDGAHAGFLIAQH-ST-DRAFRDDFLQKMQAAAQRGAYSTADLALFIDRNLIMSGKPQRYGTQ---RRADGS---L 173
1114737733 RENQAALSRLIDQ-FGFPDIPSVGEDGAHAGFLIAQH-ST-DRAFRDDFLQKMQAAAQRGAYSTADLALFIDRNLIMSGKPQRYGTQ---RRADGS---L 175
1203871641 RENQAALSRLIDQ-FGFPDIPSVGEDGAHAGFLIAQH-ST-DRAFRDDFLQKMQAAAQRGAYSTADLALFIDRNLIMSGKPQRYGTQ---RRADGS---L 175
1203765404 RENQAALSRLIDQ-FGFPDIPSVGEDGAHAGFLIAQH-ST-DRAFRDDFLQKMQAAAQRGAYSTADLALFIDRNLIMSGKPQRYGTQ---RRADGS---L 175
1203936693 RENQAALSRLIDQ-FGFPDIPSVGEDGAHAGFLIAQH-ST-DRAFRDDFLQKMQAAAQRGAYSTADLALFIDRNLIMSGKPQRYGTQ---RRADGS---L 175
1203914376 RENQAALSRLIDQ-FGFPDIPSVGEDGAHAGFLIAQH-ST-DRAFRDDFLQKMQAAAQRGAYSTADLALFIDRNLIMSGKPQRYGTQ---RRADGS---L 175
1203897480 RENQAALSRLIDQ-FGFPDIPSVGEDGAHAGFLIAQH-ST-DRAFRDDFLQKMQAAAQRGAYSTADLALFIDRNLIMSGKPQRYGTQ---RRADGS---L 175
1203736703 RENQAALSRLIDQ-FGFPDIPSVGEDGAHAGFLIAQH-ST-DRAFRDDFLQKMQAAAQRGAYSTADLALFIDRNLIMSGKPQRYGTQ---RRADGS---L 175
1203923455 RENQAALSRLIDQ-FGFPDIPSVGEDGAHAGFLIAQH-ST-DRAFRDDFLQKMQAAAQRGAYSTADLALFIDRNLIMSGKPQRYGTQ---RRADGS---L 175
1203714688 RENQAALSRLIDQ-FGFPDIPSVGEDGAHAGFLIAQH-ST-DRAFRDDFLQKMQAAAQRGAYSTADLALFIDRNLIMSGKPQRYGTQ---RRADGS---L 175
1203932523 RENQAALSRLIDQ-FGFPDIPSVGEDGAHAGFLIAQH-ST-DRAFRDDFLQKMQAAAQRGAYSTADLALFIDRNLIMSGKPQRYGTQ---RRADGS---L 175
1203927777 RENQAALSRLIDQ-FGFPDIPSVGEDGAHAGFLIAQH-ST-DRAFRDDFLQKMQAAAQRGAYSTADLALFIDRNLIMSGKPQRYGTQ---RRADGS---L 175
1203878120 RENQAALSRLIDQ-FGFPDIPSVGEDGAHAGFLIAQH-ST-DRAFRDDFLQKMQAAAQRGAYSTADLALFIDRNLIMSGKPQRYGTQ---RRADGS---L 175
1203882381 RENQAALSRLIDQ-FGFPDIPSVGEDGAHAGFLIAQH-ST-DRAFRDDFLQKMQAAAQRGAYSTADLALFIDRNLIMSGKPQRYGTQ---RRADGS---L 175
1203981868 RENQAALSRLIDQ-FGFPDIPSVGEDGAHAGFLIAQH-ST-DRAFRDDFLQKMQAAAQRGAYSTADLALFIDRNLIMSGKPQRYGTQ---RRADGS---L 175
1203910183 RENQAALSRLIDQ-FGFPDIPSVGEDGAHAGFLIAQH-ST-DRAFRDDFLQKMQAAAQRGAYSTADLALFIDRNLIMSGKPQRYGTQ---RRADGS---L 175
1203919133 RENQAALSRLIDQ-FGFPDIPSVGEDGAHAGFLIAQH-ST-DRAFRDDFLQKMQAAAQRGAYSTADLALFIDRNLIMSGKPQRYGTQ---RRADGS---L 175
1203917928 RENQAALSRLIDQ-FGFPDIPSVGEDGAHAGFLIAQH-ST-DRAFRDDFLQKMQAAAQRGAYSTADLALFIDRNLIMSGKPQRYGTQ---RRADGS---L 175
1203960538 RENQAALSRLIDQ-FGFPDIPSVGEDGAHAGFLIAQH-ST-DRAFRDDFLQKMQAAAQRGAYSTADLALFIDRNLIMSGKPQRYGTQ---RRADGS---L 175
1203708059 RENQAALSRLIDQ-FGFPDIPSVGEDGAHAGFLIAQH-ST-DRAFRDDFLQKMQAAAQRGAYSTADLALFIDRNLIMSGKPQRYGTQ---RRADGS---L 175
1203798348 RENQAALSRLIDQ-FGFPDIPSVGEDGAHAGFLIAQH-ST-DRAFRDDFLQKMQAAAQRGAYSTADLALFIDRNLIMSGKPQRYGTQ---RRADGS---L 175
1203901836 RENQAALSRLIDQ-FGFPDIPSVGEDGAHAGFLIAQH-ST-DRAFRDDFLQKMQAAAQRGAYSTADLALFIDRNLIMSGKPQRYGTQ---RRADGS---L 175
1203754395 RENQAALSRLIDQ-FGFPDIPSVGEDGAHAGFLIAQH-ST-DRAFRDDFLQKMQAAAQRGAYSTADLALFIDRNLIMSGKPQRYGTQ---RRADGS---L 175
1203817186 RENQAALSRLIDQ-FGFPDIPSVGEDGAHAGFLIAQH-ST-DRAFRDDFLQKMQAAAQRGAYSTADLALFIDRNLIMSGKPQRYGTQ---RRADGS---L 175
1203940998 RENQAALSRLIDQ-FGFPDIPSVGEDGAHAGFLIAQH-ST-DRAFRDDFLQKMQAAAQRGAYSTADLALFIDRNLIMSGKPQRYGTQ---RRADGS---L 175
1203857187 RENQAALSRLIDQ-FGFPDIPSVGEDGAHAGFLIAQH-ST-DRAFRDDFLQKMQAAAQRGAYSTADLALFIDRNLIMSGKPQRYGTQ---RRADGS---L 175
1203821751 RENQAALSRLIDQ-FGFPDIPSVGEDGAHAGFLIAQH-ST-DRAFRDDFLQKMQAAAQRGAYSTADLALFIDRNLIMSGKPQRYGTQ---RRADGS---L 175
1203966786 RENQAALSRLIDQ-FGFPDIPSVGEDGAHAGFLIAQH-ST-DRAFRDDFLQKMQAAAQRGAYSTADLALFIDRNLIMSGKPQRYGTQ---RRADGS---L 175
1203862519 RENQAALSRLIDQ-FGFPDIPSVGEDGAHAGFLIAQH-ST-DRAFRDDFLQKMQAAAQRGAYSTADLALFIDRNLIMSGKPQRYGTQ---RRADGS---L 175
1203835242 RENQAALSRLIDQ-FGFPDIPSVGEDGAHAGFLIAQH-ST-DRAFRDDFLQKMQAAAQRGAYSTADLALFIDRNLIMSGKPQRYGTQ---RRADGS---L 175
1203729303 RENQAALSRLIDQ-FGFPDIPSVGEDGAHAGFLIAQH-ST-DRAFRDDFLQKMQAAAQRGAYSTADLALFIDRNLIMSGKPQRYGTQ---RRADGS---L 175
1203779155 RENQAALSRLIDQ-FGFPDIPSVGEDGAHAGFLIAQH-ST-DRAFRDDFLQKMQAAAQRGAYSTADLALFIDRNLIMSGKPQRYGTQ---RRADGS---L 175
1203747334 RENQAALSRLIDQ-FGFPDIPSVGEDGAHAGFLIAQH-ST-DRAFRDDFLQKMQAAAQRGAYSTADLALFIDRNLIMSGKPQRYGTQ---RRADGS---L 175
1203719954 RENQAALSRLIDQ-FGFPDIPSVGEDGAHAGFLIAQH-ST-DRAFRDDFLQKMQAAAQRGAYSTADLALFIDRNLIMSGKPQRYGTQ---RRADGS---L 175
1203767106 RENQAALSRLIDQ-FGFPDIPSVGEDGAHAGFLIAQH-ST-DRAFRDDFLQKMQAAAQRGAYSTADLALFIDRNLIMSGKPQRYGTQ---RRADGS---L 175
1203724032 RENQAALSRLIDQ-FGFPDIPSVGEDGAHAGFLIAQH-ST-DRAFRDDFLQKMQAAAQRGAYSTADLALFIDRNLIMSGKPQRYGTQ---RRADGS---L 175
1203963496 RENQAALSRLIDQ-FGFPDIPSVGEDGAHAGFLIAQH-ST-DRAFRDDFLQKMQAAAQRGAYSTADLALFIDRNLIMSGKPQRYGTQ---RRADGS---L 175
1203867268 RENQAALSRLIDQ-FGFPDIPSVGEDGAHAGFLIAQH-ST-DRAFRDDFLQKMQAAAQRGAYSTADLALFIDRNLIMSGKPQRYGTQ---RRADGS---L 175
1203736652 RENQAALSRLIDQ-FGFPDIPSVGEDGAHAGFLIAQH-ST-DRAFRDDFLQKMQAAAQRGAYSTADLALFIDRNLIMSGKPQRYGTQ---RRADGS---L 175
1203783979 RENQAALSRLIDQ-FGFPDIPSVGEDGAHAGFLIAQH-ST-DRAFRDDFLQKMQAAAQRGAYSTADLALFIDRNLIMSGKPQRYGTQ---RRADGS---L 175
1203831230 RENQAALSRLIDQ-FGFPDIPSVGEDGAHAGFLIAQH-ST-DRAFRDDFLQKMQAAAQRGAYSTADLALFIDRNLIMSGKPQRYGTQ---RRADGS---L 175
1203837418 RENQAALSRLIDQ-FGFPDIPSVGEDGAHAGFLIAQH-ST-DRAFRDDFLQKMQAAAQRGAYSTADLALFIDRNLIMSGKPQRYGTQ---RRADGS---L 175
1203974736 RENQAALSRLIDQ-FGFPDIPSVGEDGAHAGFLIAQH-ST-DRAFRDDFLQKMQAAAQRGAYSTADLALFIDRNLIMSGKPQRYGTQ---RRADGS---L 175
1203866957 RENQAALSRLIDQ-FGFPDIPSVGEDGAHAGFLIAQH-ST-DRAFRDDFLQKMQAAAQRGAYSTADLALFIDRNLIMSGKPQRYGTQ---RRADGS---L 175
1203844643 RENQAALSRLIDQ-FGFPDIPSVGEDGAHAGFLIAQH-ST-DRAFRDDFLQKMQAAAQRGAYSTADLALFIDRNLIMSGKPQRYGTQ---RRADGS---L 175
1203808660 RENQAALSRLIDQ-FGFPDIPSVGEDGAHAGFLIAQH-ST-DRAFRDDFLQKMQAAAQRGAYSTADLALFIDRNLIMSGKPQRYGTQ---RRADGS---L 175
1084153285 RENQAALSRLIDQ-FGFPDIPSVGEDGAHAGFLIAQH-ST-DRAFRDDFLQKMQAAAQRGAYSTADLALFIDRNLIMSGKPQRYGTQ---RRADGS---L 175
1084167088 RENQAALSRLIDQ-FGFPDIPSVGEDGAHAGFLIAQH-ST-DRAFRDDFLQKMQAAAQRGAYSTADLALFIDRNLIMSGKPQRYGTQ---RRADGS---L 175
1082907377 RENQAALSRLIDQ-FGFPDIPSVGEDGAHAGFLIAQH-ST-DRAFRDDFLQKMQAAAQRGAYSTADLALFIDRNLIMSGKPQRYGTQ---RRADGS---L 175
1084162744 RENQAALSRLIDQ-FGFPDIPSVGEDGAHAGFLIAQH-ST-DRAFRDDFLQKMQAAAQRGAYSTADLALFIDRNLIMSGKPQRYGTQ---RRADGS---L 175
1082902114 RENQAALSRLIDQ-FGFPDIPSVGEDGAHAGFLIAQH-ST-DRAFRDDFLQKMQAAAQRGAYSTADLALFIDRNLIMSGKPQRYGTQ---RRADGS---L 175
1082897639 RENQAALSRLIDQ-FGFPDIPSVGEDGAHAGFLIAQH-ST-DRAFRDDFLQKMQAAAQRGAYSTADLALFIDRNLIMSGKPQRYGTQ---RRADGS---L 175
1085622206 RENQAALSRLIDQ-FGFPDIPSVGEDGAHAGFLIAQH-ST-DRAFRDDFLQKMQAAAQRGAYSTADLALFIDRNLIMSGKPQRYGTQ---RRADGS---L 175
1084157524 RENQAALSRLIDQ-FGFPDIPSVGEDGAHAGFLIAQH-ST-DRAFRDDFLQKMQAAAQRGAYSTADLALFIDRNLIMSGKPQRYGTQ---RRADGS---L 175
1082143499 RENQAALSRLIDQ-FGFPDIPSVGEDGAHAGFLIAQH-ST-DRAFRDDFLQKMQAAAQRGAYSTADLALFIDRNLIMSGKPQRYGTQ---RRADGS---L 175
1904335826 RENQAALSRLIDQ-FGFPDIPSVGEEGAHAGFLIAQH-ST-DRAFRDDFLQKMQAAAQRGAYSTADLALFIDRNLIMSGKPQRYGTQ---RRADGS---L 173
1104577954 RENQAALSRLIDQ-FGFPDIPSVGEEGAHAGFLIAQH-ST-DRAFRDDFLQKMQAAAQRGAYSTADLALFIDRNLIMSGKPQRYGTQ---RRADGS---L 175
1806099227 RENQAALSRLIDQ-FGFPDIPSVGEEGAHAGFLIAQH-ST-DRAFRDDFLQKMQAAAQRGAYSTADLALFIDRNLIMSGKPQRYGTQ---RRADGS---L 175
1501657983 RENYEDAKVIFSK-FGFPGYNKVGKDGSDKFWLVVQH-LNKWPDFQTLVLKKMAAEVKRKNASADKYAYLLDRINLNSGELQIYGTQIW-YDIRYHRTFP 162
1090975247 EANQKEFKEIFNE-TGL-ITSKYGSEAQMAAFLIVQHMPREDVKFMKKYLTLMKKSMA--GYPTNIYAMLVDRVRNWEGKDQLYGTQFMPVEGLESTYKL 148
1806863047 HANAQVLRVVAD-HGWPGRSLVGDEAAKAAWQIALH-ADHLPDFQRLVLRLMATAVERGEVPIQRWAHLYDRCSVNDGGTQLYGTQYR-LGADGV-VEV 141
1091236563 KKNIKELKKIVYK-VGWPDKKLVGVKAALGAWLIVQH-GVFNIEFQKYCLGLLKKAVADKKMPQFTVPFLIDRIKMVEGKPQLFGTQFF-KKTRSGKLHL 109
1091273272 KKNIKELKKIVYK-VGWPDKKLVGVKAALGAWLIVQH-GVFNIEFQKYCLGLLKKAVADKKMPQFTVPFLIDRIKMVEGKPQLFGTQFF-KKTRSGKLHL 109
1091169967 NASTRALKQIVSK-IGWPTISKVGKEASQKAWLLLQH-TN-DIKFVGRCLNMMKKHIN--DIDKTSIPYLVDKINLTRGKNQYYGTIMS-SKVINNKIVI 130
1091193520 NASTRALKQIVSK-IGWPTISKVGKEASQKAWLLLQH-TN-DIKFVGRCLNMMKKHIN--DIDKTSIPYLVDKINLTRGKNQYYGTIMS-SKVINNKIVI 130
1091190115 NASTRALKQIVSK-IGWPTISKVGKEASQKAWLLLQH-TN-DIKFVGRCLNMMKKHIN--DIDKTSIPYLVDKINLTRGKNQYYGTIMS-SKVINNKIVI 130
1091254628 KNSSDFIKSIVSE-ICLPTITKVGKKASYNSWLLVQH-SR-DLKFQKSYLGLLKQNEK--DINPANIAYLEDRVLMYGGKPQIYGTQFVLNKKTGK-MVP 136
1091035071 KNSSDFIKSIVSE-ICLPTITKVGKKASYNSWLLVQH-SR-DLKFQKSYLGLLKQNEK--DINPANIAYLEDRVLMYGGKPQIYGTQFVLNKKTGK-MVP 136
1091203206 KNSSDFIKSIVSE-ICLPTITKVGKKASYNSWLLVQH-SR-DLKFQKSYLGLLKQNEK--DINPANIAYLEDRVLMYGGKPQIYGTQFVLNKKTGK-MVP 136
1091210742 KNSSDFIKSIVSE-ICLPTITKVGKKASYNSWLLVQH-SR-DLKFQKSYLGLLKQNEK--DINPANIAYLEDRVLMYGGKPQIYGTQFVLNKKTGK-MVP 136
1091205158 KNSSDFIKSIVSE-ICLPTITKVGKKASYNSWLLVQH-SR-DLKFQKSYLGLLKQNEK--DINPANIAYLEDRVLMYGGKPQIYGTQFVLNKKTGK-MVP 136
1091137286 KNSSDFIKSIVSE-ICLPTITKVGKKASYNSWLLVQH-SR-DLKFQKSYLGLLKQNEK--DINPANIAYLEDRVLMYGGKPQIYGTQFVLNKKTGK-MVP 136
1091173617 KNSSDFIKSIVSE-ICLPTITKVGKKASYNSWLLVQH-SR-DLKFQKSYLGLLKQNEK--DINPANIAYLEDRVLMYGGKPQIYGTQFVLNKKTGK-MVP 136
1091234491 KNSSDFIKSIVSE-ICLPTITKVGKKASYNSWLLVQH-SR-DLKFQKSYLGLLKQNEK--DINPANIAYLEDRVLMYGGKPQIYGTQFVLNKKTGK-MVP 136
1091150044 KNSSDFIKSIVSE-ICLPTITKVGKKASYNSWLLVQH-SR-DLKFQKSYLGLLKQNEK--DINPANIAYLEDRVLMYGGKPQIYGTQFVLNKKTGK-MVP 136
1091199422 KNSSDFIKSIVSE-ICLPTITKVGKKASYNSWLLVQH-SR-DLKFQKSYLGLLKQNEK--DINPANIAYLEDRVLMYGGKPQIYGTQFVLNKKTGK-MVP 136
1091208725 KNSSDFIKSIVSE-ICLPTITKVGKKASYNSWLLVQH-SR-DLKFQKSYLGLLKQNEK--DINPANIAYLEDRVLMYGGKPQIYGTQFVLNKKTGK-MVP 136
1008483832 KNSSDFIKSIVSE-ICLPTITKVGKKASYNSWLLVQH-SR-DLKFQKSYLGLLKQNEK--DINPANIAYLEDRVLMYGGKPQIYGTQFVLNKKTGK-MVP 136
1091132924 KKSSDFIKNIVIE-IGLPTITKVGKNASYNAWLLVQH-SR-DLEFQKSYLELLKQNEK--DINPANIAYLEDRVLMYENKPQIFGTQFVLNQKTGK-MEP 138
1091105593 KKSSDFIKNIVIE-IGLPTITKVGKNASYNAWLLVQH-SR-DLEFQKSYLELLKQNEK--DINPANIAYLEDRVLMYENKPQIFGTQFVLNQKTGK-MEP 136
1701924761 KTNCSTLKNYFEE-YGYPTINHNGKKISHTFWLLVQH-CDHDIDFQKSVLKAMKSELKNNNVAKVDYAYLYDRVQINNGKKQLYGTQVG-YDENYN---P 150
1504402626 ARHGDRLGEIMDA-YGWPTADLVGEEAARAAWLIAQH-ADRQLDVQRRALDLMGQAVSAGRAGARELAFLRDRTLVNEGREQVYGTQIA-GVRDGA-PVP 137
1502349055 ARHGDRLGEIMAE-HGWPTAELVGEEAARAAWLIAQH-ADRQLDIQRRALRLMQQAVSAGAAGPRELAFLCDRTLVNEGRHQVYGTQIA-GVKDGA-PVP 137
1091155140 MARLRQMKRIVNK-YGWTTRKLVGKRASHSAWLLVQH-GDDDVKFQEYCLKLIKQA------------------------------------GEND---- 99
1115923480 QDNAAWLSAVVDE-YGWPGRGLVGRDGAHAAWLLLQH-APHDL--QQRCLPLLREAVAAGEAEAAELAYLEDRVRCHEGMPQRYGTQYL-RLPDGE-VRI 112
1505624847 TDNTAALARILDE-YGWPGLSLVGEQATTAAWLIAQH-A--ELDFQLRALNMLRDAVARGEAPAWHFAYLTDRCLMHRREPQLFGTQYL-DTGDGHGLRL 142
1091169964 ATHNTKIQRIINK-YGYPISNLIGKKGMFNFFILVQH-QDYDLELQKNCLQY-------CDFAKKDRAHLIDRVLLHEGKEQLYGTQFQ-RDAENNRMTS 155
1091193517 ATHNTKIQRIINK-YGYPISNLIGKKGMFNFFILVQH-QDYDLELQKNCLQY-------CDFAKKDRAHLIDRVLLHEGKEQLYGTQFQ-RDAENNRMTS 155
1091190112 ATHNTKIQRIINK-YGYPISNLIGKKGMFNFFILVQH-QDYDLELQKNCLQY-------CDFAKKDRAHLIDRVLLHEGKEQLYGTQFQ-RDAENNRMTS 155
1091068289 MKNTEKLKKIINK-YGWPGKSLVGEKAADAAWLIAQH-ADHDVKFQEKCLCLIKKAVKIGEASKKNLAYLIDRMLVKNRKKQIYGTQFR-YESEQNLLKP 134
1091000951 MKNTEKLKKIINK-YGWPGKSLVGEKAADAAWLIAQH-ADHDVKFQEKCLCLIKKAVKIGEASKKNLAYLIDRMLVKNRKKQIYGTQFR-YESEQNLLKP 121
1090982346 MKNTEKLKKIINK-YGWPGKSLVGEKAADAAWLIAQH-ADHDVKFQEKCLCLIKKAVKIGEASKKNLAYLIDRMLVKNRKKQIYGTQFR-YESEQNLLKP 134
1091056744 MKNTEKLKKIINK-YGWPGKSLVGEKAADAAWLIAQH-ADHDVKFQEKCLCLIKKAVKIGEASKKNLAYLIDRMLVKNRKKQIYGTQFR-YESEQNLLKP 134
1091004012 MKNTEKLKKIINK-YGWPGKSLVGEKAADAAWLIAQH-ADHDVKFQEKCLCLIKKAVKIGEASKKNLAYLIDRMLVKNRKKQIYGTQFR-YESEQNLLKP 121
1090984164 MKNTEKLKKIINK-YGWPGKSLVGEKAADAAWLIAQH-ADHDVKFQEKCLCLIKKAVKIGEASKKNLAYLIDRMLVKNRKKQIYGTQFR-YESEQNLLKP 134
```

```
1091392317 AKHTREMKKITTR-CDWPGRSLVGKRGAAAAWLLVQH-ADHDLVFQKKCLILMKSAAP-DEVESKHIAYLTDRILVHEGKEQIYGTQFK-SGPDNN-YAP 154
1091393958 ----------------------MGKRGAAAAWLLVQH-ADHDLVFQKKCLILMKSAAP-DEVESKHIAYLTDRILVHEGKEQIYGTQFK-SGPDNN-YAP 74
                         R
1064363960 HA--ISYEENIEQLLEKLQL---------------------------------------------- 188
1091210526 EPLSLQDPKNINKIRADFGLSKSLE--EHIKWANEQFENMKVPD---------------------- 172
1091100979 EPLPLQNPKNVNKIRADFGLSQSLE--EHIKWANEQFENMKVPD---------------------- 145
1091209206 EPLPLQNPKNVNKIRADFGLSQSLE--EHIKWANEQFENMKVPD---------------------- 145
1091136844 EPLPLQNPKNVNKIRADFGLSQSLE--EHIKWANEQFENMKVPD---------------------- 145
1091234973 EPLPLQNPKNVNKIRADFGLSQSLE--EHIKWANEQFENMKVPD---------------------- 145
1091111073 EPLPLQNPKNVNKIRADFGLSQSLE--EHIKWANEQFENMKVPD---------------------- 145
1090995448 EPLPLQNPKNVNKIRADFGLSQSLE--EHIKWANEQFENMKVPD---------------------- 145
1091125220 EPLPLQNPKNVNKIRADFGLSQSLE--EHIKWANEQFENMKVPD---------------------- 145
1091055562 EPLPLQNPKNVNKIRADFGLSQSLE--EHIKWANEQFENMKVPD---------------------- 145
1091167037 EPLPLQNPKNVNKIRADFGLSQSLE--EHIKWANEQFENMKVPD---------------------- 172
1017537694 YP--IENKETVNQRRKSIGLNT-IE--EYAKNNNYVIR---------------------------- 203
1507123407 FIENLIDSANVNSRRKSMDLYP-IE--KYIRMIDSSNSVENTQKIR-------------------- 197
1506709129 FIENLIDSANVNSRRKSMDLYP-IE--KYIRMIDSSNSVENTQKIR-------------------- 197
1028378095 FIENLIDSANVNSRRKSMDLYP-IE--KYIRMIDSSNSVENTQKIR-------------------- 197
1604649744 FIENLIDSANVNSRRKSMDLYP-IE--KYIEMIDSSNNATRSR----------------------- 188
1604693139 IG--LIDSINVDGWRVKYNLEP-LK--KYLNFMTERNFNINKNILIKKGITEPVFYK--------- 216
1034294064 LK--LDSPASVERERTALGLMS-LE--DYRCISDQLYRSAR------------------------- 223
1701082198 LD--VGNSVAIERERDALGLMK-LA--DYRCISEQLYKNSH------------------------- 197
1701091927 LD--VGNSVAIERERDALGLMK-LA--DYRCISEQLYKNSH------------------------- 197
1701098556 LD--VGNSVAIERERDALGLMK-LA--DYRCISEQLYKNSH------------------------- 197
1701107844 LD--VGNSVAIERERDALGLMK-LA--DYRCISEQLYKNSH------------------------- 197
1701085478 LD--VGNSVAIERERDALGLMK-LA--DYRCISEQLYKNSH------------------------- 197
1701063269 LD--VGNSVAIERERDALGLMK-LA--DYRCISEQLYKNSH------------------------- 197
1701088627 LD--VGNSVAIERERDALGLMK-LA--DYRCISEQLYKNSH------------------------- 197
1701069525 LD--VGNSVAIERERDALGLMK-LA--DYRCISEQLYKNSH------------------------- 197
1003372624 LD--VGNSVAIERERDALGLMK-LA--DYRCISEQLYKNSH------------------------- 223
1200090063 LD--LGNPVAIERERDALGLMK-LA--DYRCISEQLYKNSH------------------------- 197
1000489224 LD--LGNPVAIERERDALGLMK-LA--DYRCISEQLYKNSH------------------------- 223
1000112493 LD--LGNPVAIERERDALGLMK-LA--DYRCISEQLYKNSH------------------------- 223
1101210601 LD--LGNPVAIERERDALGLMK-LA--DYRCISEQLYKNSH------------------------- 223
1091018262 KPLPIEDDKNVDKRRQKYGLPP-LA--DYIKVSEETFNNFIKGKPK-------------------- 172
1091256281 KPLPIEDDKNVDKRRQKYGLPP-LA--DYIKVSEETFNNFIKGKPK-------------------- 172
1091176042 KPLPIEDDKNVDKRRQKYGLPP-LA--DYIKVSEETFNNFIKGKPK-------------------- 172
1033412857 GE--IDSVEAVDRRRAAIGLPG-VA--EYVKSANEKLARIRADRDKPDAP---------------- 208
1303959073 AP--LETPSLLAQRRREIGLSSALH--EKSMDEEAQC----------------------------- 153
1300606538 FD--VEQPERLQQRRAEMGLPPDVA--AH------------------------------------- 202
1049540699 FE--LEDPARLAQRRAEIGLPD-EQ--SGSGGLEP------------------------------- 200
1081417263 FE--LENPAQLRQRRTEAGLPD--E----------------------------------------- 196
1203949331 FE--LENPAELKKRRAEAGLPE-EE--DSVRGR--------------------------------- 201
1203760654 FE--LENPAELKKRRAEAGLPE-EE--DSVRGR--------------------------------- 201
1203785350 FE--LENPAELKKRRAEAGLPE-EE--DSVRGR--------------------------------- 201
1203805937 FE--LENPAELKKRRAEAGLPE-EE--DSVRGR--------------------------------- 201
1203792350 FE--LENPAELKKRRAEAGLPE-EE--DSVRGR--------------------------------- 201
1203852386 FE--LENPAELKKRRAEAGLPE-EE--DSVRGR--------------------------------- 201
1203773865 FE--LENPAELKKRRAEAGLPE-EE--DSVRGR--------------------------------- 201
1203741733 FE--LENPAELKKRRAEAGLPE-EE--DSVRGR--------------------------------- 201
1203848578 FE--LEDPAELKKRRAEAGLPE-EE--DSVRGR--------------------------------- 201
1203948816 FE--LENPAELKKRRAEAGLPE-EE--DSVRGR--------------------------------- 201
1203891105 FE--LENPAELKKRRAEAGLPE-EE--DSVRGR--------------------------------- 201
1203752654 FE--LENPAELKKRRAEAGLPE-EE--DSVRGR--------------------------------- 201
1203955172 FE--LENPAELKKRRAEAGLPE-EE--DSVRGR--------------------------------- 201
1203885689 FE--LENPAELKKRRAEAGLPE-EE--DSVRGR--------------------------------- 201
1203813176 FE--LENPAELKKRRAEAGLPE-EE--DSVRGR--------------------------------- 201
1203894760 FE--LENPAELKKRRAEAGLPE-EE--DSVRGR--------------------------------- 201
1203714212 FE--LENPAELKKRRAEAGLPE-EE--DSVRGR--------------------------------- 201
1203794393 FE--LENPAELKKRRAEAGLPE-EE--DSVRGR--------------------------------- 201
1203978383 FE--LENPAELKKRRAEAGLPE-EE--DSVRGR--------------------------------- 201
1203825023 FE--LENPAELKKRRAEAGLPE-EE--DSVRGR--------------------------------- 201
1203988106 FE--LENPAELKKRRAEAGLPE-EE--DSVRGR--------------------------------- 201
1114737733 FE--LENPAELKKRRAEAGLPE-EE--DSVRGR--------------------------------- 203
1203871641 FE--LENPAELKKRRAEAGLPE-EE--DSVRGR--------------------------------- 203
1203765404 FE--LENPAELKKRRAEAGLPE-EE--DSVRGR--------------------------------- 203
1203936693 FE--LENPAELKKRRAEAGLPE-EE--DSVRGR--------------------------------- 203
1203914376 FE--LENPAELKKRRAEAGLPE-EE--DSVRGR--------------------------------- 203
1203897480 FE--LENPAELKKRRAEAGLPE-EE--DSVRGR--------------------------------- 203
1203736703 FE--LENPAELKKRRAEAGLPE-EE--DSVRGR--------------------------------- 203
1203923455 FE--LENPAELKKRRAEAGLPE-EE--DSVRGR--------------------------------- 203
1203714688 FE--LENPAELKKRRAEAGLPE-EE--DSVRGR--------------------------------- 203
1203932523 FE--LENPAELKKRRAEAGLPE-EE--DSVRGR--------------------------------- 203
1203927777 FE--LENPAELKKRRAEAGLPE-EE--DSVRGR--------------------------------- 203
1203878120 FE--LENPAELKKRRAEAGLPE-EE--DSVRGR--------------------------------- 203
1203882381 FE--LENPAELKKRRAEAGLPE-EE--DSVRGR--------------------------------- 203
1203981868 FE--LENPAELKKRRAEAGLPE-EE--DSVRGR--------------------------------- 203
1203910183 FE--LENPAELKKRRAEAGLPE-EE--DSVRGR--------------------------------- 203
1203919133 FE--LENPAELKKRRAEAGLPE-EE--DSVRGR--------------------------------- 203
1203917928 FE--LENPAELKKRRAEAGLPE-EE--DSVRGR--------------------------------- 203
1203960538 FE--LENPAELKKRRAEAGLPE-EE--DSVRGR--------------------------------- 203
1203708059 FE--LENPAELKKRRAEAGLPE-EE--DSVRGR--------------------------------- 203
1203798348 FE--LENPAELKKRRAEAGLPE-EE--DSVRGR--------------------------------- 203
1203901836 FE--LENPAELKKRRAEAGLPE-EE--DSVRGR--------------------------------- 203
1203754395 FE--LENPAELKKRRAEAGLPE-EE--DSVRGR--------------------------------- 203
1203817186 FE--LENPAELKKRRAEAGLPE-EE--DSVRGR--------------------------------- 203
1203940998 FE--LENPAELKKRRAEAGLPE-EE--DSVRGR--------------------------------- 203
1203857187 FE--LENPAELKKRRAEAGLPE-EE--DSVRGR--------------------------------- 203
1203821751 FE--LENPAELKKRRAEAGLPE-EE--DSVRGR--------------------------------- 203
1203966786 FE--LENPAELKKRRAEAGLPE-EE--DSVRGR--------------------------------- 203
1203862519 FE--LENPAELKKRRAEAGLPE-EE--DSVRGR--------------------------------- 203
1203835242 FE--LENPAELKKRRAEAGLPE-EE--DSVRGR--------------------------------- 203
1203729303 FE--LENPAELKKRRAEAGLPE-EE--DSVRGR--------------------------------- 203
1203779155 FE--LENPAELKKRRAEAGLPE-EE--DSVRGR--------------------------------- 203
1203747334 FE--LENPAELKKRRAEAGLPE-EE--DSVRGR--------------------------------- 203
```

```
1203719954 FE--LENPAELKKRRAEAGLPE-EE--DSVRGR--------------------------------- 203
1203767106 FE--LENPAELKKRRAEAGLPE-EE--DSVRGR--------------------------------- 203
1203724032 FE--LENPAELKKRRAEAGLPE-EE--DSVRGR--------------------------------- 203
1203963496 FE--LENPAELKKRRAEAGLPE-EE--DSVRGR--------------------------------- 203
1203867268 FE--LENPAELKKRRAEAGLPE-EE--DSVRGR--------------------------------- 203
1203736652 FE--LENPAELKKRRAEAGLPE-EE--DSVRGR--------------------------------- 203
1203783979 FE--LENPAELKKRRAEAGLPE-EE--DSVRGR--------------------------------- 203
1203831230 FE--LENPAELKKRRAEAGLPE-EE--DSVRGR--------------------------------- 203
1203837418 FE--LENPAELKKRRAEAGLPE-EE--DSVRGR--------------------------------- 203
1203974736 FE--LENPAELKKRRAEAGLPE-EE--DSVRGR--------------------------------- 203
1203866957 FE--LENPAELKKRRAEAGLPE-EE--DSVRGR--------------------------------- 203
1203844643 FE--LENPAELKKRRAEAGLPE-EE--DSVRGR--------------------------------- 203
1203808660 FE--LENPAELKKRRAEAGLPE-EE--DSVRGR--------------------------------- 203
1084153285 FE--LENPAELKKRRAEAGLPE-EE--DSVRGR--------------------------------- 203
1084167088 FE--LENPAELKKRRAEAGLPE-EE--DSVRGR--------------------------------- 203
1082907377 FE--LENPAELKKRRAEAGLPE-EE--DSVRGR--------------------------------- 203
1084162744 FE--LENPAELKKRRAEAGLPE-EE--DSVRGR--------------------------------- 203
1082902114 FE--LENPAELKKRRAEAGLPE-EE--DSVRGR--------------------------------- 203
1082897639 FE--LENPAELKKRRAEAGLPE-EE--DSVRGR--------------------------------- 203
1085622206 FE--LENPAELKKRRAEAGLPE-EE--DSVRGR--------------------------------- 203
1084157524 FE--LENPAELKKRRAEAGLPE-EE--DSVRGR--------------------------------- 203
1082143499 FE--LENPAELKKRRAEAGLPE-EE--DSVRGR--------------------------------- 203
1904335826 FE--LENPAELKKRRAEAGLPE-EE--DSVRGH--------------------------------- 201
1104577954 FE--LENPAELKKRRAEAGLPE-EE--DSVRGH--------------------------------- 203
1806099227 FE--LENPAELKKRRAEAGLPE-EE--DSVRGH--------------------------------- 203
1501657983 YP--VVDIKRVNKRRKAVGLEA-LE--VYLNTTTESHFALRDSINLRSGVKKPWLYKVPSHMFGKY 223
1090975247 KE--LYRPNMVDKRRIELGLEP-LG--DYIKKLQEERGVTLIL----------------------- 186
1806863047 LP--VRDRQRLDSRRASVRLGPASAALDSLRHRHAREREADEPHDGAAALVGSAA----------- 194
1091236563 WK--IYNKKGIDKRRKKCGLEP-LK--NHKMVDQRIRYKYY------------------------- 145
1091273272 WK--IYNKKGIDKRRKKCGLEP-LK--NHKMVDQRIRYKYY------------------------- 145
1091169967 ITPKLRNKKSVNTRRRQFGLEP-LE--DQIKRNEKVFRKYFLNRK--------------------- 172
1091193520 ITPKLRNKKSVNTRRRQFGLEP-LE--DQIKRNEKVFRKYFLNRK--------------------- 172
1091190115 ITPKLRNKKSVNTRRRQFGLEP-LE--DQIKRNEKVFRKYFLNRK--------------------- 172
1091254628 YT--IWDAANVNERRSKYNLGK-LE--EHIKTFYSN------------------------------ 167
1091035071 YT--IWDAANVNERRSKYNLGK-LE--EHIKTFYSN------------------------------ 167
1091203206 YT--IWDAANVNERRSKYNLGK-LE--EHIKTFYSN------------------------------ 167
1091210742 YT--IWDAANVNERRSKYNLGK-LE--EHIKTFYSN------------------------------ 167
1091205158 YT--IWDAANVNERRSKYNLGK-LE--EHIKTFYSN------------------------------ 167
1091137286 YT--IWDAANVNERRSKYNLGK-LE--EHIKTFYSN------------------------------ 167
1091173617 YT--IWDAANVNERRSKYNLGK-LE--EHIKTFYSN------------------------------ 167
1091234491 YT--IWDAANVNERRSKYNLGK-LE--EHIKTFYSN------------------------------ 167
1091150044 YT--IWDAANVNERRSKYNLGK-LE--EHIKTFYSN------------------------------ 167
1091199422 YT--IWDAANVNERRSKYNLGK-LE--EHIKTFYSN------------------------------ 167
1091208725 YT--IWDAANVNERRSKYNLGK-LE--EHIKTFYSN------------------------------ 167
1008483832 YT--IWDAANVNERRSKYNLGK-LE--EHIKTFYSN------------------------------ 167
1091132924 YT--ISDPENVNERRSKYGMET-VE--EHLKKFYSV------------------------------ 169
1091105593 YT--ISDPENVNERRSKYGMET-VE--EHLKKFYSV------------------------------ 167
1701924761 YIKELINPSTVNKRRKAMGLNT-IE--EYIEEIRKIQTMNSKS----------------------- 190
1504402626 WP--CEDPERMDDLRVAVGIAP-FA--EYVAGFAPS------------------------------ 168
1502349055 WP--CEEPERVDELRAAVGVEP-FD--EYVARFS-------------------------------- 166
1091155140 -E--VANLPRLDDRRKTMGLGS-FD--EYKIKIEKEWKNESGRFRQESPV---------------- 143
1115923480 YE--VEDPEGLDERRAAVGLEP-HA--AYDARIRAMR----------------------------- 144
1505624847 WD--VADPDHLYARRAEVGLGP-HA--DYALARTVDAGC--------------------------- 176
1091169964 RP--IVDKKNVDARRKKMGLRP-LK--YDLVAMNKKFYNK-------------------------- 190
1091193517 RP--IVDKKNVDARRKKMGLRP-LK--YDLVAMNKKFYNK-------------------------- 190
1091190112 RP--IVDKKNVDARRKKMGLRP-LK--YDLVAMNKKFYNK-------------------------- 190
1091068289 YL--IRDKKNLARRRKNAGLES-FT--VNMKRLRLNVGLNKKNKRKNIKEV--------------- 180
1091000951 YL--IRDKKNLARRRKNAGLES-FT--VNMKRLRLNVGLNKKNKRKNIKEV--------------- 167
1090982346 YL--IRDKKNLARRRKNAGLES-FT--VNMKRLRLNVGLNKKNKRKNIKEV--------------- 180
1091056744 YL--IRDKKNLARRRKNAGLES-FT--VNMKRLRLNVGLNKKNKRKNIKEV--------------- 180
1091004012 YL--IRDKKNLARRRKNAGLES-FT--VNMKRLRLNVGLNKKNKRKNIKEV--------------- 167
1090984164 YL--IRDKKNLARRRKNAGLES-FT--VNMKRLRLNVGLNKKNKRKNIKEV--------------- 180
1091392317 FP--IKDPRRLNKLRKEIGLEP-FL--AYKKRMRALVS---------------------------- 187
1091393958 FP--IKDPRRLNKLRKEIGLEP-FL--AYKKRMRALVS---------------------------- 107
```

E.

Chryseoviridin precursors

```
WP_099768145.1 MKDKNSKKKPFFASFLEKQLKDPETVKGG---------GNITIPERDV-I-TKPFVDN--VTSPQDDLMQTMKYPSDGDDDFPSVPVD--------- 75
WP_048502161.1 MKDKNSKKKPFFASFLEKQLKDPETIKGG-------DTGIITDTLKDS-I-TKPSIDV--VTSPKDDMMHTLKYPSDGDDDAPTVPLD--------- 77
WP_034745973.1 MKDKNSKKKPFFASFLEKQLKDPETVKGG---------SNVTNILKDS-I-TNPAVDI--VTSPKDDLMVTMKYPSDGDDDAPTIPLD--------- 75
WP_048506193.1 MKNKNSKKQPFFASFLEKQLKDPETIKGG-------DTSIITNILKDS-I-TKPAVDV--VTSPQDDMMHTLKYPSDGDDDAPIIPLD--------- 77
WP_062648812.1 MKDKNSKKKPFFASFLEKQLKDPETVKGG-------DSGIITIPERDV-I-TKPSIDV--VTSPKDDMMHTLKYPSDGDDDAPTVPL---------- 76
WP_068944162.1 MENKNSKKKPFFASFLEKQLKDPETVKGG---------TIITIPERDV-I-TKPIIDD--VTSKLNDMEHTMKYPSDGDDDSAEL------------ 72
WP_079242686.1 MESNNSKKKPFFASFLEKQVKDPETVKGG---------SDITLAERDL-I-TKPTIDT--VTSPKDDMMHTLKYPSDGDDDTITLPL---------- 74
WP_002977592.1 MKSNNSKKKPFFASFLEKQVKDPETVKGG---------GDITLAETDL-I-TKPTVDT--VTSPKDDMMQTMKYPSDGDDDVINLPL---------- 74
WP_062696861.1 MKNKNSKKKPFFASFLEKQIRDPETVKGG-------GTEIITIPEKDV-I-TKPSVDT--ATSPKDDMAHTLKYPSDGDDDVPTLPL---------- 76
WP_034710238.1 MKNKNSQKKPFFASFLEKQLKDPQKVKGG--------TDIITIPERDA-V-TKPQIDS--VTSPKDDLAHTMKYPSDGDDYSPTV------------ 73
WP_072952774.1 MKNKDSKKKPFFASFLEKQLQDPEKIKGG---------GDITLATQDI-I-TKPTLDT--VTKPTLDMMQTQKYPSDGDDDTQYVDL---------- 74
WP_089790527.1 MKNKNSKKKPFFASFLEKQLQDPEKIKGG---------GDITLATQDI-V-TKPAFDG--VTKPDQDMAQTQKYPSDGDDDVQYVDL---------- 74
CdnA1          -MENRKSKKPFFASFLEKQIEDPEKIKGG--AVTSALVDNVTSVNKDN-V-TSSLLDH--VTKPGGDNV-TMKYPSDGDEDGNAV------------ 77
WP_029295142.1 MKNKNSKKQPFFASFLEKQLQDPEKIKGG---------GDVTIATQDGPITTSKTADA--VTKPIYDMAQTMKYPSDGDDDAPTV------------ 74
WP_034682457.1 MKNKNSKKKPFFASFLEKQLKDPQSVTGG--------GPDITIPERDV------------VTKPTVDLAHTMKYPSDGDDDAPTV------------ 65
WP_054513233.1 MKNKDSKKKPFFASFLEKQLKEPQKVKGG---------TDITIPERDS-V-YSPGP----VTMKPTDMAYTQKYPSDGDDDAPVV------------ 70
WP_089755757.1 MKNKNSKKKPFFASFLEKQVKDPETVKGG--------SGIITIPERDT------------VTKPTLDLAQTQKYPSDGDDDVQSV------------ 65
WP_027380633.1 MKNKNSKKKPFFAAFLEKQINEPQKVKGG---------GIITKPEVDT------------VTKPIYDMAQTQKYPSDGDDDVQSV------------ 64
WP_002977591.1 MENKNSKKKPFFATFLEKQVKDPETVKGG--GITSVLADQITTSLQDQ-I-TTPLKDN--VTKPDNDNV-TMKYPSDGDEDVLEV------------ 78
WP_034699857.1 MENKNSKKKPFFASFLEKQVKDPETVKGG--GITSVLADQITTSLQDQ-I-TTPIKDN--VTKPDNDNV-TMKYPSDGDEDVLEA------------ 78
WP_073294271.1 MKNKNSKKQPFFASFLEKQIQDPQTVKGG----------AITTALQDT-PTTSILKDT--VTDRQNDQV-TLKYPSDSDETGELE------------ 71
```

```
WP_034710235.1 MNNKKSQKKPFFASFLEKQLKDGQSVKGG----------VMTAVTLDA-PTTSVLKDD--VTGPGGDQV-TMKYPSDSDEAGYEV------------ 71
WP_034710236.1 MKNKKSQKKPFFASFLEKQVKDGQAVTGG----------AITSVLVDS-PTTSILRDS--ATTQSNDQV-TMKYPSDSDEAGELS------------ 71
CdnA3          MKNKNSKKEPFFAAFLEKQLQDPEKIQGG--GVSTSLKDVVTSPLGDT-L-TLKTLDN--GTTPAADTPVTLKYPSDSDEGGFDPGFPPGLDPEIHP 91

WP_099768147.1 -MEKKNSKKPFFASFLEKQIQDPETIQGG--GITTPTTDVLTVPVRDT-VTTSPFLDN--ATLPSRDQIVTMKYPSDSDESGELEIL---------- 81
WP_048502163.1 -MEKKNSKKPFFASFLEKQISDPEKIQGG--GITTSTTDVITIPERDN-VTTSPFLDN--ATTPLKDQAVTMKYPSDSDESGELDIL---------- 81
WP_062648816.1 -MEKKNSKKPFFASFLEKQINDPEKIQGG--GITTPTTDVLTVPQRDV-VTTSPFLDN--ATSQLRDQVVTMKYPSDSDESGELEVL---------- 81
WP_048506190.1 -MEKKNSKKPFFASFLEKQIQDPEKVQGG--GITTPTTDVLTVPTRDT-ITTSPFLDN--ATSTVRDQIVTMKYPSDSDESGELEVL---------- 81
WP_034745964.1 -MEKKNSKKPFFASFLEKQIQDPEKIQGG--GITTPTTDVITVPTRDV-VTTSPFLDN--ATLPVRDQIVTMKYPSDSDESGELEIL---------- 81
WP_034710233.1 -MEKKNSKKPFFASFLEKQIQDPEKIQGG--GITTPATDIVTSLAKDT-VTTSPTFDN--ATLPTKDNLVTLKYPSDNDEALDLD------------ 79
WP_089790526.1 -MKKNGGKKPFFASFLEKQIQNPEEIQGG-SVPTAPLTDFVTLPTKDT-VTTSPTLDN--ATLPFKDNVVTLKYPSDNDEIGDLSDPGES------- 85
WP_062696859.1 -MEKKNSKKPFFASFLEKQIQDPEKIQGG--GITDPSMDIVTLPAQDQ-I-TSVNFDT--VTLPVKDNVVTMKHPSDSDEAGELDYL---------- 80
WP_089755758.1 -MEKKNSKKPFFASFLEKQIKDPQAVQGG--GLTTPRTDIVTLPTNDT-I-TSPTFDN--VSTPTNDHV-TLKYPSDSDEAGDLS------------ 77
WP_062696860.1 -MKKNNSKKPFFASFLEKQIHDPEKVQGG-G--TTPDTDVITHPATDG-S-TSPVLDN--MTTPRQDYIETKKYPSDSDEAGNF------------- 76
WP_073294273.1 MKNENSKKKPFFASFLEKQIKDPETIQGG--AVTTPLTDIVSDTTRDV-A-TSPTFDN--ATLPTRDHLVTLKYPSDNDEAGI-------------- 77
WP_072952773.1 -MKSNDKKKPFFASFLEKQVQNPEEIQGGGGIITSINNDSVTLPTKDM-VVTTRTSDGPISTLPAYDTPVTLKYPSDSDEGGFNPGLPGLEPLEP-- 93
CdnA2          MKNKDSKKKPFFASFLEKQIQDPEKIKGG-------SGDITTGVLKDS-V-TSALVDN--ITKPGQDVV-SMKYPSDSDEDGDPV------------ 73
WP_034745967.1 MKDENSKKKPFFASFLEKQIQDPEKVLGG-G--TTPDTDVITSPVVDV-I-TAPILDN--ATSPSKDHLITLKYPSDHDDSVN-------------- 76
WP_048506191.1 MNNENSKKKPFFASFLEKQIKDPETIQGG-NIISSPDSDVITAPSIDN-V-TRPVFDN--VTAPSKDLLVTMKYPSDNDESGELEAF---------- 82
WP_048502162.1 MKDENSKKKPFFASFLEKQIKDPETVQGG-GIISNTDNDIVTAPSIDN-V-TAQIFDH--VTHPSKDLLVTMKYPSDGDDDVV-------------- 78
WP_048506192.1 MKDQNSKKKPFFASFLEKQIKDPETIQGG-GVITNTDNDVITLPSRDN-A-TAQILDH--VTSPNKDLLVTMKYPSDGDEDIV-------------- 78
WP_099768146.1 MKDENSKKKPFFASFLEKQIKDPETIQGG-GIITSTDNDIVTLPSRDN-I-TAQIFDH--VTSPNKDLLVTMKYPSDGDEDVI-------------- 78
WP_080780036.1 MTDQNSKKKPFFASFLEKQIQDPEKVQGG-GPITTPDTDIITAPSIDN-V-TRPVFDN--VTTPSKDLLVTMKYPSDGDESVL-------------- 78
WP_034745970.1 MKNENSKKKPFFASFLEKQIQDPEKVQGG-GIITTPEIDVITLPSIDN-V-TRPLLDN--ATNPSRDLLVTMKYPSDGDESVL-------------- 78
WP_034682458.1 -MKNKKQKKPFFASFLEKQIQDPEKVTGG-GTITTPSIDVVTIPTRDN-I-TSFVTDN--ITTPGNDHV-TLKYPSDSDESGPLE------------ 78
WP_073294276.1 -MRQENSKKPFFASFLEKQIKNPEKVNGG-LEITTALEESITIPTKDT-I-TSAILDN--VTKPGFDHV-TMKYPSDGDDSVV-------------- 76

WP_054513230.1 -MKNKKLKKPFFASFLEKQINDPEKVKGG----------SMTSELLDN-V-TTALQDT--VTKPGYDNV-TMKYPSDGDEAGETS------------ 69
WP_034674310.1 -MEKKKLKKPFFASFLENQIKDTKAVQGG--------------ASATG-I-TTPLKDT--VTKPAYDQQQTMKYPSDGDESGV-------------- 64
WP_089741992.1 -MEKKKLKKPFFASFLENQIKDTKAVKGG--------------ASGTG-L-TTPVKDT--VTKPVYDTHQTMKYPSDGDEGSDI------------- 65

WP_027380634.1 -MENKKSKKPFFASFLEKQVEDPEKIKGGSGTITSALQDNTTGALKDS-I-TSALQDN--ITKPGSDNV-TMKYPSDSDEGGDTV------------ 79
WP_072952772.1 -MGEKKNRKPFFASFLEKQIEDPEKIKGGSGSITSALQDNATAPLKDS-I-TSSLQDQ--VTKPGSDNV-TMKYPSDGDEDANAV------------ 79
WP_089790525.1 -MEEKKSKKPFFASFLEKQVEDPEKIKGGSGTITSALQDNTTGALKDS-I-TSALQDN--VTKPGSDNV-TMKYPSDGDEDGDAV------------ 79
CdnA4          MKSKDSKKKPFFASFLEKQIQDPEKVKGG---------TEVTLSTQDN-I-TKPLYDT--VTKPTLDMMQTMKYPSDGDDDAPVV------------ 72
WP_029295141.1 -MEGKKSKKPFFATFLEKQIQDPETVKGG--TVTSVLLDNTTGALKDS-V-TSTTIDN--ITKPGGDHV-TLKYPSDGDEDGTAI------------ 77
```
