# Supplementary figures and images for: Phylogenomic analysis of the diversity of graspetides and proteins involved in their biosynthesis
Source: Biol Direct. 2022 Mar 21;17:7. doi: 10.1186/s13062-022-00320-2 (PMC8939145; doi:10.1186/s13062-022-00320-2)

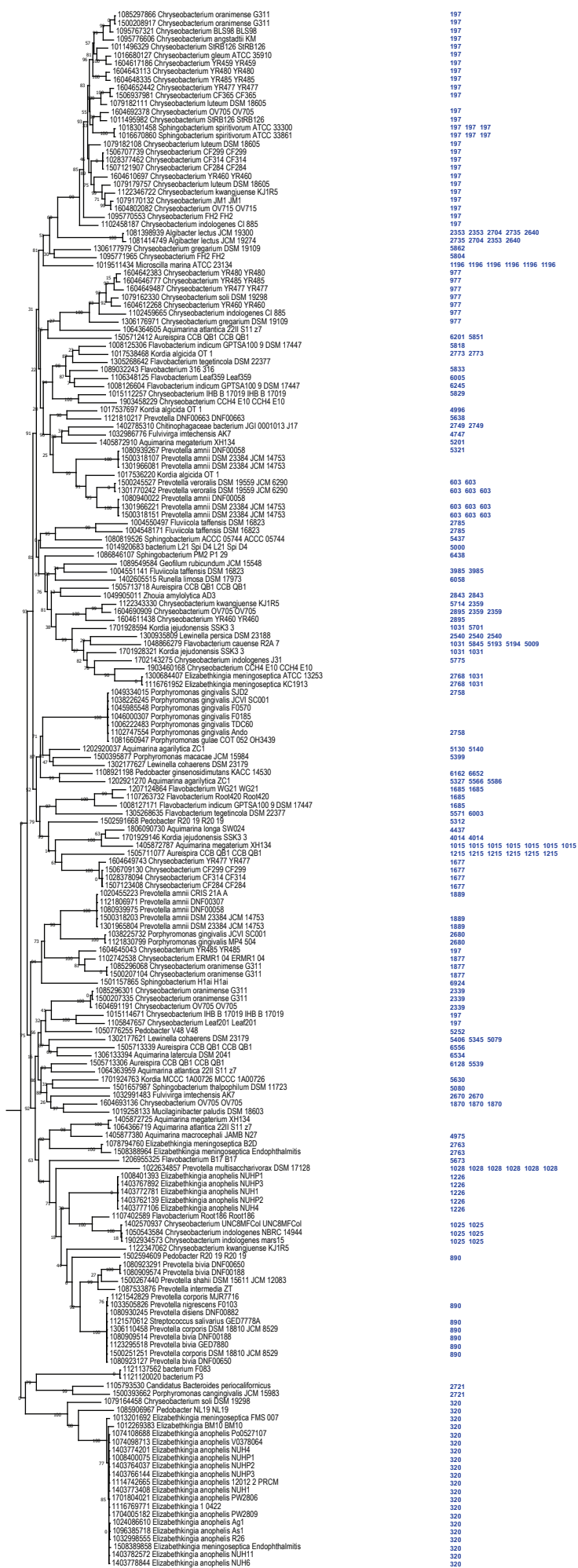

Supplementary Figure 3

Supplement: Supplementary file 3 — Additional file 3. Figure S3. Diversity of precursors associated with Branch 5 ATP-grasps. The ATP_grasp subtree corresponding to branch 5 is shown. Cluster number of precursors identified in the respective ATP-grasp loci are indicated on the right. [file 13062_2022_320_MOESM3_ESM.pdf]

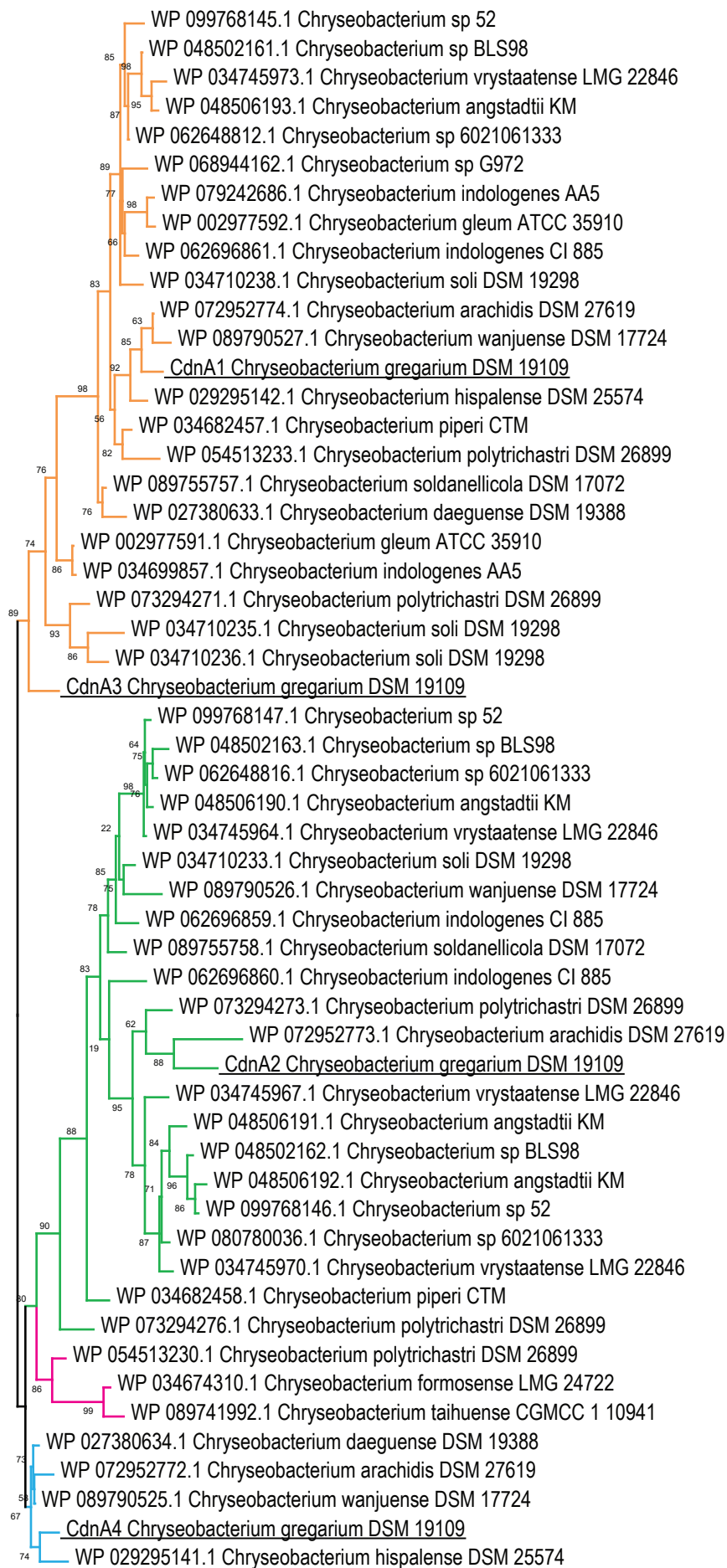

1

2

3

4

0.50

Supplement: Supplementary file 5 — Additional file 5. Figure S5. Phylogenetic analysis of chryseoviridin precursors. Approximate maximum likelihood phylogenetic tree was built using FastTree (WAG evolutionary model, gamma distributed site rates) (Price et al. [48]). Same program was used to calculated support values, which are indicated for each branch. Four distinct branches 1 to 4 are colored by orange, green, magenta and blue respectively. Precursors from Chryseobacterium gregarium DSM 19109 are underlined. [file 13062_2022_320_MOESM5_ESM.pdf]

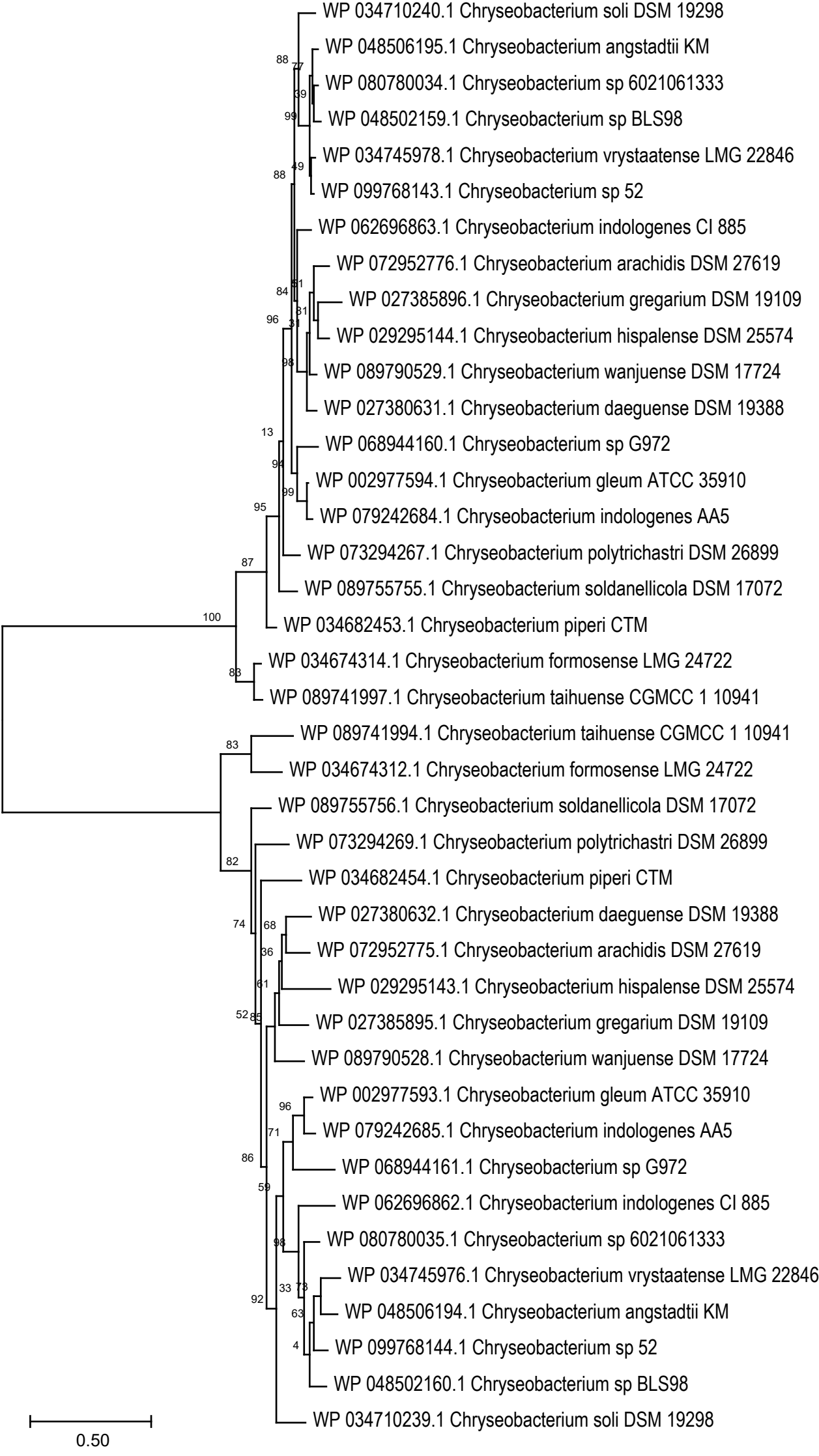

CdnC

CdnB

Supplement: Supplementary file 6 — Additional file 6. Figure S6. Phylogenetic analysis of ATP-grasps from chryseoviridin loci. Approximate maximum likelihood phylogenetic tree was built using FastTree (WAG evolutionary model, gamma distributed site rates) (Price et al. [48]). Same program was used to calculated support values, which are indicated for each branch. Two branches corresponding to two ATP-grasp proteins CdnA and CdnB encoded in chryseoviridin-like loci are indicated respectively. [file 13062_2022_320_MOESM6_ESM.pdf]

**A**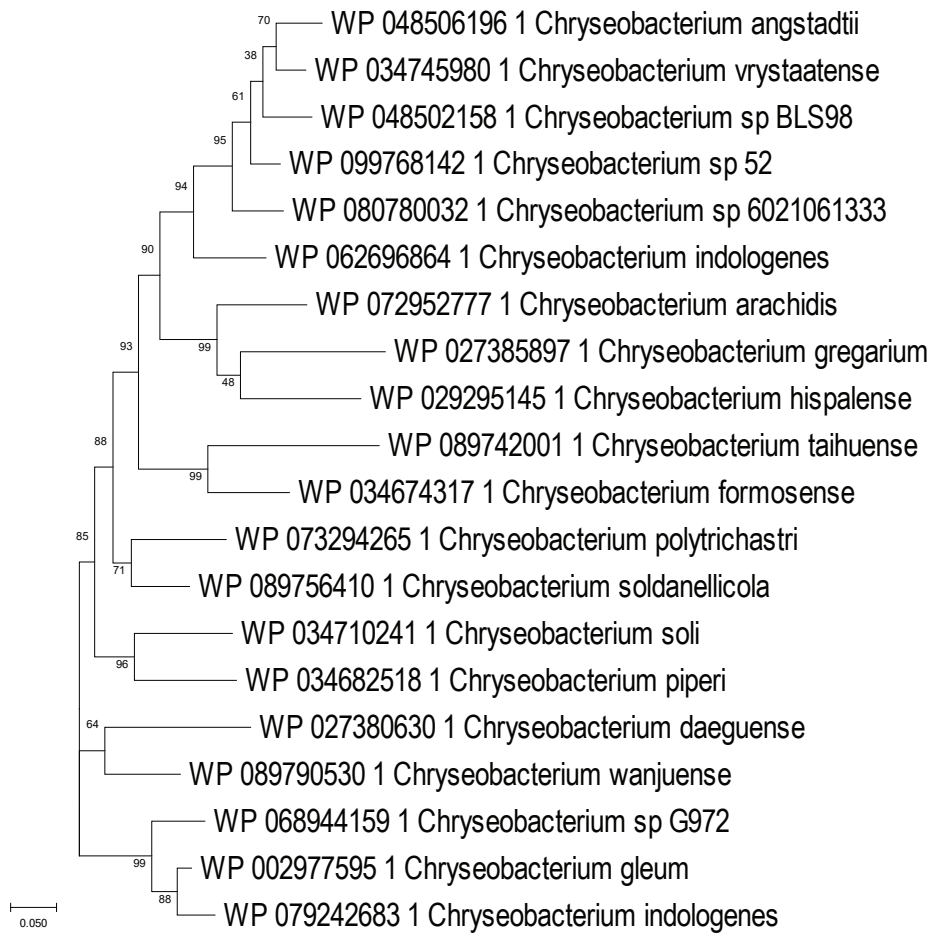**B**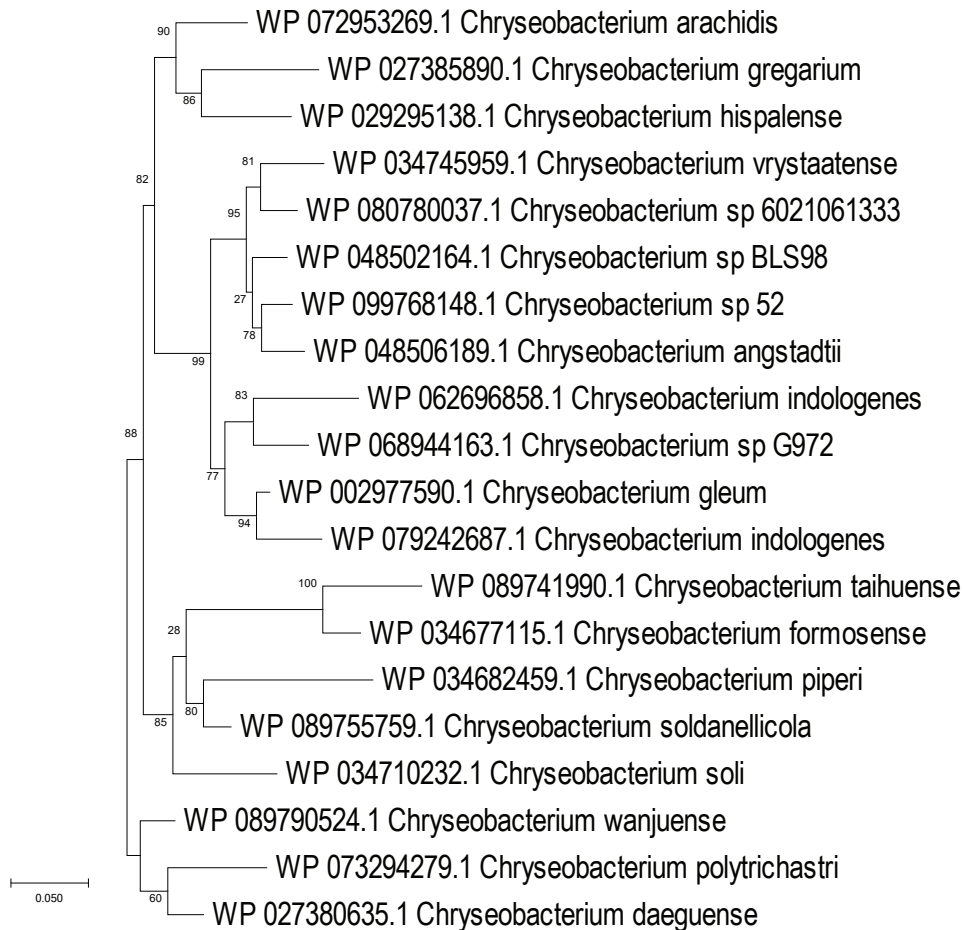

Supplement: Supplementary file 7 — Additional file 7. Figure S7. Phylogenetic analysis of flanking genes from chryseoviridin loci. A. Epimerase. B. Alpha/beta hydrolase. Approximate maximum likelihood phylogenetic trees were built using FastTree (WAG evolutionary model, gamma distributed site rates) (Price et al. [48]). Same program was used to calculated support values, which are indicated for each branch. [file 13062_2022_320_MOESM7_ESM.pdf]
