# Supplementary material for: Phylogenomic analysis of the diversity of graspetides and proteins involved in their biosynthesis
Source: Biol Direct. 2022 Mar 21;17:7. doi: 10.1186/s13062-022-00320-2 (PMC8939145; doi:10.1186/s13062-022-00320-2)
Supplement: Supplementary file 4 — Additional file 4. Figure S4. Comparison of pheganomycin BGC locus and partly similar locus from Streptomyces viridosporus T7A. Genes are shown by block arrows, roughly to scale. Homologous genes present in both loci connected by dashed lines and the percent of identical residues is indicated in red. A table with gene annotation for both loci is shown below. [file 13062_2022_320_MOESM4_ESM.pdf]

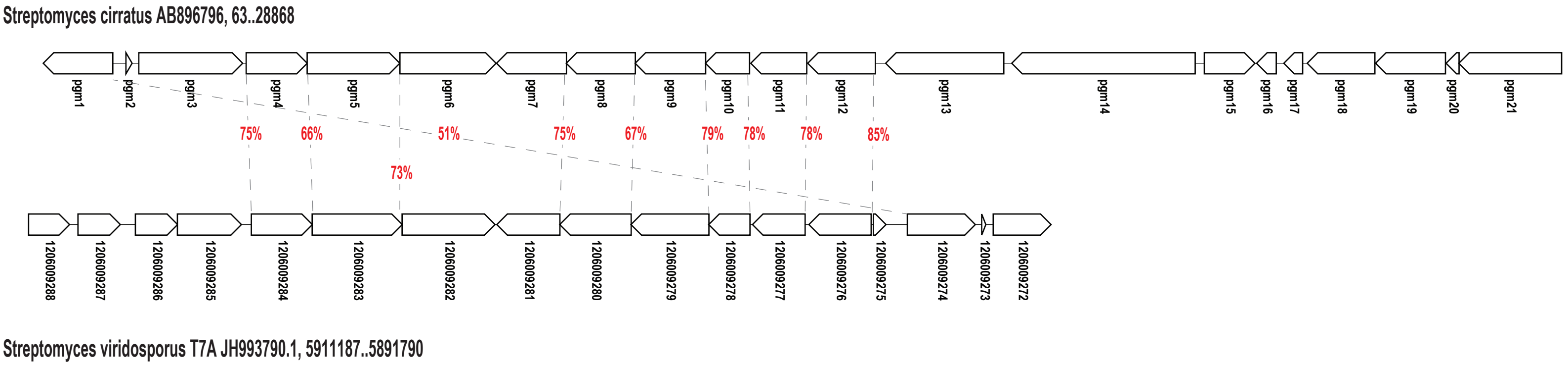

pgm1	peptide ligase
pgm2	Pheganomycin precursor
pgm3	radical SAM domain containing protein
pgm4	C-methyltransferase
pgm5	ABC transporter
pgm6	ABC transporter
pgm7	cytochrome P450
pgm8	aminotransferase
pgm9	enoyl-CoA hydratase like protein
pgm10	enoyl-CoA hydratase like protein
pgm11	chalcone and stilbene synthase like protein
pgm12	amidinotransferase
pgm13	hypothetical protein
pgm14	peptidase
pgm15	transcriptional regulator LysR
pgm16	hypothetical protein
pgm17	hypothetical protein
pgm18	permease
pgm19	non-ribosomal peptide synthetase C-domain containing protein
pgm20	non-ribosomal peptide synthetase T-domain containing protein
pgm21	non-ribosomal peptide synthetase A-domain containing protein

1206009272	ATP-grasp
1206009273	possible precursor
1206009274	PGM1-like ATP-grasp
1206009275	hypothetical protein
1206009276	amidinotransferase
1206009277	Chalcone and stilbene synthase family protein
1206009278	enoyl-CoA hydratase like protein
1206009279	enoyl-CoA hydratase like protein
1206009280	Aminotransferase
1206009281	Cytochrome P450
1206009282	ABC transporter permease
1206009283	ABC-type multidrug transport system, ATPase and permease component
1206009284	O-methyltransferase
1206009285	UDP-glycosyltransferases
1206009286	DNA binding domain of the transcription regulators TipA
1206009287	O-methyltransferase
1206009288	enoyl-CoA hydratase like protein

Supplementary Figure 4
